# Synthesis, Spectroscopic Characterization, and *In Vitro* Antibacterial Evaluation of Novel Functionalized Sulfamidocarbonyloxyphosphonates

**DOI:** 10.3390/molecules23071682

**Published:** 2018-07-10

**Authors:** Abdeslem Bouzina, Khaoula Bechlem, Hajira Berredjem, Billel Belhani, Imène Becheker, Jacques Lebreton, Marc Le Borgne, Zouhair Bouaziz, Christelle Marminon, Malika Berredjem

**Affiliations:** 1 Laboratory of Applied Organic Chemistry, Synthesis of Biomolecules and Molecular Modelling Group, Badji-Mokhtar—Annaba University, Box 12, 23000 Annaba, Algeria; bouzinaabdeslem@yahoo.fr (A.B.); bechlemkhaoula92@yahoo.com (K.B.); billelbelhani@yahoo.com (B.B.); 2 Laboratory of Applied Biochemistry and Microbiology, Department of Biochemistry, Badji-Mokhtar-Annaba University, Box 12, 23000 Annaba, Algeria; hajira.berredjem@univ-annaba.dz (H.B.); i_mene7@msn.com (I.B.); 3 CNRS, Université de Nantes, Chimie et Interdisciplinarité: Synthèse, Analyse, Modélisation (CEISAM), UMR CNRS 6230, 2 rue de la Houssinière, BP92208, CEDEX 3, 44322 Nantes, France; jacques.lebreton@univ-nantes.fr; 4 Université de Lyon, Université Lyon 1, Faculté de Pharmacie—ISPB, EA 4446 Bioactive Molecules and Medicinal Chemistry, SFR Santé Lyon-Est CNRS UMS3453—INSERM US7, CEDEX 8, 69373 Lyon, France; marc.le-borgne@univ-lyon1.fr (M.L.B.); zouhair.bouaziz@univ-lyon1.fr (Z.B.)

**Keywords:** sulfamides, phosphonates, carbamoylation, sulfamoylation, antibacterial activity

## Abstract

Several new sulfamidocarbonyloxyphosphonates were prepared in two steps, namely carbamoylation and sulfamoylation, by using chlorosulfonyl isocyanate (CSI), α-hydroxyphosphonates, and various amino derivatives and related (primary or secondary amines, β-amino esters, and oxazolidin-2-ones). All structures were confirmed by ^1^H, ^13^C, and ^31^P NMR spectroscopy, IR spectroscopy, and mass spectroscopy, as well as elemental analysis. Eight compounds were evaluated for their *in vitro* antibacterial activity against four reference bacteria including Gram-positive *Staphylococcus aureus* (ATCC 25923), and Gram-negative *Escherichia coli* (ATCC 25922), *Klebsiella pneumonia* (ATCC 700603), *Pseudomonas aeruginosa* (ATCC 27853), in addition to three clinical strains of each studied bacterial species. Compounds **1a**–**7a** and **1b** showed significant antibacterial activity compared to sulfamethoxazole/trimethoprim, the reference drug used in this study.

## 1. Introduction

The synthesis and reactivity of sulfamides (sulfonyl analogues of ureas) have attracted much interest in the last decades [1]. A large number of sulfamide derivatives have been reported to show biological activities such as anti-mycobacterial, anticonvulsant, anti-hypoglycemic, anticancer, and enzyme inhibition (e.g., carbonic anhydrase I, HIV-1 protease, elastase, carboxypeptidase A) [2,3,4,5,6,7,8,9]. These important compounds have been synthesized by various routes, most of them using the reaction of a sulfonyl chloride with ammonia or primary and secondary amines [10]. Another approach utilizes the amide exchange of a sulfamide by heating with an amine [11]. In parallel, many synthetic efforts have also focused on sulfonamide derivatives that have shown great potency to inhibit important biological targets such as cox-2, carbonic anhydrase (e.g., isoenzymes I, II, VII, IX), and NaV1.7, or to block, for example, the *Chlamydia* fatty acid synthesis [12,13,14,15,16].

**Figure 1:**
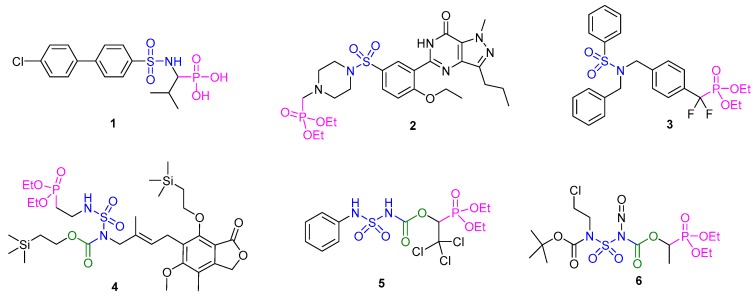
Structure of diverse sulfonamide and sulfamide derivatives containing a phosphonate-type group.

In addition, the extensive interest in the synthesis of bifunctional sulfonamide or sulfamide-phosphonate derivatives is due to their broad biological activities. In Figure 1, the structures of six bifunctional compounds are depicted. Biasone *et al.* [17] demonstrated that analogues of α-biphenylsulfonylamino 2-methylpropyl phosphonate **1** exhibit potency against several matrix metalloproteinases (MMPs). New sildenafil analogue **2** containing a phosphonate group in the 5′-sulfonamide moiety of the phenyl ring has shown promising *in vitro* PDE5 inhibitory activity [18]. Sulfonamide derivative **3** containing a single difluoromethylene phosphonate group has been discovered to be a potent inhibitor of protein tyrosine phosphatase PTP1B [19]. A series of phosphonate derivatives of mycophenolic acid **4** were described as anticancer, antiviral, and anti-inflammatory agents [20]. Compound **5** shows the highest insecticidal activity against plant pests [21]. It should be pointed out that to the best of our knowledge, it is the only example of compounds containing a sulfamidocarbonyloxyphosphonate moiety described in the literature. Finally, Winum *et al.* [22] reported the synthesis of sulfamide analogues of fotemustine **6** along with preliminary *in vitro* evaluation on two human melanoma cell lines.

**Figure 2:**
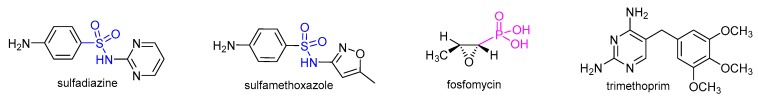
Structures of drugs approved for human use.

Since the 1930’s, sulfamide and sulfonamide derivatives have had a special place in the anti-infectious strategies and their therapeutic application continues to be investigated, as illustrated by this recent work on the use of sulfonamide agents against *Staphylococcus aureus* (SA) of the CNS [23]. They demonstrated that sulfadiazine and sulfamethoxazole (SMX) (Figure 2) exhibited strong activity against bacteria. Fosfomycin (Figure 2) is another well-known antibacterial agent with a structure containing a phosphonate motif and may be prescribed alone or in combination (e.g., with vancomycin). Unfortunately, year after year, increased bacterial resistance to sulfonamides/sulfamides [24] to the combination sulfamethoxazole-trimethoprim (SMX-TMP) [25] and to fosfomycin [26] has limited their use. Moreover, the appearance of multidrug resistant Gram-positive bacteria, in particular methicillin-resistant *Staphylococcus aureus* (MRSA) and vancomycin-resistant *Enterococci* (VRE), has become a major health problem [27]. So, new research on drug discovery needs to be intensively developed for designing new antibacterial agents.

**Figure 3:**
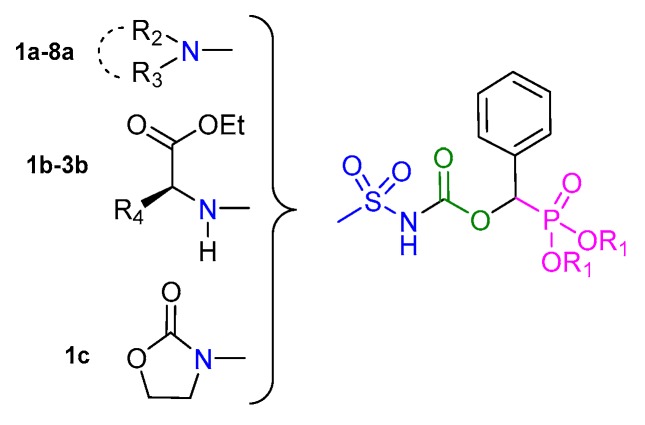
General formula of studied compounds.

In continuation of our interest in the preparation of sulfonamide and sulfamide derivatives [28,29,30,31], we decided to include both motifs, sulfamido and phosphonate, on each targeted compound and then to obtain new hybrids also containing an α-phenyl on the phosphonate methylene. For this preliminary study, we opted to select a set of various substituents -NR_2_R_3_ and -PO(OR_1_)_2_ in order to shape the first SAR trends in this series (Figure 3). To link these motifs, we chose a carbonyloxy-type spacer present in compounds **5** and **6** (Figure 1). The first-step reaction using chlorosulfonyl isocyanate and the corresponding α-hydroxyphosphonate-type intermediate allowed us to synthesize all *N*-chlorosulfonyl carbamate intermediates. In the second step, the key structural sequence, sulfamidocarbonyloxyphosphonate, was achieved directly from various amines. The antibacterial activity of eight phosphonate derivatives (**1a**–**7a** and **1b**) was studied against representative reference strains *Staphylococcus aureus* ATCC 25923, *Escherichia coli* ATCC 25922, *Klebsiella pneumoniae* ATCC 700603, and *Pseudomonas aeruginosa* ATCC 27853, as well as diverse clinical strains. Inhibition zones were performed by the disc diffusion method and the MIC values were determined by the dilution broth method [32]. The combination SMX-TMP, currently employed to treat bacterial infections, was used as the reference standard.

## 2. Results and Discussion

### 2.1. Chemistry

**Scheme 1:**
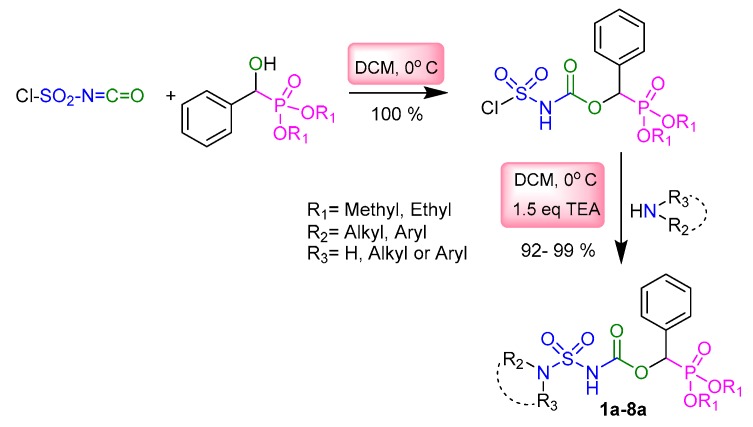
Synthesis of sulfamidocarbonyloxyphosphonates **1a**–**8a** from primary or secondary amines.

The synthetic route for the preparation of a novel series of sulfamidocarbonyloxyphosphonates **1a**–**8a** is outlined in Scheme 1. The synthesis was carried out in two steps. First, carbamoylation under anhydrous conditions of commercial chlorosulfonyl isocyanate with the corresponding α-hydroxyphosphonate (R_1_ = methyl or ethyl), easily prepared in a single step [33,34], quantitatively afforded the corresponding *N*-chlorosulfonyl carbamate intermediate. Reaction with various primary or secondary amines in the presence of triethylamine at 0 °C then gave the target compounds **1a**–**8a** in excellent yields (92–99%) within 60–90 min (Table 1).

**Scheme 2:**
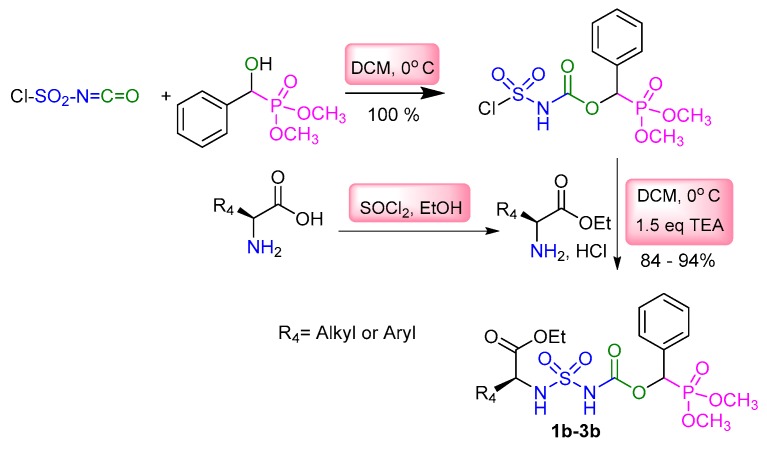
Synthesis of sulfamidocarbonyloxyphosphonates **1b**–**3b** from amino acid esters.

To increase the scope of this reaction, we synthesized other sulfamidocarbonyloxyphosphonates using diverse (*S*)-amino acid esters (Scheme 2). The isolated yields of the products **1b**–**3b**, obtained as a mixture of diastereoisomers (Table 2), were in the range of 84–94% yield after 90 min of reaction.

**Scheme 3:**
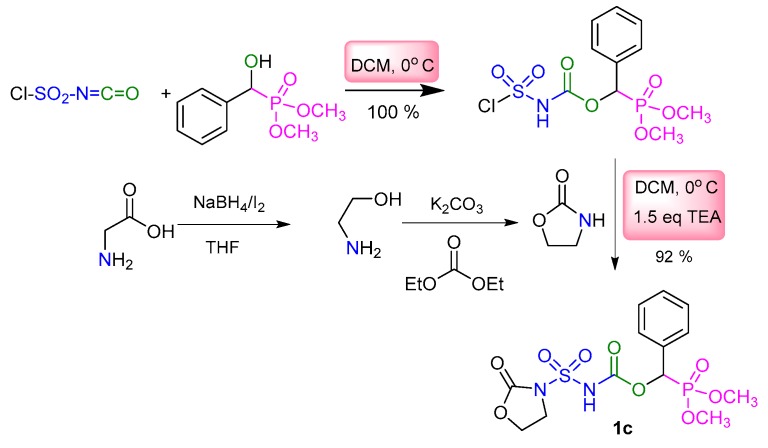
Synthesis of sulfamidocarbonyloxyphosphonate **1c** from oxazolidin-2-one.

These satisfactory and encouraging results have prompted us to develop a third subseries (Scheme 3, Table 3), by using oxazolidin-2-one as a building block in order to synthesize new potential bioactive molecules. The new compound **1c** was obtained with a very good yield (92%).

Spectrometric methods confirmed the structures of all the sulfamidocarbonyloxyphosphonates synthesized. Their physicochemical and analytical data are depicted in Table 1, Table 2 and Table 3. The FT-IR spectrum showed the characteristic signals of the three functions, namely the carbamate NH stretching at 3300–3250 cm^−1^ and its C=O stretching at 1750–1730 cm^−1^, the phosphonate group at 1255–1234 cm^−1^, and the sulfamide group with its two signals at 1185–1118 cm^−1^ and 1384–1356 cm^−1^. The molecular peak [M + H]^+^ obtained by ESI-MS was always present and corresponded to each synthesized compound. NMR spectra were recorded using CDCl_3_ as the solvent and are available in the supplementary material part. The ^1^H spectrum always exhibited a dramatically deshielded doublet at 6 ppm corresponding to the COOC**H**(Ph)POOR proton with its expected coupling constant ^2^*J*_H-P_ frequently around 12–14 Hz. The two methoxy groups of the phosphonate appeared as two separate doublets (^3^*J*_H-P_~10 Hz) at 3.5 and 3.7 ppm, while the NH of the carbamate appeared as a broad singlet at δ 8–11 ppm. The ^13^C spectrum was also characteristic due to the expected doublets related to the presence of the phosphorus (*J*_C-P_ couplings): (i) the methoxy of the phosphonates at 54 ppm (^2^*J*_C-P_~7–8 Hz), and (ii) the aromatic ring (^3^*J*_C-P_~6 Hz and ^4^*J*_C-P_~1–3 Hz) [35,36]. The ^13^C chemical shifts are particular, as the carbonyl of the carbamate at 150 ppm (doublet with a ^3^*J*_C-P_ = 12 Hz coupling constant) and the greatly deshielded COO**C**H(Ph)POOR carbon at 70 ppm (doublet with a ^1^*J*_C-P_ = 170 Hz coupling constant).

To determine the initial interest of these novel functionalized sulfamidocarbonyloxyphosphonates as antibacterial agents, we only selected eight derivatives (including seven from the first sub-series) for testing their potency against sixteen bacterial strains. This first biological study can confirm the interest to modulate such a scaffold.

### 2.2. In Vitro Antibacterial Evaluation of Sulfamidocarbonyloxyphosphonates

A total of twelve clinical strains of Gram-positive and Gram-negative bacteria and four control strains (*S. aureus* ATCC 25923, *E. coli* ATCC 25922, *P. aeruginosa* ATCC 27853, and *K. pneumoniae* ATCC 700603) were used to investigate the antibacterial activity. The eight tested sulfamidocarbonyloxyphosphonate derivatives (compounds **1a**–**7a** and **1b**) showed antibacterial activity with a varying degree of inhibitory effect on the growth of the bacterial strains (Table 4 and Table 5).

The disk diffusion is just a qualitative method to determine whether a particular bacterium is susceptible to the action of a specific antimicrobial agent. The presence or the absence of a clear region around the disk is an indication of the inhibition or lack of inhibition of the bacterial growth. Then, the size of the zone of inhibition indicates the degree of sensitivity of bacteria to an antimicrobial drug. We could use the terms “resistant, intermediate, and sensitive” to discuss the results obtained. As shown in Table 4, the diameters of the inhibition zone (DIZ) of the tested compounds against the bacteria strains ranged from 12–26 mm. The values obtained with the positive control sulfamethoxazole-trimethoprim (SXT) ranged between 17 and 22 mm for both clinical and control strains. Furthermore, some *P. aeruginosa* and *K. pneumoniae* strains (*P. aeruginosa* 1, *K. pneumoniae* 1 and 3) were resistant (R) towards SXT. It should be noted that *P. aeruginosa* is known to be a multidrug resistant bacteria due to its remarkable ability of acquiring mechanisms of resistance to some antimicrobial agents.

Tested sulfamidocarbonyloxyphosphonate derivatives were more active toward Gram-negative bacteria than Gram-positive ones. Compound **4a** was inactive on all three clinical strains of *S. aureus* (only DIZ = 12 mm for *S. aureus* ATCC 25923). Compounds **2a**, **3a**, **6a**, **7a**, and **1b** exerted intermediate activity on *S. aureus* (12 ≤ DIZ ≤ 16 mm). The best activities on *S. aureus* (DIZ > 16 mm, result reported as sensitive) were observed with compounds **1a** (*S. aureus* 3) and **5a** (*S. aureus* 1 and 3) with zone sizes of 17, 18, and 17 mm, respectively. Nevertheless, SXT seems to give better results, with zone sizes of between 18 and 22 mm. On *E. coli* strains, all tested compounds and STX gave results with inhibition zones between 17 and 25 mm. Among them, compounds **3a** and **4a** were the most active molecules against *E. coli*, with inhibition diameters of 25 mm for *E. coli* ATCC 25922 and 24 mm for *E. coli* 2. For *P. aeruginosa* strains, the inhibition zones were between 17 and 26 mm. Their susceptibility was really marked with compounds **1a**, **4a**, and **5a**, with zone sizes between 18 and 26 mm. Compound **1a** showed the best activity against strains *P. aeruginosa* ATCC 27853 and *P. aeruginosa* 2, with an inhibition diameter of 26 mm. SXT exhibited less activity against the four *P. aeruginosa* strains tested. For example, in the cases of *P. aeruginosa* ATCC 27853 and *P. aeruginosa* 2, the inhibition zones of STX were equal to 17 and 20 mm, respectively. Concerning *K. Pneumoniae*, all tested sulfamidocarbonyloxyphosphonate derivatives were globally active, with inhibition zones superior to 15 mm. The best activity was obtained with compound **4a**, with inhibition zones of 24 and 25 mm for clinical strains.

After the evidence of *in vitro* antibacterial activity against the tested strains in the disk diffusion test, the Minimum Inhibitory Concentration (MIC) values were determined. As shown in Table 5, most derivatives exhibited low MIC values against the different strains of bacteria employed when compared with STX (MIC = 25 µg/mL). All the tested compounds showed the best MIC values against *E. coli* and *P. aeruginosa* strains, ranging between 0.5 and 32 µg/mL. In particular, compounds **1a**, **3a**, and **6a** exerted the most intense activity, especially on *P. aeruginosa*, with MIC values ranging between 0.5 and 1 µg/mL for **1a** and 1 and 4 µg/mL for **3a** and **6a**. As regards compound **4a**, it was very active against *E. coli* strains, with MIC values between 0.5 and 4 µg/mL. For *K. pneumoniae*, the best results were obtained with compounds **1a** and **4a**, with MIC values in the range of 4 to 8 µg/mL and 2 to 16 µg/mL, respectively. Concerning *S. aureus* strains, all MIC values were superior to 64 µg/mL, except for compound **1b** (15 ≤ MIC ≤ 18 µg/mL).

Overall, our results showed that the sulfamidocarbonyloxyphosphonates possessed a good concentration dependent antibacterial activity, especially against the tested Gram-negative bacteria at MIC values ranging between 0.5–32 µg/mL for compound **1a**, and 0.5 and 16 µg/mL for compound **4a**. Among the eight compounds tested, only compound **1b** exerted antibacterial activity against *S. aureus*.

## 3. Materials and Methods

### 3.1. General Information

All chemicals and solvents were purchased from common commercial sources and were used as received without any further purification. All reactions were monitored by TLC on silica Merck 60 F254 percolated aluminum plates and were developed by spraying with ninhydrin solution. Column chromatography was performed with Merck silica gel (230–400 mesh). Proton nuclear magnetic resonance (^1^H NMR) spectra were recorded on Bruker or Jeol spectrometers at 400 MHz. Chemical shifts are reported in δ units (ppm) with TMS as the reference (δ 0.00). All coupling constants (*J*) are reported in Hertz. Multiplicity is indicated by one or more of the following: b (broad), s (singlet), d (doublet), t (triplet), q (quartet), dd (doublet of doublet), and m (multiplet). The Carbon nuclear magnetic resonance (^13^C NMR) spectra were recorded on Bruker (Reinstetten, Germany) or Jeol (JNM-ECS400 (Tokyo, Japan) spectrometers at 100.62 MHz. Chemical shifts are reported in δ units (ppm) and coupling constants (*J*) are reported in Hertz. Phosphorus nuclear magnetic resonance (^31^P NMR) spectra and Fluor (^19^F NMR) nuclear magnetic resonance spectra were recorded on a Bruker spectrometer at 161.98 MHz and 316.48 MHz, respectively. Infrared spectra were recorded on a Perkin Elmer 600 (Waltham, Massachusetts, USA) spectrometer. The Mass spectra were recorded on a shimadzu QP 1100 Ex mass spectrometer operating at an ionization potential of 70 eV. Elemental analysis was recorded on a EURO E.A. 3700 apparatus. All melting points were recorded on a Büchi B-545 (Taufkirchen, Germany) apparatus in open capillary tubes.

Ultrasound assisted reactions were carried out using a FUNGILAB ultrasonic bath (Barcelona, Spain) with a frequency of 40 kHz and a nominal power of 250 W. The reactions were carried out in an open glass tube (diameter: 25 mm; thickness: 1 mm; volume: 20 mL) at room temperature.

### 3.2. Typical Experimental Procedure for the Synthesis of Sulfamidocarbonyloxyphosphonates ***1a**–**8a***, ***1b**–**3b***, and ***1c***

α-Hydroxyphosphonates were synthesized in 94% overall yield starting from benzaldehyde and trialkylphosphites under ultrasound irradiation according to the procedure described in reference [34].

A solution of α-hydroxyphosphonate (1.1 equiv) in anhydrous CH_2_Cl_2_ (5 mL) was added dropwise to a stirring solution of chlorosulfonyl isocyanate (CSI) (1 equiv) in anhydrous CH_2_Cl_2_ (5 mL) at 0 °C over a period of 20 min. The resulting solution was transferred to a mixture of primary or secondary amine (1.1 equiv) or amino acid ester or oxazolidin-2-one in anhydrous CH_2_Cl_2_ (10 mL) in the presence of triethylamine (1.1–1.5 equiv). The reaction mixture was stirred at 0 °C for less than 1–2 h, and then neutralized by adding a solution of aqueous HCl 0.1 M to pH 7. The organic layer was extracted, washed with water, dried over anhydrous sodium sulfate, filtered, and concentrated *in vacuo*. The pure products were crystallized in a mixture of diethyl ether/*n*-hexane (1.5:1) at 6 °C overnight. The pure sulfamidocarbonyloxyphosphonates were finally filtered and dried in excellent yields.

*(Dimethoxyphosphoryl)(phenyl)methyl (N-benzylsulfamoyl)carbamate* (**1a**)*.* White powder, 99% yield, m.p. 131–133 °C, R*_f_* = 0.43 (CH_2_Cl_2_/MeOH, 90:10). IR (KBr, cm^−1^): 3365, 3298, 1733, 1481, 1364, 1249, 1170. ^1^H-NMR (400 MHz, CDCl_3_) δ: 3.57 (d, 3H, ^3^*J_H-P_* = 10.4 Hz, C**H**_3_-OP), 3.77 (d, 3H, ^3^*J_H-P_* = 10.8 Hz, C**H**_3_-OP), 4.11 (dd, 1H, *J*_1_ = 13.6 Hz, *J*_2_ = 5.4 Hz, C**H**-N), 4.23 (dd, 1H, *J*_1_ = 14.0 Hz, *J*_2_ = 5.6 Hz, C**H**-N), 5.61 (bs, 1H, N**H**-SO_2_), 6.00 (d, 1H, ^2^*J_H-P_* = 12.0 Hz, CH*-OP), 7.18–7.28 (m, 5H, **H**-Ar), 7.36–7.42 (m, 3H, **H**-Ar), 7.47–7.53 (m, 2H, **H**-Ar), 8.90 (bs, 1H, N**H**-C=O). ^13^C-NMR (100.62 MHz, CDCl_3_) δ: 48.15 (CH_2_), 54.19 (d, *J*_C-P_ = 7 Hz, POCH_3_), 54.48 (d, *J*_C-P_ = 7 Hz, POCH_3_), 72.52 (d, *J*_C-P_ = 172 Hz, CH*-OP), 128.03 (2C, d, *J*_C-P_ = 6 Hz), 128.18 (2C), 128.34 (2C), 128.54, 128.96 (2C, d, *J*_C-P_ = 4 Hz), 129.58, 132.41, 135.54, 150.49 (d, J_C-P_ = 11 Hz, C=O). ^31^P-NMR (161.98 MHz, CDCl_3_) δ: 19.10. Anal. Calc. for C_17_H_21_N_2_O_7_PS: C 47.66, H 4.94, N 6.54, S 7.48. Found: C 47.71, H 4.89, N 6.52, S 7.44%. ESI-MS: (*m*/*z*) = 429.1 [M + H]^+^.

*(Dimethoxyphosphoryl)(phenyl)methyl(N-(2-methoxyphenyl)sulfamoyl)carbamate* (**2a**)*.* White powder, 98% yield, m.p. 137–139 °C, R*_f_* = 0.40 (CH_2_Cl_2_/MeOH, 90:10). IR(KBr, cm^−1^): 3342, 3275, 1733, 1489, 1361, 1252, 1136. ^1^H-NMR (400 MHz, CDCl_3_) δ: 3.50 (d, 3H, ^3^*J_H-P_*= 10.8 Hz, C**H**_3_-OP), 3.54 (s, 3H, C**H**_3_-O), 3.62 (d, 3H, ^3^*J_H-P_* = 10.8 Hz, C**H**_3_-OP), 5.94 (d, 1H, ^2^*J_H-P_* = 14.0 Hz, CH*-OP), 6.75 (dd, 1H, *J*_1_ = 8.0 Hz, *J*_2_ = 1.2 Hz, **H***_ortho_*-Ar OMe), 6.84 (td, 1H, *J*_1_ = 7.6 Hz, *J*_2_ = 1.2 Hz, **H***_metha_*-Ar), 7.07 (td, 1H, *J*_1_ = 6.8 Hz, *J*_2_ = 1.2 Hz, **H**-Ar), 7.31–7.39 (m, 5H, **H**-Ar), 7.43 (dd, 1H, *J*_1_ = 8.0 Hz, *J*_2_ = 1.6 Hz, **H*_ortho_***-Ar NH), 7.55 (bs, 1H, N**H**-SO_2_), 9.85 (bs, 1H, N**H**-C=O). ^13^C-NMR (100.62 MHz, CDCl_3_) δ: 54.16 (d, *J*_C-P_ = 7 Hz, POCH_3_), 54.26 (d, *J*_C-P_ = 7 Hz, POCH_3_), 55.79 (OCH_3_), 72.20 (d, *J*_C-P_ = 174 Hz, CH*-OP), 111.09, 120.87, 121.04, 121.37, 125.95, 128.09 (2C, d, *J*_C-P_ = 6 Hz), 128.86, 129.33 (2C, d, *J*_C-P_ = 3 Hz), 132.52, 149.73, 150.09 (d, *J*_C-P_ = 12 Hz, C=O). ^31^P-NMR (161.98 CDCl_3_) δ: 18.81. Anal. Calc. for C_17_H_21_N_2_O_8_PS: C 45.95, H 4.76, N 6.30, S 7.22. Found: C 45.90, H 4.81, N 6.28, S 7.26%. ESI-MS: (*m*/*z*) = 445.1 [M + H]^+^.

*(Dimethoxyphosphoryl)(phenyl)methyl(morpholinosulfonyl)carbamate* (**3a**)*.* White powder, 98% yield, m.p. 144–146 °C, R*_f_* = 0.47 (CH_2_Cl_2_/MeOH, 90:10). IR (KBr, cm^−1^): 3447, 3297, 1732, 1481, 1361, 1247, 1185, 769, 687. ^1^H-NMR (400 MHz, CDCl_3_) δ: 3.29–3.31 (m, 4H, 2 C**H**_2_-N), 3.56 (d, 3H, ^3^*J_H-P_* = 10.4 Hz, C**H**_3_-OP), 3.65–3.67 (m, 4H, 2 C**H**_2_-O), 3.84 (d, 3H,^3^*J_H-P_* = 10.8 Hz, C**H**_3_-OP), 6.02 (d, 1H, ^2^*J_H-P_* = 13.6 Hz, CH*-OP), 7.37–7.40 (m, 3H, **H**-Ar), 7.51–7.55 (m, 2H, **H**-Ar), 9.92 (bs, 1H, N**H**-C=O). ^13^C-NMR (100.62 MHz, CDCl_3_) δ: 46.70 (2C, CH_2_-N), 54.23 (d, *J*_C-P_ = 7 Hz, POCH_3_), 54.38 (d, *J*_C-P_ = 7 Hz, POCH_3_), 66.32 (2C, CH_2_-O), 72.09 (d, *J*_C-P_ = 174 Hz, CH*-OP), 128.16 (2C, d, *J*_C-P_ = 6 Hz), 128.92 (2C, d, *J*_C-P_ = 1 Hz), 129.56 (d, *J*_C-P_ = 3 Hz), 132.46, 150.72 (d, *J*_C-P_ = 12 Hz, C=O). ^31^P-NMR (161.98 CDCl_3_) δ: 18.93. Anal. Calc. for C_14_H_21_N_2_O_8_PS: C 41.18, H 5.18, N 6.86, S 7.85. Found: C 41.22, H 5.23, N 6.83, S 7.81%. ESI-MS: (*m*/*z*) = 409.1 [M + H]^+^.

*(Dimethoxyphosphoryl)(phenyl)methyl(N-(3-fluorophenyl)sulfamoyl)carbamate* (**4a**)*.* White powder, 96% yield, m.p. 136–138 °C, R*_f_* = 0.41 (CH_2_Cl_2_/MeOH, 90:10). IR (KBr, cm^−1^): 3311, 3297, 1758, 1477, 1355, 1251, 1166. ^1^H-NMR (400 MHz, CDCl_3_) δ: 3.62 (dd, 3H, *J_1_* = 38.8 Hz, *J_2_* = 10.4 Hz, C**H**_3_-O), 3.72 (dd, 3H, *J_1_* = 10.4 Hz, *J_2_* = 1.2 Hz, CH_3_-O), 5.95 (d, 1H, *J* = 13.6, CH*-O), 6.88–7.04 (m, 3H, **H**-Ar), 7.19–7.41 (m, 6H, **H**-Ar). ^13^C-NMR (100.62 MHz, CDCl_3_) δ: 54.85, 54.90, 71.86, 73.21, 128.14, 128.30, 129.23, 129.65, 12.91, 131.15, 131.75, 134.19, 134.56, 138.25, 138.45, 150.36. ^31^P-NMR (161.98 CDCl_3_) 20.61. ^19^F-NMR (316.48 MHz, CDCl_3_) δ: −111.62. Anal. Calc. for C_16_H_18_FN_2_O_7_PS: C 44.45, H 4.20, N 6.48, S 7.42. Found: C 44.40, H 4.23, N 6.52, S 7.41%. ESI-MS: (*m*/*z*) = 433.1 [M + H]^+^.

*(Diethoxyphosphoryl)(phenyl)methyl(N-phenylsulfamoyl)carbamate* (**5a**)*.* White powder, 96% yield, m.p. 187–189 °C, R*_f_* = 0.42 (CH_2_Cl_2_/MeOH, 90:10). IR (KBr, cm^−1^): 3447, 3297, 1733, 1481, 1384, 1247, 1185. ^1^H-NMR (400 MHz, CDCl_3_) δ: 1.03 (t, 3H, *J =* 7.0 Hz, C**H**_3_), 1.30 (t, 3H, *J* = 7.0 Hz, C**H**_3_), 3.59–3.68 (m, 1H, C**H**_2_-O), 3.82–3.90 (m, 1H, C**H**_2_-O), 4.07–4.17 (m, 2H, C**H**_2_-O), 5.82 (d, 1H, *J* = 8.8 Hz, CH*OP), 6.47 (s, 1H, N**H**-SO_2_), 6.80 (dd, 2H, *J*_1_ = 8.8 Hz, *J*_2_ = 1.2 Hz, **H**-Ar), 7.02 (t, 1H, *J* = 7.6 Hz, **H**-Ar), 7.15 (t, 2H, *J* = 7.6 Hz, **H**-Ar), 7.20–7.26 (m, 5H, **H**-Ar).^13^C-NMR (100.62 MHz, CDCl_3_) δ: 16.32 (CH_3_), 16.59 (CH_3_), 63.96 (CH_2_), 64.10 (CH_2_), 72.46 (d, *J*_C-P_ = 170 Hz, CH*-OP), 119.77 (2C), 124.46, 128.31 (2C, d, *J*_C-P_ = 6 Hz), 128.76 (2C), 128.90 (2C), 129.32, 134.25, 136.86, 150.40 (d, *J*_C-P_ = 16 Hz, C=O). ^31^P-NMR (161.98 MHz, CDCl_3_) δ: 19.61. Anal. Calc. for C_18_H_23_N_2_O_7_PS: C 48.87, H 5.24, N 6.33, S 7.25. Found: C 48.93, H 5.21, N 6.28, S 7.26%. ESI-MS: (*m*/*z*) = 443.1 [M + H]^+^.

*(Dimethoxyphosphoryl)(phenyl)methyl(3,4-dihydroisoquinolin-2(1H)-yl)sulfonylcarbamate* (**6a**). Color powder, 94% yield, m.p. 153–155 °C, R*_f_* = 0.49 (CH_2_Cl_2_/MeOH, 90:10). IR (KBr, cm^−1^): 3258, 1750, 1360, 1454, 1234, 1120. ^1^H-NMR (400 MHz, CDCl_3_) δ: 2.87 (t, 2H, *J* = 6.0 Hz, C_Ar_-**CH_2_**-CH_2_), 3.55 (d, 3H, ^3^*J_H-P_* = 10.0 Hz, C**H**_3_-OP), 3.60 (t, 2H, *J* = 6.0 Hz, CH_2_-**CH_2_**-N), 3.75 (d, 3H, ^3^*J_H-P_* = 10.0 Hz, C**H**_3_-O), 4.52 (s, 2H, C_Ar_-**CH_2_**-N), 6.00 (d, 1H, ^2^*J_H-P_* = 12.0 Hz, CH*-O), 7.01–7.08 (m, 2H, **H**-Ar), 7.14–7.16 (m, 2H, **H**-Ar), 7.33–7.36 (m, 3H, **H**-Ar), 7.48-7.51 (m, 2H, **H**-Ar), 9.91 (s, 1H, N**H**-C=O). ^13^C-NMR (100.62 MHz, CDCl_3_) δ: 28.38 (CH_2_), 44.47 (NCH_2_), 47.78 (NCH_2_), 54.63 (2C, POCH_3_), 72.04 (d, *J*_C-P_ = 178 Hz, CH*-OP), 126.4, 126.6, 127.1, 128.6 (2C), 128.8, 129.60 (2C), 129.80, 131.41, 132.4, 133.2, 150.95 (d, *J*_C-P_ = 16 Hz, C=O). ^31^P-NMR (161.98 MHz, CDCl_3_) δ: 18.76. Anal. Calc. for C_19_H_23_N_2_O_7_PS: C 50.22, H 5.10, N 6.10, S 7.06. Found: C 50.19, H 5.15, N 6.16, S 7.10%. ESI-MS: (*m*/*z*) = 453.2 [M − H]^+^.

*(Dimethoxyphosphoryl)(phenyl)methyl(4-phenylpiperazin-1-yl)sulfonylcarbamate* (**7a**). White powder, 93% yield, m.p. 152–154 °C, R*_f_* = 0.50 (CH_2_Cl_2_/MeOH, 90:10). IR (KBr, cm^−1^): 3337, 1741, 1449, 1360, 1248, 1167. ^1^H-NMR (400 MHz, CDCl_3_) δ: 3.10–3.40 (m, 4H, 2 **CH_2_**-N-SO_2_), 3.42–3.62 (m, 4H, 2 **CH_2_**-N-C_Ar_), 3.67 (d, 3H, *J* = 10.6 Hz, C**H**_3_-O), 3.75 (d, 3H, *J* = 10.8 Hz, C**H**_3_-O), 6.05 (d, 1H, ^2^*J_H-P_* = 14.0 Hz, CH^*^-O), 6.80–6.96 (m, 3H, **H**-Ar), 7.25–7.40 (m, 5H, **H**-Ar), 7.45–7.56 (m, 2H, **H**-Ar). ^13^C-NMR (100.62 MHz, CDCl_3_) δ: 46.78 (2C, NCH_2_), 48.90 (2C, NCH_2_), 54.61 (d, *J*_C-P_ = 7 Hz, POCH_3_), 54.62 (d, *J*_C-P_ = 7 Hz, POCH_3_), 70.86 (d, *J*_C-P_ = 142.4 Hz, CH*-OP), 117.37 (2C), 120.93, 127.87 (2C, d, *J*_C-P_ = 5 Hz), 128.57, 128.92 (2C), 129.65 (2C), 132.49, 136.81, 151.27 (d, *J*_C-P_ = 12 Hz, C=O). ^31^P-NMR (161.98 MHz, CDCl_3_) δ: 19.82. Anal. Calc. for C_20_H_26_N_3_O_7_PS: C 49.68, H 5.42, N 8.69, S 6.63. Found: C 49.73, H 5.46, N 8.65, S 6.67%. ESI-MS: (*m*/*z*) = 482.3 [M − H]^+^.

*(Diethoxyphosphoryl)(phenyl)methyl(N-propylsulfamoyl)carbamate* (**8a**)*.* White powder, 97% yield, m.p. 151–153 °C, R*_f_* = 0.43 (CH_2_Cl_2_/MeOH, 90:10). IR (KBr, cm^−1^): 3369, 3061, 1758, 1475, 1355, 1240, 1156, 763, 697. ^1^H-NMR (400 MHz, CDCl_3_) δ: 0.72 (t, 3H, *J* = 8.8 Hz, C**H**_3_-Pr), 1.09 (t, 3H, *J* = 9.4 Hz, C**H**_3_-OEt), 1.12–1.27 (m, 2H, C**H**_2_-Pr), 1.37 (t, 3H, *J* = 9.4 Hz, C**H**_3_-OEt), 2.48–2.59 (m, 1H, C**H**_2_-N), 2.78–2.87 (m, 1H, C**H**_2_-N), 3.67–4.05 (m, 2H, **CH**_2_-OP), 4.25 (1H, m, NH), 4.74 (dq, 2H, ^3^*J_H-P_* = 11.8 Hz, ^3^*J*_H-H_ = 7.5 Hz, C**H**_2_-OP), 6.00 (dd, 1H, ^2^*J_H-P_* = 11.3 Hz, *J* = 8.8 Hz, CH^*^-O), 7.35–7.38 (m, 3H, **H**-Ar), 7.50–7.52 (m, 2H, **H**-Ar). Anal. Calc. for C_15_H_25_N_2_O_7_PS: C 44.11, H 6.17, N 6.86, S 7.85. Found: C 44.29, H 6.79, N 6.91, S 7.80%. ESI-MS: (*m*/*z*) = 409.2 [M + H]^+^.

*(SR) and (SS)-Ethyl-2-((N-(((dimethoxyphosphoryl)(phenyl)methoxy)carbonyl)sulfamoyl)amino) -4-methylpentanoate* (**1b**)*.* White powder, 91% yield; m.p. 118–120 °C, R*_f_* = 0.39 (CH_2_Cl_2_/MeOH, 90:10). IR (KBr, cm^−1^): 3274, 1747 (l), 1470, 1371, 1251, 1164. ^1^H-NMR (400 MHz, CDCl_3_) δ: 0.81–0.87 (m, 12H, C**H**_3_-CH*_isop_*), 1.05–1.35 (m, 6H, O-CH**_2_**-C**H**_3_), 1.36–1.60 (m, 4H, 2C**H***_isop_*+ 1C**H**_2_-CH*_isop_*), 1.20 (m, 2H, 1C**H**_2_-CH*_isop_*), 3.51 (d, 3H, *J* = 10.6 Hz, C**H**_3_-O), 3.52 (d, 3H, *J* = 10.6 Hz, C**H**_3_-O), 3.60–3.75 (m, 1H, CH*-NH), 3.75–3.99 (m, 3H, -O-**CH_2_**-CH_3_ + CH*-NH), 3.78 (d, 6H, *J* = 10.8 Hz, C**H**_3_-O), 4.00–4.25 (m, 2H, -O-**CH_2_**-CH_3_), 5.79 (bs, 1H, N**H**-SO_2_), 5.96 (d, 1H, *J* = 13.9 Hz, CH^*^-O), 6.00 (d, 1H, *J* = 14.3 Hz, CH^*^-O), 6.21 (bs, 1H, N**H**-SO_2_), 7.32–7.40 (m, 6H, **H**-Ar), 7.50-7.56 (m, 4H, **H**-Ar), 9.86 (bs, 1H, N**H**-C=O). ^13^C-NMR (100.62 MHz, CDCl_3_) δ: 14.04 (2CH_3_), 22.76 (4C), 24.37 (2C), 41.95(2C), 54.19 (2POCH_3_), 54.44 (2POCH_3_), 55.55 (2C), 61.63 (2OCH_2_), 72.13 (2C, d, *J*_C-P_ = 142.4 Hz, CH*-OP), 128.03 (4C), 128.12 (2C), 128.75 (4C, d, *J*_C-P_ = 5 Hz), 132.50 (2C), 150.60 (2C, d, *J*_C-P_ = 2 Hz, C=O), 172.01 (2C=O). ^31^P-NMR (161.98 MHz, CDCl_3_) δ: 21.61. Anal. Calc. for C_18_H_29_N_2_O_9_PS: C 45.00, H 6.08, N 5.83, S 6.67. Found: C 45.07, H 6.04, N 5.81, S 6.72%. ESI-MS: (*m*/*z*) = 481.1 [M + H]^+^.

*(SR) and (SS)-Ethyl-2-((N-(((dimethoxyphosphoryl)(phenyl)methoxy)carbonyl)sulfamoyl)amino) -3-phenylpropanoate* (**2b**)*.* White powder, 94% yield; m.p. 125–127 °C, R*_f_* = 0.41 (CH_2_Cl_2_/MeOH, 90:10). IR (KBr, cm^−1^): 3279, 1744 (l), 1455, 1373, 1249, 1162. ^1^H-NMR (400 MHz, CDCl_3_) δ: 1.01 (t, 3H, *J* = 7.6 Hz, C**H**_3_-CH_2-_O), 1.02 (t, 3H, *J* = 7.6 Hz, C**H**_3_-CH_2-_O), 2.85–3.15 (m, 4H, C**H**_2_-Ar), 3.49 (d, 6H, *J* = 9.2 Hz, C**H_3_**-O), 3.82 (d, 6H, *J* = 9.6 Hz, C**H_3_**-O), 3.75–4.00 (m, 3H, CH*-NH + -O-C**H**_2_-CH_3_), 4.09–4.20 (m, 1H, CH*-NH), 4.25–4.50 (m, 3H, -O-C**H**_2_-CH_3 +_ N**H**-SO_2_), 4.86 (s, 1H, N**H**-SO_2_), 5.97 (d, 1H, *J* = 13.5 Hz, CH^*^-O), 5.98 (d, 1H, *J* = 14.3 Hz, CH^*^-O), 7.00–7.12 (m, 2H, **H**-Ar), 7.11–7.41 (m, 14H, **H**-Ar), 7.42–7.48 (m, 4H, **H**-Ar). ^13^C-NMR (100.62 MHz, CDCl_3_) δ: 13.98 (2CH_3_), 38.99 (2CH_2_), 54.27 (2C, d, *J*_C-P_ = 6.9 Hz, POCH_3_), 54.45 (2C, d, *J*_C-P_ = 6.9 Hz, POCH_3_), 57.74 (2CH), 61.76 (2OCH_2_), 71.14 (2C, d, *J*_C-P_ = 155.6 Hz, CH*-OP), 128.06 (4C, d, *J*_C-P_ = 6 Hz), 128.09 (4C), 128.57 (4C), 128.79 (4C), 129.51 (4C, d, *J*_C-P_ = 6 Hz), 132.2 (2C), 135.5 (2C), 150.69 (2C=O), 170.66 (2C=O). ^31^P-NMR (161.98 MHz, CDCl_3_) δ: 23.42. Anal. Calc. for C_21_H_27_N_2_O_9_PS: C 49.02, H 5.29, N 5.44, S 6.23. Found: C 45.07, H 6.04, N 5.81, S 6.72%. ESI-MS: (*m*/*z*) = 515.21 [M + H]^+^.

*(SR) and (SS)-Ethyl-2-((N-(((dimethoxyphosphoryl)(phenyl)methoxy)carbonyl)sulfamoyl) amino)-3-(1H-indol-3-yl) propanoate* (**3b**)*.* White powder, 84% yield; m.p. 116–118 °C; R*_f_* = 0.39 (CH_2_Cl_2_/MeOH, 90:10). IR (KBr, cm^−1^): 3274, 1747, 1471, 1371, 1250, 1164. ^1^H-NMR (400 MHz, CDCl_3_) δ: 0.99 (t, 3H, *J* = 7.20 Hz, C**H**_3_-CH_2-_O), 1.06 (t, 3H, *J* = 7.2 Hz, C**H**_3_-CH_2-_O), 3.11 (d, 4H, *J* = 6.0 Hz, C**H**_2_-CH*), 3.30–3.50 (m, 8H, 2C**H**_3_-O + 2CH*CO), 3.82–4.00 (m, 8H, 2C**H**_3_-O + OC**H**_2_), 4.21–4.26 (m, 2H, OC**H**_2_), 6.20 (d, 2H, *J* = 7.8 Hz, CH^*^-O), 6.68–6.98 (m, 6H, **H**-Ar), 7.18–7.26 (m, 8H, **H**-Ar), 7.37–7.40 (m, 6H, **H**-Ar), 9.60 (bs, 2H, N**H**-C=O). ^31^P-NMR (161.98 MHz, CDCl_3_) δ: 20.61. Anal. Calc. for C_23_H_28_N_3_O_9_PS: C 49.91, H 5.10, N 7.59, S 5.79. Found: C 49.97, H 5.04, N 7.68, S 5.83%. ESI-MS: (*m*/*z*) = 553.21 [M]^+^.

*(Dimethoxyphosphoryl)(phenyl)methyl ((2-oxooxazolidin-3-yl)sulfonyl)carbamate* (**1c**)*.* White powder; 92% yield; m.p. 123–125 °C; R*_f_* = 0.38 (CH_2_Cl_2_/MeOH, 90:10). IR (KBr, cm^−1^): 3255, 1748, 1663, 1357, 1254, 1118, 757, 629; ^1^H-NMR (400 MHz, CDCl_3_) δ: 3.40–3.43 (m, 2H, C**H**_2_-N), 3.61 (d, 3H, ^3^*J_H-P_* = 8.0 Hz, C**H**_3_-OP), 3.75 (d, 3H, ^3^*J_H-P_* = 8.0 Hz, CH_3_-OP), 4.60–4.63 (m, 2H, C**H**_2_-O), 6.04 (d, 1H, ^2^*J_H-P_* = 12.0 Hz, CH*-OP), 7.31–7.35 (m, 3H, **H**-Ar), 7.37-7.39 (m, 2H, **H**-Ar). ^13^C-NMR (100.62 MHz, CDCl_3_) δ: 46.58, 54.92 (d, *J*_C-P_ = 7 Hz, POCH_3_), 54.95 (d, *J*_C-P_ = 7 Hz, POCH_3_), 70.76, 71.02 (d, *J*_C-P_ = 171 Hz, CH*-OP), 127.93 (2C, d, *J*_C-P_ = 3 Hz), 128.75, 128.99 (2C, d, *J*_C-P_ = 2 Hz), 133.52, 155.06 (C=O), 155.12 (d, *J*_C-P_ = 12 Hz, C=O). ^31^P-NMR (161.98 MHz, CDCl_3_) δ: 18.93. Anal. Calc. for C_13_H_17_N_2_O_9_PS: C 38.24, H 4.20, N 6.86, S 7.85. Found: C 38.20, H 4.25, N 6.89, S 7.81%. ESI-MS: (*m*/*z*) = 431.5 [M + Na]^+^.

### 3.3. Determination of In Vitro Antibacterial Activity

The antimicrobial activity of the synthesized compounds was evaluated *in vitro* against Gram positive and Gram negative bacteria. Serial dilutions of the tested compounds in acetone were made in a concentration range from 0.5 to 512 µg/mL. All tests were performed in triplicate.

Firstly, compounds **1a**–**7a** and **1b** were screened for antibacterial activity by using the Kirby Bauer disc diffusion test on Mueller-Hinton agar plates. The medium was poured into Petri plates and allowed to solidify. These plates were inoculated with a bacterial inoculum prepared in physiologically sterile water with an OD of about 0.08. Sterilized disks of 6 mm (Schleicher and Schule, Germany) were each impregnated with 20 µL of different concentrations of the compounds and were deposited on the plates. The latter were then left at room temperature for 2 h and incubated at 37 °C for 24 h. The diameters of the inhibition zones (mm) were measured in accordance with the recommendations of the clinical and laboratory standards institute (CLSI 2017) [32]. For each bacterial strain, the best inhibition zone obtained was reported in Table 4.

Secondly, the MIC values were determined by the dilution broth method following the procedure recommended by the CLSI [32]. The serial dilutions of compounds, ranging from concentrations of 0.5 to 512 μg/mL, T were inoculated with fresh bacterial inoculums and then incubated at 37 °C for 24 h. The MIC value was considered as the lowest concentration showing visual inhibition of growth. Sulfamethoxazole-trimethoprime (Bio-Rad, Marseille, France) was used as the positive control (CMI = 25 µg/mL). Disks embedded with acetone were used as a negative one.

## 4. Conclusions

In summary, 12 new and original sulfamidocarbonyloxyphosphonates were synthesized and fully characterized by ^1^H, ^13^C, and ^31^P NMR spectroscopy, IR spectroscopy, and mass spectroscopy, as well as elemental analysis. The synthesized compounds **1a**–**7a** and **1b** were screened for *in vitro* evaluation as a proof of concept for designing new antibacterial agents containing both sulfamido and phosphonate moieties. Standard strains were chosen according to the screening protocol including Gram-positive and Gram-negative bacteria, which represent micro-organisms associated with important infections. All compounds showed promising *in vitro* antibacterial activity. Additionally, it has been demonstrated that our derivatives have more antibacterial effects on Gram-negative bacteria than Gram-positive ones except for compound **1b** (R_1_=CH_3_, R_2_=CH(*i*Bu)COOEt, R_3_=H). This latter is the only one active on both Gram-negative and Gram-positive bacteria. Compound **1a** (R_1_=CH_3_, R_2_=Bn, R_3_=H) had more pronounced activity against *P. aeruginosa*, whereas compound **4a** (R_1_=CH_3_, R_2_=3-F-C_6_H_4_, R_3_=H) had more activity on *E. coli*. Antibacterial effects will be investigated in further studies to explain the susceptibility of bacteria to our compounds. Further pharmacomodulation efforts are in progress to explore the impact of new substituents on the phenyl moiety and thereby will offer new expectations for sulfamidocarbonyloxyphosphonates as novel antibacterial agents.

## Figures and Tables

**Table 1:** The physical data and yields for sulfamidocarbonyloxyphosphonates **1a**–**8a** synthesized from primary and secondary amines.

Entry	-NR_2_R_3_	Target Molecule	Yield %	m.p. °C
**1a**	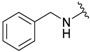	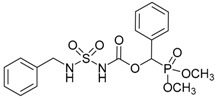	99	131–133
**2a**	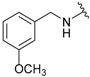	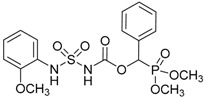	98	137–139
**3a**	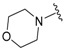	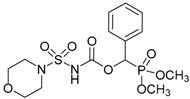	98	144–146
**4a**	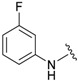	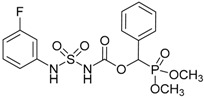	96	136–138
**5a**	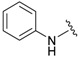	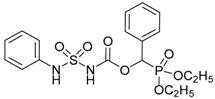	96	187–189
**6a**	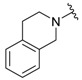	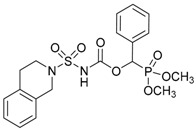	94	153–155
**7a**	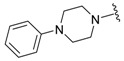	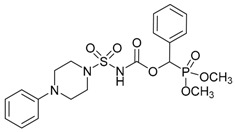	93	152–154
**8a**	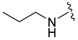	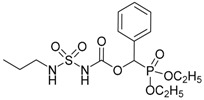	97	151–153

**Table 2:** Physical data and yields for sulfamidocarbonyloxyphosphonates **1b**–**3b** synthesized from amino acid esters.

Entry	R_4_	Target Molecule	Yield	m.p. °C
**1b**	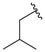	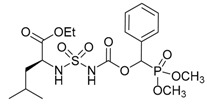	91	118–120
**2b**	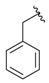	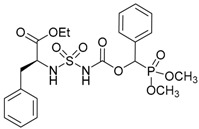	94	125–127
**3b**	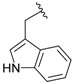	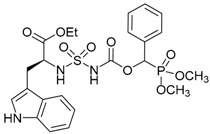	84	116–118

**Table 3:** Physical data and yield for the sulfamidocarbonyloxyphosphonate **1c** synthesized from oxazolidin-2-one.

Entry	Target Molecule	Yield %	m.p. °C
**1c**	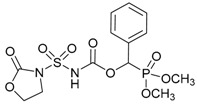	92	123–125

**Table 4:** Diameters of the inhibition zone (DIZ) of sulfamidocarbonyloxyphosphonate derivatives **1a**–**7a**, **1b**, and **SXT** toward Gram-positive and Gram-negative bacteria.

	Molecules	Diameters of Inhibition Zone (DIZ) in mm ^a^								
Bacterial Strains		1a	2a	3a	4a	5a	6a	7a	1b	SXT
***S. aureus* ATCC 25923**		15	16	14	12	15	12	13	13	22
***S. aureus* 1**		16	15	14	R ^b^	18	13	14	12	20
***S. aureus* 2**		16	15	16	R	16	15	12	13	18
***S. aureus* 3**		17	14	15	R	17	14	13	15	18
***E. coli* ATCC 25922**		24	23	25	25	23	21	23	18	20
***E. coli* 1**		17	22	18	20	20	20	19	18	18
***E. coli* 2**		22	19	24	24	22	18	23	20	18
***E. coli* 3**		20	22	23	22	19	18	20	17	20
***P. aeruginosa* ATCC 27853**		26	20	18	23	19	19	20	18	17
***P. aeruginosa* 1**		24	20	20	18	18	20	18	20	R
***P. aeruginosa* 2**		26	19	20	20	20	19	18	17	20
***P. aeruginosa* 3**		25	21	21	22	22	21	19	R	18
***K. pneumoniae* ATCC 700603**		22	19	13	20	19	20	21	19	22
***K. pneumoniae* 1**		22	21	18	25	15	18	19	15	R
***K. pneumoniae* 2**		20	21	17	25	20	18	16	R	17
***K. pneumoniae* 3**		20	18	18	24	19	19	22	21	R

^a^ All tests were performed in triplicate. ^b^ R: Resistant.

**Table 5:** Minimum inhibitory concentrations (MICs) of the sulfamidocarbonyloxyphosphonate derivatives **1a**–**7a** and **1b** toward Gram-positive and Gram-negative bacteria.

	Molecules	MIC (µg/mL) ^a^							
Bacterial Strains		1a	2a	3a	4a	5a	6a	7a	1b
***S. aureus* ATCC 25923**		128	128	256	512	256	128	256	15
***S. aureus* 1**		128	256	256	R ^b^	64	128	256	15
***S. aureus* 2**		64	128	128	R	128	64	128	18
***S. aureus* 3**		64	128	128	R	128	128	128	15
***E. coli* ATCC 25922**		1	2	2	0.5	2	2	8	18
***E. coli* 1**		32	16	16	4	8	16	4	18
***E. coli* 2**		4	16	2	0.5	4	16	8	20
***E. coli* 3**		8	32	4	1	16	4	32	17
***P. aeruginosa* ATCC 27853**		0.5	1	4	2	2	1	4	18
***P. aeruginosa* 1**		0.5	2	2	4	2	2	2	20
***P. aeruginosa* 2**		1	2	2	4	4	2	8	17
***P. aeruginosa* 3**		0.5	6	1	2	2	4	4	R
***K. pneumoniae* ATCC 700603**		4	32	256	16	128	128	32	19
***K. pneumoniae* 1**		4	64	32	2	128	64	128	15
***K. pneumoniae* 2**		8	16	16	2	32	128	128	R
***K. pneumoniae* 3**		8	16	32	4	128	64	64	21

^a^ All tests were performed in triplicate and STX was used as the positive control (MIC = 25 µg/mL). ^b^ R: Resistant.

## Supplementary Materials

Supplementary materials are available on line.
