# Supplementary material for: Synthesis, Spectroscopic Characterization, and In Vitro Antibacterial Evaluation of Novel Functionalized Sulfamidocarbonyloxyphosphonates
Source: Molecules. 2018 Jul 10;23(7):1682. doi: 10.3390/molecules23071682 (PMC6099799; doi:10.3390/molecules23071682)
Supplement: Supplementary file 1 [file molecules-23-01682-s001.pdf]

Supporting information

Synthesis, spectroscopic characterization and *in vitro* antibacterial evaluation of novel functionalized sulfamidocarbonyloxyphosphonates

Abdeslem Bouzina [1], Khaoula Bechlem [1], Hajira Berredjem [2], Billel Belhani [1], Imène Becheker [2], Jacques Lebreton [3], Marc Le Borgne [4], Zouhair Bouaziz [4], Christelle Marminon [4,*] and Malika Berredjem [1,*]

[1] Laboratory of Applied Organic Chemistry, Synthesis of Biomolecules and Molecular Modelling Group, Badji-Mokhtar - Annaba University, Box 12, 23000 Annaba, Algeria; bouzinaabdeslem@yahoo.fr (A.B.); bechlemkhaoula92@yahoo.com (K.B); billelbelhani@yahoo.com (B.B.); malika.berredjem@univ-annaba.org (M.B.)

[2] Laboratory of Applied Biochemistry and Microbiology, Department of Biochemistry, Badji-Mokhtar University, Annaba, Algeria; hajira.berredjem@univ-annaba.dz (H.B.); i_mene7@msn.com (I.B.)

[3] CNRS, Université de Nantes, Chimie et Interdisciplinarité : Synthèse, Analyse, Modélisation (CEISAM), UMR CNRS 6230, 2 rue de la Houssinière, BP92208, 44322 Nantes Cedex 3, France; jacques.lebreton@univ-nantes.fr (J.L.)

[4] Université de Lyon, Université Lyon 1, Faculté de Pharmacie - ISPB, EA 4446 Bioactive Molecules and Medicinal Chemistry, SFR Santé Lyon-Est CNRS UMS3453 - INSERM US7, 69373 Lyon cedex 8, France; marc.le-borgne@univ-lyon1.fr (M.L.B.); zouhair.bouaziz@univ-lyon1.fr (Z.B.); christelle.marminon-davoust@univ-lyon1.fr (C.M.D.)

* Correspondence: christelle.marminon-davoust@univ-lyon1.fr (C.M.D.); malika.berredjem@univ-annaba.org (M.B.); Tel.: +33478772859 (C.M.D.); +21338876567 (M.B.)

1. General

All chemicals and solvents were purchased from common commercial sources and were used as received without any further purification. All reactions were monitored by TLC on silica Merck 60 F254 percolated aluminum plates and were developed by spraying with ninhydrin solution. Column chromatography was performed with Merck silica gel (230-400 mesh). The Proton nuclear magnetic resonance (^{1}H NMR) spectra were recorded on Bruker or Jeol spectrometers at 400 MHz. Chemical shifts are reported in δ units (ppm) with TMS as reference (δ 0.00). All coupling constants (*J*) are reported in Hertz. Multiplicity is indicated by one or more of the following: b (broad), s (singlet), d (doublet), t (triplet), q (quartet), dd (doublet of doublet), m (multiplet). The Carbon nuclear magnetic resonance (^{13}C NMR) spectra were recorded on Bruker or Jeol spectrometers at 100.62 MHz. Chemical shifts are reported in δ units (ppm) and coupling constants (*J*) in Hertz. The Phosphorus (^{31}P NMR) and Fluor (^{19}F NMR) nuclear magnetic resonance spectra were recorded on a Bruker spectrometer respectively at 161.98 MHz and 316.48 MHz. The Infrared spectra (IR) were recorded on a Perkin Elmer 600 spectrometer. The Mass spectra were recorded on a shimadzu QP 1100 Ex mass spectrometer operating at an ionization potential of 70 eV. Elemental analysis was recorded on a EURO E.A. 3700 apparatus. All melting points were determined on a Büchi B-545 apparatus in open capillary tubes.

Ultrasound assisted reactions were carried out using a FUNGILAB ultrasonic bath with a frequency of 40 kHz and a nominal power of 250 W. The reactions were carried out in an open glass tube (diameter: 25 mm; thickness: 1 mm; volume: 20 mL) at room temperature.

2.a. Typical experimental procedure for the synthesis of α-hydroxyphosphonates

A mixture of aldehyde (1.0 mmol) and trimethylphosphite (1.2 mmol), in a 20 ml open glass tube at room temperature, was subjected to the ultrasonic irradiation for appropriate time. After completion of the reaction, as indicated by TLC, dichloromethane/methanol (9:1), a mixture of diethyl ether/*n*-hexane (6:4) was added to the reaction mixture and the pure product was crystallized at 6 °C overnight. The product was finally filtered and dried.

2.b. Typical experimental procedure for the synthesis of carboxylsulfamidophosphonates

A solution of α-hydroxyphosphonate (1.1 equiv) in anhydrous CH_2Cl_2 (5 ml) was added dropwise to a stirring solution of chlorosulfonyl isocyanate (CSI) (1 equiv) in anhydrous CH_2Cl_2 (5 ml) at 0 ^{o}C over a period of 20 min. The resulting solution was transferred to a mixture of primary or secondary amine (1.1 equiv) or amino acid ester or oxazolidinone in CH_2Cl_2 (10 ml) and triethylamine (1.1-1.5 equiv). The solution was stirred at 0 ^{o}C for less than 1-2 h. Then the reaction mixture was neutralized adding a solution of HCl 0.1 N to pH 7. The organic layer was extracted, washed with water, then dried over anhydrous sodium sulfate, filtered and concentrated *in vacuo*. A mixture of diethyl ether/*n*-hexane (6:4) was added to the reaction and the pure product crystallized at 6° C overnight. The product was finally filtered and dried in excellent yields.

3. Spectral data:

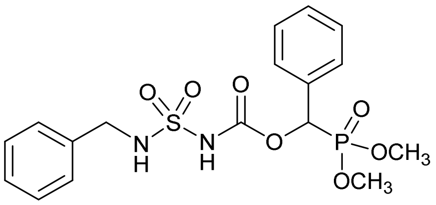

(Dimethoxyphosphoryl)(phenyl)methyl (*N*-benzylsulfamoyl)carbamate (Table 1, Entry 1a):

White powder, 99% yield, m.p. 131-133 °C, R_f = 0.43 (CH_2Cl_2/MeOH, 90:10). IR (KBr, cm^{-1}): 3365, 3298, 1733, 1481, 1364, 1249, 1170. ^{1}H-NMR (400 MHz, $CDCl_3$) δ: 3.57 (d, 3H, $^3J_{H-P}$ = 10.4 Hz, C**H**$_3$-OP), 3.77 (d, 3H, $^3J_{H-P}$ = 10.8 Hz, C**H**$_3$-OP), 4.11 (dd, 1H, J_1 = 13.6 Hz, J_2 = 5.4 Hz, C**H**-N), 4.23 (dd, 1H, J_1 = 14.0 Hz, J_2 = 5.6 Hz, C**H**-N), 5.61 (bs, 1H, N**H**-SO_2), 6.00 (d, 1H, $^2J_{H-P}$ = 12 Hz, C**H***-OP), 7.18-7.28 (m, 5H, **H**-Ar), 7.36-7.42 (m, 3H, **H**-Ar), 7.47-7.53 (m, 2H, **H**-Ar), 8.90 (bs, 1H, N**H**-C=O). ^{13}C-NMR (100.62 MHz, $CDCl_3$) δ: 48.15 (CH_2), 54.19 (d, J_{C-P} = 7 Hz, $POCH_3$), 54.48 (d, J_{C-P} = 7 Hz, $POCH_3$), 72.52 (d, J_{C-P} = 172 Hz, CH*-OP), 128.03 (2C, d, J_{C-P} = 6 Hz), 128.18 (2C), 128.34 (2C), 128.54, 128.96 (2C, d, J_{C-P} = 4 Hz), 129.58, 132.41, 135.54, 150.49 (d, J_{C-P} = 11 Hz, C=O). ^{31}P-NMR (161.98 MHz, $CDCl_3$) δ: 19.10. Anal. Calc. for $C_{17}H_{21}N_2O_7PS$: C 47.66, H 4.94, N 6.54, S 7.48. Found: C 47.71, H 4.89, N 6.52, S 7.44%. ESI-MS: (m/z) = 429.1 $[M+H]^+$.

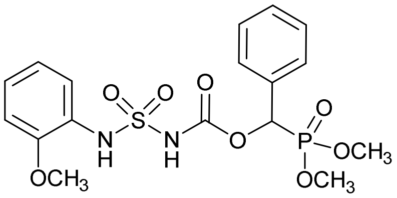

(Dimethoxyphosphoryl)(phenyl)methyl *N*-(2-methoxyphenyl)sulfamoylcarbamate (Table 1, Entry 2a):

White powder, 98% yield, m.p. 137-139 °C, R_f = 0.40 (CH_2Cl_2/MeOH, 90:10). IR(KBr, cm^{-1}): 3342, 3275, 1733, 1489, 1361, 1252, 1136. ^{1}H-NMR (400 MHz, $CDCl_3$) δ: 3.50 (d, 3H, $^3J_{H-P}$= 10.8 Hz, C**H**$_3$-OP), 3.54 (s, 3H, C**H**$_3$-O), 3.62 (d, 3H, $^3J_{H-P}$ = 10.8 Hz, C**H**$_3$-OP), 5.94 (d, 1H, $^2J_{H-P}$ = 14.0 Hz, C**H***-OP), 6.75 (dd, 1H, J_1 = 8.0 Hz, J_2 = 1.2 Hz, **H**$_{ortho}$-Ar OMe), 6.84 (td, 1H, J_1 = 7.6 Hz, J_2 = 1.2 Hz, **H**$_{metha}$-Ar), 7.07 (td, 1H, J_1 = 6.8 Hz, J_2 = 1.2 Hz, **H**-Ar), 7.31-7.39 (m, 5H, **H**-Ar), 7.43 (dd, 1H, J_1 = 8.0 Hz, J_2 = 1.6 Hz, **H**$_{ortho}$-Ar NH), 7.55 (bs, 1H, N**H**-SO_2), 9.85 (bs, 1H, N**H**-C=O). ^{13}C-NMR (100.62 MHz, $CDCl_3$) δ: 54.16 (d, J_{C-P} = 7 Hz, $POCH_3$), 54.26 (d, J_{C-P} = 7 Hz, $POCH_3$), 55.79 (OCH_3), 72.20 (d, J_{C-P} = 174 Hz, CH*-OP), 111.09, 120.87, 121.04, 121.37, 125.95, 128.09 (2C, d, J_{C-P} = 6 Hz), 128.86, 129.33 (2C, d, J_{C-P} = 3 Hz), 132.52, 149.73, 150.09 (d, J_{C-P} = 12 Hz, C=O). ^{31}P-NMR (161.98 $CDCl_3$) δ: 18.81. Anal. Calc. for $C_{17}H_{21}N_2O_8PS$: C 45.95, H 4.76, N 6.30, S 7.22. Found: C 45.90, H 4.81, N 6.28, S 7.26%. ESI-MS: (m/z) = 445.1 $[M+H]^+$.

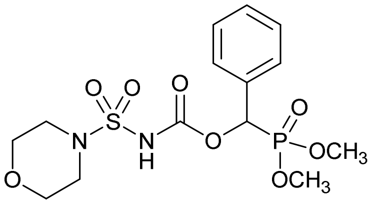

(Dimethoxyphosphoryl)(phenyl)methyl morpholinosulfonylcarbamate (Table 1, Entry 3a):

White powder, 98% yield, m.p. 144-146 °C, R_f = 0.47 (CH_2Cl_2/MeOH, 90:10). IR (KBr, cm^{-1}): 3447, 3297, 1732, 1481, 1361, 1247, 1185, 769, 687. ^{1}H-NMR (400 MHz, $CDCl_3$) δ: 3.29-3.31 (m, 4H, 2 C**H**$_2$-N), 3.56 (d, 3H, $^3J_{H-P}$ = 10.4 Hz, C**H**$_3$-OP), 3.65-3.67 (m, 4H, 2 C**H**$_2$-O), 3.84 (d, 3H,$^3J_{H-P}$ = 10.8 Hz, C**H**$_3$-OP), 6.02 (d, 1H, $^2J_{H-P}$ = 13.6 Hz, C**H***-OP), 7.37-7.40 (m, 3H, **H**-Ar), 7.51-7.55 (m, 2H, **H**-Ar) 9.92 (bs, 1H, N**H**-C=O). ^{13}C-NMR (100.62 MHz, $CDCl_3$) δ: 46.70 (2C, CH_2-N), 54.23 (d, J_{C-P} = 7 Hz, $POCH_3$), 54.38 (d, J_{C-P} = 7 Hz, $POCH_3$), 66.32 (2C, CH_2-O), 72.09 (d, J_{C-P} = 174 Hz, CH*-OP), 128.16 (2C, d, J_{C-P} = 6 Hz), 128.92 (2C, d, J_{C-P} = 1 Hz), 129.56 (d, J_{C-P} = 3 Hz), 132.46, 150.72 (d, J_{C-P} = 12 Hz, C=O). ^{31}P-NMR (161.98 $CDCl_3$) δ: 18.93. Anal. Calc. for $C_{14}H_{21}N_2O_8PS$: C 41.18, H 5.18, N 6.86, S 7.85. Found: C 41.22, H 5.23, N 6.83, S 7.81%. ESI-MS: (m/z) = 409.1 $[M+H]^+$.

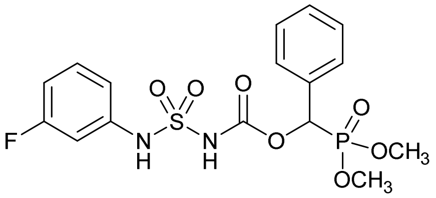

(Dimethoxyphosphoryl)(phenyl)methyl *N*-(3-fluorophenyl)sulfamoylcarbamate (Table 1, Entry 4a):
White powder, 96% yield, m.p. 136-138 °C, R_f = 0.41 (CH_2Cl_2/MeOH, 90:10). IR (KBr, cm^{-1}): 3311, 3297, 1758, 1477, 1355, 1251, 1166. ^{1}H-NMR (400 MHz, $CDCl_3$) δ: 3.62 (dd, 3H, J_1 = 38.8 Hz, J_2 = 10.4 Hz, C**H**$_3$-O), 3.72 (dd, 3H, J_1 = 10.4 Hz, J_2 = 1.2 Hz, CH_3-O), 5.95 (d, 1H, J = 13.6, C**H***-O), 6.88-7.04 (m, 3H, **H**-Ar), 7.19-7.41 (m, 6H, **H**-Ar). ^{13}C-NMR (100.62 MHz, $CDCl_3$) δ: 54.85, 54.90, 71.86, 73.21, 128.14, 128.30, 129.23, 129.65, 12.91, 131.15, 131.75, 134.19, 134.56, 138.25, 138.45, 150.36. ^{31}P-NMR (161.98 $CDCl_3$) 20.61. ^{19}F-NMR (316.48 MHz, $CDCl_3$) δ: -111.62. Anal. Calc. for $C_{16}H_{18}FN_2O_7PS$: C 44.45, H 4.20, N 6.48, S 7.42. Found: C 44.40, H 4.23, N 6.52, S 7.41%. ESI-MS: (m/z) = 433.1 $[M+H]^+$.

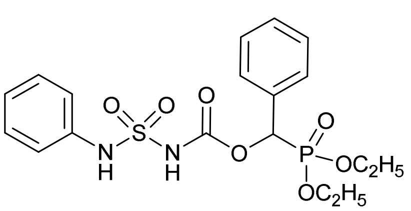

(Diethoxyphosphoryl)(phenyl)methyl *N*-phenylsulfamoylcarbamate (Table 1, Entry 5a):
White powder, 96% yield, m.p. 187-189 °C, R_f = 0.42 (CH_2Cl_2/MeOH, 90:10). IR (KBr, cm^{-1}): 3447, 3297, 1733, 1481, 1384, 1247, 1185. ^{1}H-NMR (400 MHz, $CDCl_3$) δ: 1.03 (t, 3H, J = 7.0 Hz, C**H**$_3$), 1.30 (t, 3H, J = 7.0 Hz, C**H**$_3$), 3.59-3.68 (m, 1H, C**H**$_2$-O), 3.82-3.90 (m, 1H, C**H**$_2$-O), 4.07-4.17 (m, 2H, C**H**$_2$-O), 5.82 (d, 1H, J = 8.8 Hz, C**H***OP), 6.47 (s, 1H, N**H**-SO_2), 6.80 (dd, 2H, J_1 = 8.8 Hz, J_2 = 1.2 Hz, **H**-Ar), 7.02 (t, 1H, J = 7.6 Hz, **H**-Ar), 7.15 (t, 2H, J = 7.6 Hz, **H**-Ar), 7.20-7.26 (m, 5H, **H**-Ar).^{13}C-NMR (100.62 MHz, $CDCl_3$) δ: 16.32 (CH_3), 16.59 (CH_3), 63.96 (CH_2), 64.10 (CH_2), 72.46 (d, J_{C-P} = 170 Hz, CH*-OP), 119.77 (2C), 124.46, 128.31 (2C, d, J_{C-P} = 6 Hz), 128.76 (2C), 128.90 (2C), 129.32, 134.25, 136.86, 150.40 (d, J_{C-P} = 16 Hz, C=O). ^{31}P-NMR (161.98 MHz, $CDCl_3$) δ: 19.61. Anal. Calc. for $C_{18}H_{23}N_2O_7PS$: C 48.87, H 5.24, N 6.33, S 7.25. Found: C 48.93, H 5.21, N 6.28, S 7.26%. ESI-MS: (m/z) = 443.1 $[M+H]^+$.

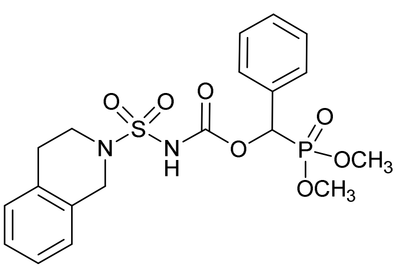

(Dimethoxyphosphoryl)(phenyl)methyl (3,4-dihydroisoquinolin-2(1*H*)-yl)sulfonylcarbamate (Table 1, Entry 6a):

Color powder, 94% yield, m.p. 153-155 °C, R_f = 0.49 (CH_2Cl_2/MeOH, 90:10). IR (KBr, cm^{-1}): 3258, 1750, 1360, 1454, 1234, 1120. ^{1}H-NMR (400 MHz, $CDCl_3$) δ: 2.87 (t, 2H, *J* = 6.0 Hz, C_{Ar}-**CH$_2$**-CH_2), 3.55 (d, 3H, $^3J_{H-P}$ = 10.0 Hz, C**H**$_3$-OP), 3.60 (t, 2H, *J* = 6.0 Hz, CH_2-**CH$_2$**-N), 3.75 (d, 3H, $^3J_{H-P}$ = 10.0 Hz, C**H**$_3$-O), 4.52 (s, 2H, C_{Ar}-**CH$_2$**-N), 6.00 (d, 1H, $^2J_{H-P}$ = 12.0 Hz, C**H***-O), 7.01-7.08 (m, 2H, **H**-Ar), 7.14-7.16 (m, 2H, **H**-Ar), 7.33-7.36 (m, 3H, **H**-Ar), 7.48-7.51 (m, 2H, **H**-Ar), 9.91 (s, 1H, N**H**-C=O). ^{13}C-NMR (100.62 MHz, $CDCl_3$) δ: 28.38 (CH_2), 44.47 (NCH_2), 47.78 (NCH_2), 54.63 (2C, $POCH_3$), 72.04 (d, J_{C-P} = 178 Hz, CH*-OP), 126.4, 126.6, 127.1, 128.6 (2C), 128.8, 129.60 (2C), 129.80, 131.41, 132.4, 133.2, 150.95 (d, J_{C-P} = 16 Hz, C=O). ^{31}P-NMR (161.98 MHz, $CDCl_3$) δ: 18.76. Anal. Calc. for $C_{19}H_{23}N_2O_7PS$: C 50.22, H 5.10, N 6.10, S 7.06. Found: C 50.19, H 5.15, N 6.16, S 7.10%. ESI-MS: (m/z) = 453.2 $[M-H]^+$.

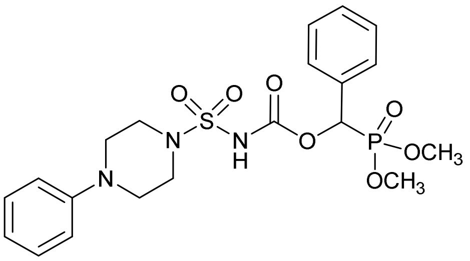

(Dimethoxyphosphoryl)(phenyl)methyl(4-phenylpiperazin-1-yl)sulfonylcarbamate (Table 1a, Entry 7a):

White powder, 93% yield, m.p. 152-154 °C, R_f = 0.50 (CH_2Cl_2/MeOH, 90:10). IR (KBr, cm^{-1}): 3337, 1741, 1449, 1360, 1248, 1167. ^{1}H-NMR (400 MHz, $CDCl_3$) δ: 3.10-3.40 (m, 4H, 2 **CH$_2$**- N-SO_2), 3.42-3.62 (m, 4H, 2 **CH$_2$**-N-C_{Ar}), 3.67 (d, 3H, *J* = 10.6 Hz, C**H$_3$**-O), 3.75 (d, 3H, *J* = 10.8 Hz, C**H$_3$**-O), 6.05 (d, 1H, $^2J_{H-P}$ = 14.0 Hz, C**H***-O), 6.80-6.96 (m, 3H, **H**-Ar), 7.25-7.40 (m, 5H, **H**-Ar), 7.45-7.56 (m, 2H, **H**-Ar). ^{13}C-NMR (100.62 MHz, $CDCl_3$) δ: 46.78 (2C, NCH_2), 48.90 (2C, NCH_2), 54.61 (d, J_{C-P} = 7 Hz, $POCH_3$), 54.62 (d, J_{C-P} = 7 Hz, $POCH_3$), 70.86 (d, J_{C-P} = 142.4 Hz, CH*-OP), 117.37 (2C), 120.93, 127.87 (2C, d, J_{C-P} = 5 Hz), 128.57, 128.92 (2C), 129.65 (2C), 132.49, 136.81, 151.27 (d, J_{C-P} = 12 Hz, C=O). ^{31}P-NMR (161.98 MHz, $CDCl_3$) δ: 19.82. Anal. Calc. for $C_{20}H_{26}N_3O_7PS$: C 49.68, H 5.42, N 8.69, S 6.63. Found: C 49.73, H 5.46, N 8.65, S 6.67%. ESI-MS: (m/z) = 482.3 $[M-H]^+$.

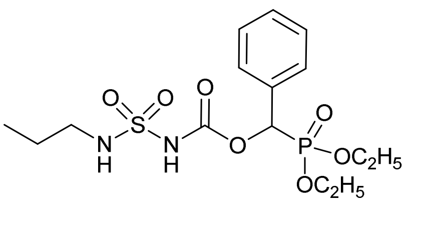

(Diethoxyphosphoryl)(phenyl)methyl *N*-propylsulfamoylcarbamate (Table 1, Entry 8a):

White powder, 97% yield, m.p. 151-153 °C, R_f = 0.43 (CH_2Cl_2/MeOH, 90:10). IR (KBr, cm^{-1}): 3369, 3061, 1758, 1475, 1355, 1240, 1156, 763, 697. ^{1}H-NMR (400 MHz, $CDCl_3$) δ: 0.72 (t, 3H, *J* = 8.8 Hz, **C**$\mathbf{H_3}$-Pr), 1.09 (t, 3H, *J* = 9.4 Hz, **C**$\mathbf{H_3}$-OEt), 1.12-1.27 (m, 2H, **C**$\mathbf{H_2}$-Pr), 1.37 (t, 3H, *J* = 9.4 Hz, **C**$\mathbf{H_3}$-OEt), 2.48-2.59 (m, 1H, **C**$\mathbf{H_2}$-N), 2.78-2.87 (m, 1H, **C**$\mathbf{H_2}$-N), 3.67-4.05 (m, 2H, $\mathbf{CH_2}$-OP), 4.25 (1H, m, NH), 4.74 (dq, 2H, $^3J_{H-P}$ = 11.8 Hz, $^3J_{H-H}$ = 7.5 Hz, **C**$\mathbf{H_2}$-OP), 6.00 (dd, 1H, $^2J_{H-P}$ = 11.3 Hz, *J* = 8.8 Hz, C**H***-O), 7.35-7.38 (m, 3H, **H**-Ar), 7.50-7.52 (m, 2H, **H**-Ar). Anal. Calc. for $C_{15}H_{25}N_2O_7PS$: C 44.11, H 6.17, N 6.86, S 7.85. Found: C 44.29, H 6.79, N 6.91, S 7.80%. ESI-MS: (m/z) = 409.2 $[M+H]^+$.

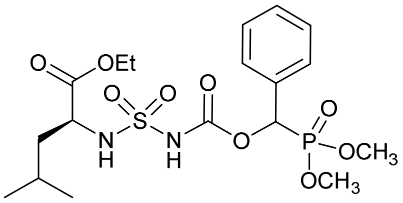

(SR) and (SS)-Ethyl-2-((*N*-(((dimethoxyphosphoryl)(phenyl)methoxy)carbonyl)sulfamoyl)amino)-4-methylpentanoate (Table 2, Entry 1b):

White powder, 91% yield; m.p. 118-120 °C, R_f = 0.39 (CH_2Cl_2/MeOH, 90:10). IR (KBr, cm^{-1}): 3274, 1747 (l), 1470, 1371, 1251, 1164. ^{1}H-NMR (400 MHz, $CDCl_3$) δ: 0.81-0.87 (m, 12H, **C**$\mathbf{H_3}$-CH_{isop}), 1.05-1.35 (m, 6H, O-$\mathbf{CH_2}$-$\mathbf{CH_3}$), 1.36-1.60 (m, 4H, 2**C**$\mathbf{H}_{isop}$+ 1**C**$\mathbf{H_2}$-CH_{isop}), 1.20 (m, 2H, 1**C**$\mathbf{H_2}$-CH_{isop}), 3.51 (d, 3H, *J* = 10.6 Hz, **C**$\mathbf{H_3}$-O), 3.52 (d, 3H, *J* = 10.6 Hz, **C**$\mathbf{H_3}$-O), 3.60-3.75 (m, 1H, **CH***-NH), 3.75-3.99 (m, 3H, -O-$\mathbf{CH_2}$-CH_3 + **CH*-**NH), 3.78 (d, 6H, *J* = 10.80 Hz, **C**$\mathbf{H_3}$-O), 4.00-4.25 (m, 2H, -O-$\mathbf{CH_2}$-CH_3), 5.79 (bs, 1H, N**H**-SO_2), 5.96 (d, 1H, *J* = 13.9 Hz, C**H***-O), 6.00 (d, 1H, *J* = 14.3 Hz, C**H***-O), 6.21 (bs, 1H, N**H**-SO_2), 7.32-7.40 (m, 6H, **H**-Ar), 7.50-7.56 (m, 4H, **H**-Ar), 9.86 (bs, 1H, N**H**-C=O). ^{13}C-NMR (100.62 MHz, $CDCl_3$) δ: 14.04 (2CH_3), 22.76 (4C), 24.37 (2C), 41.95(2C), 54.19 (2$POCH_3$), 54.44 (2$POCH_3$), 55.55 (2C), 61.63 (2OCH_2), 72.13 (2C, d, J_{C-P} = 142.4 Hz, CH*-OP), 128.03 (4C), 128.12 (2C), 128.75 (4C, d, J_{C-P} = 5 Hz), 132.50 (2C), 150.60 (2C, d, J_{C-P} = 2 Hz, C=O), 172.01 (2C=O). ^{31}P-NMR (161.98 MHz, $CDCl_3$) δ: 21.61. Anal. Calc. for $C_{18}H_{29}N_2O_9PS$: C 45.00, H 6.08, N 5.83, S 6.67. Found: C 45.07, H 6.04, N 5.81, S 6.72%. ESI-MS: (m/z) = 481.1 $[M+H]^+$.

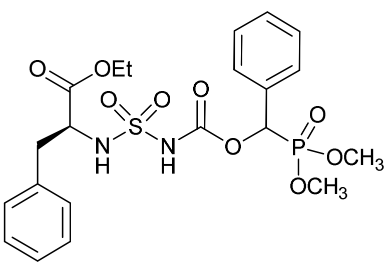

(SR) and (SS)-Ethyl-2-((*N*-(((dimethoxyphosphoryl)(phenyl)methoxy)carbonyl)sulfamoyl)amino)-3-phenylpropanoate (Table 2, Entry 2b):

White powder, 94% yield; m.p. 125-127 °C, R_f = 0.41 (CH_2Cl_2/MeOH, 90:10). IR (KBr, cm^{-1}): 3279, 1744 (l), 1455, 1373, 1249, 1162. ^{1}H-NMR (400 MHz, $CDCl_3$) δ: 1.01 (t, 3H, *J* = 7.6 Hz, **C**$\mathbf{H_3}$-CH_2-O), 1.02 (t, 3H, *J* = 7.6 Hz, **C**$\mathbf{H_3}$-CH_2-O), 2.85-3.15 (m, 4H, **C**$\mathbf{H_2}$-Ar), 3.49 (d, 6H, *J* = 9.2 Hz, $\mathbf{CH_3}$-O), 3.82 (d, 6H, *J* = 9.6 Hz, $\mathbf{CH_3}$-O), 3.75-4.00 (m, 3H, **CH***-NH + -O-**C**$\mathbf{H_2}$-CH_3), 4.09-4.20 (m, 1H, **CH***-NH), 4.25-4.50 (m, 3H, -O-$\mathbf{CH_2}$-CH_3 + N**H**-SO_2), 4.86 (s, 1H, N**H**-SO_2), 5.97 (d, 1H, *J* = 13.5 Hz, C**H***-O), 5.98 (d, 1H, *J* = 14.3 Hz, C**H***-O), 7.00-7.12 (m, 2H, **H**-Ar), 7.11-

7.41 (m, 14H, **H**-Ar), 7.42-7.48 (m, 4H, **H**-Ar). ^{13}C-NMR (100.62 MHz, $CDCl_3$) δ: 13.98 (2CH_3), 38.99 (2CH_2), 54.27 (2C, d, J_{C-P} = 6.9 Hz, $POCH_3$), 54.45 (2C, d, J_{C-P} = 6.9 Hz, $POCH_3$), 57.74 (2CH), 61.76 (2OCH_2), 71.14 (2C, d, J_{C-P} = 155.6 Hz, CH*-OP), 128.06 (4C, d, J_{C-P} = 6 Hz), 128.09 (4C), 128.57 (4C), 128.79 (4C), 129.51 (4C, d, J_{C-P} = 6 Hz), 132.2 (2C), 135.5 (2C), 150.69 (2C=O), 170.66 (2C=O). ^{31}P-NMR (161.98 MHz, $CDCl_3$) δ: 23.42. Anal. Calc. for $C_{21}H_{27}N_2O_9PS$: C 49.02, H 5.29, N 5.44, S 6.23. Found: C 45.07, H 6.04, N 5.81, S 6.72%. ESI-MS: (m/z) = 515.21 $[M+H]^+$.

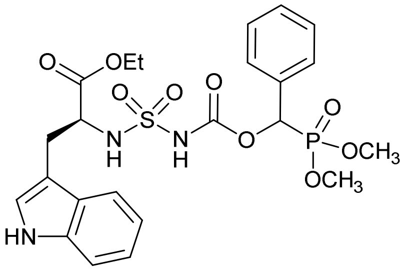

(SR) and (SS)-Ethyl 2-((*N*-(((dimethoxyphosphoryl)(phenyl)methoxy)carbonyl)sulfamoyl)amino)-3-(1*H*-indol-3-yl)propanoate (Table 2, Entry 3b):

White powder, 84% yield; m.p. 116-118 °C; R_f = 0.39 (CH_2Cl_2/MeOH, 90:10). IR (KBr, cm^{-1}): 3274, 1747, 1471, 1371, 1250, 1164. ^{1}H-NMR (400 MHz, $CDCl_3$) δ: 0.99 (t, 3H, *J* = 7.2 Hz, C**H**$_3$-CH$_2$-O), 1.06 (t, 3H, *J* = 7.2 Hz, C**H**$_3$-CH$_2$-O), 3.11 (d, 4H, *J* = 6.0 Hz, C**H**$_2$-CH*), 3.30-3.50 (m, 8H, 2C**H**$_3$-O + 2C**H***CO), 3.82-4.00 (m, 8H, 2C**H**$_3$-O + OC**H**$_2$), 4.21-4.26 (m, 2H, OC**H**$_2$), 6.20 (d, 2H, *J* = 7.80 Hz, C**H***-O), 6.68-6.98 (m, 6H, **H**-Ar), 7.18-7.26 (m, 8H, **H**-Ar), 7.37-7.40 (m, 6H, **H**-Ar), 9.60 (bs, 2H, N**H**-C=O). ^{31}P-NMR(161.98 MHz, $CDCl_3$) δ: 20.61. Anal. Calc. for $C_{23}H_{28}N_3O_9PS$: C 49.91, H 5.10, N 7.59, S 5.79. Found: C 49.97, H 5.04, N 7.68, S 5.83%. ESI-MS: (m/z) = 553.21 $[M]^+$.

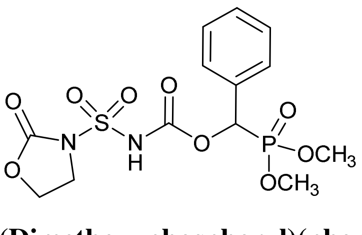

(Dimethoxyphosphoryl)(phenyl)methyl (2-oxooxazolidin-3-yl)sulfonylcarbamate (Table 3, Entry 1c):

White powder; 92% yield; m.p. 123-125 °C; R_f = 0.38 (CH_2Cl_2/MeOH, 90:10). IR (KBr, cm^{-1}): 3255, 1748, 1663, 1357, 1254, 1118, 757, 629; ^{1}H-NMR (400 MHz, $CDCl_3$) δ: 3.40-3.43 (m, 2H, C**H**$_2$-N), 3.61 (d, 3H, $^3J_{H-P}$ = 8.0 Hz, C**H**$_3$-OP), 3.75 (d, 3H, $^3J_{H-P}$ = 8.0 Hz, CH_3-OP), 4.60-4.63 (m, 2H, C**H**$_2$-O), 6.04 (d, 1H, $^2J_{H-P}$ = 12.0 Hz, C**H***-OP), 7.31-7.35 (m, 3H, **H**-Ar), 7.37-7.39 (m, 2H, **H**-Ar). ^{13}C-NMR (100.62 MHz, $CDCl_3$) δ: 46.58, 54.92 (d, J_{C-P} = 7 Hz, $POCH_3$), 54.95 (d, J_{C-P} = 7 Hz, $POCH_3$), 70.76, 71.02 (d, J_{C-P} = 171 Hz, CH*-OP), 127.93 (2C, d, J_{C-P} = 3 Hz), 128.75, 128.99 (2C, d, J_{C-P} = 2 Hz), 133.52, 155.06 (C=O), 155.12 (d, J_{C-P} = 12 Hz, C=O). ^{31}P-NMR (161.98 MHz, $CDCl_3$) δ: 18.93. Anal. Calc. for $C_{13}H_{17}N_2O_9PS$: C 38.24, H 4.20, N 6.86, S 7.85. Found: C 38.20, H 4.25, N 6.89, S 7.81%. ESI-MS: (m/z) = 431.5 $[M+Na]^+$.

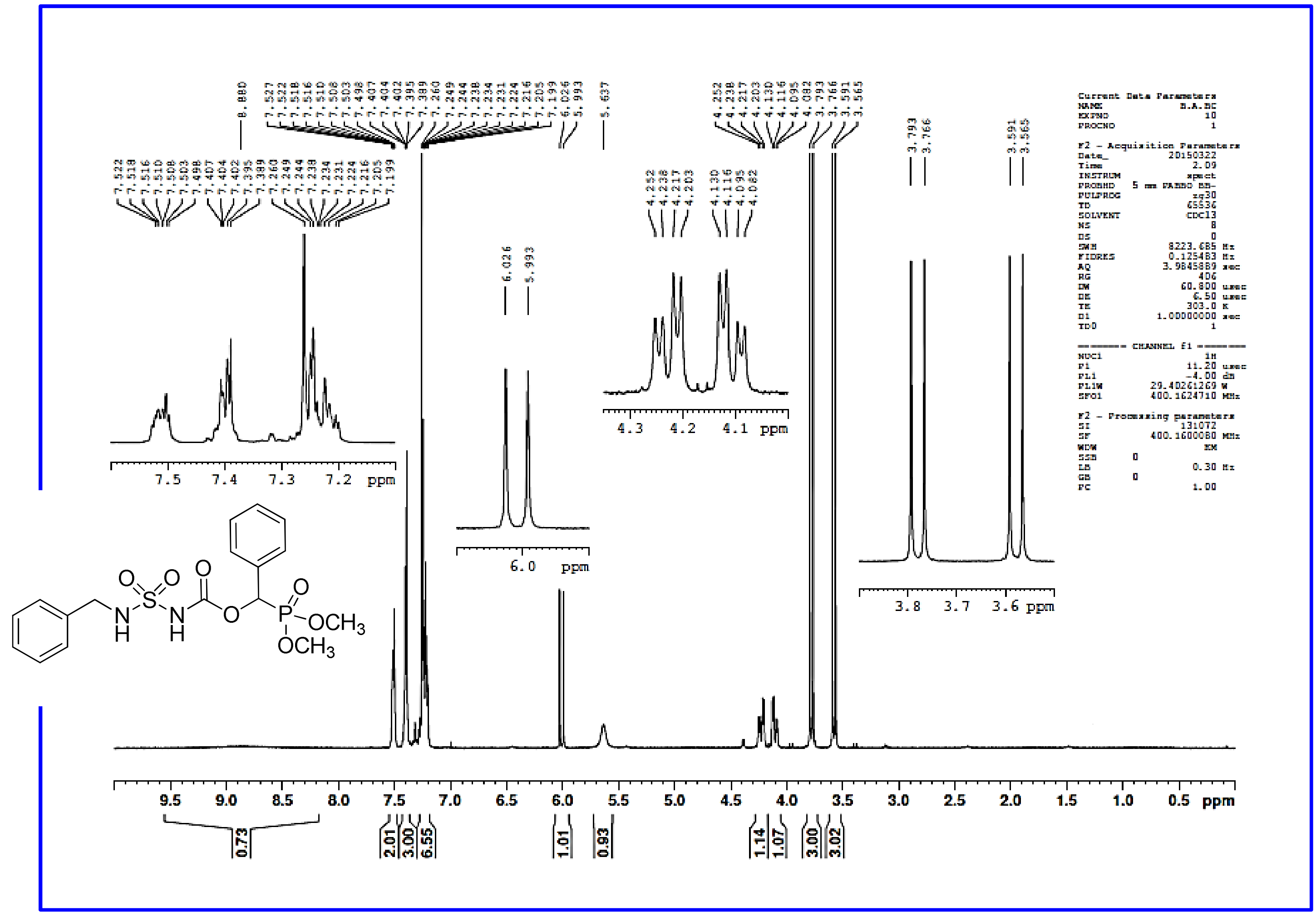

^{1}H NMR Spectrum : (Dimethoxyphosphoryl) (phenyl)methyl *N*-benzylsulfamoylcarbamate **1a**

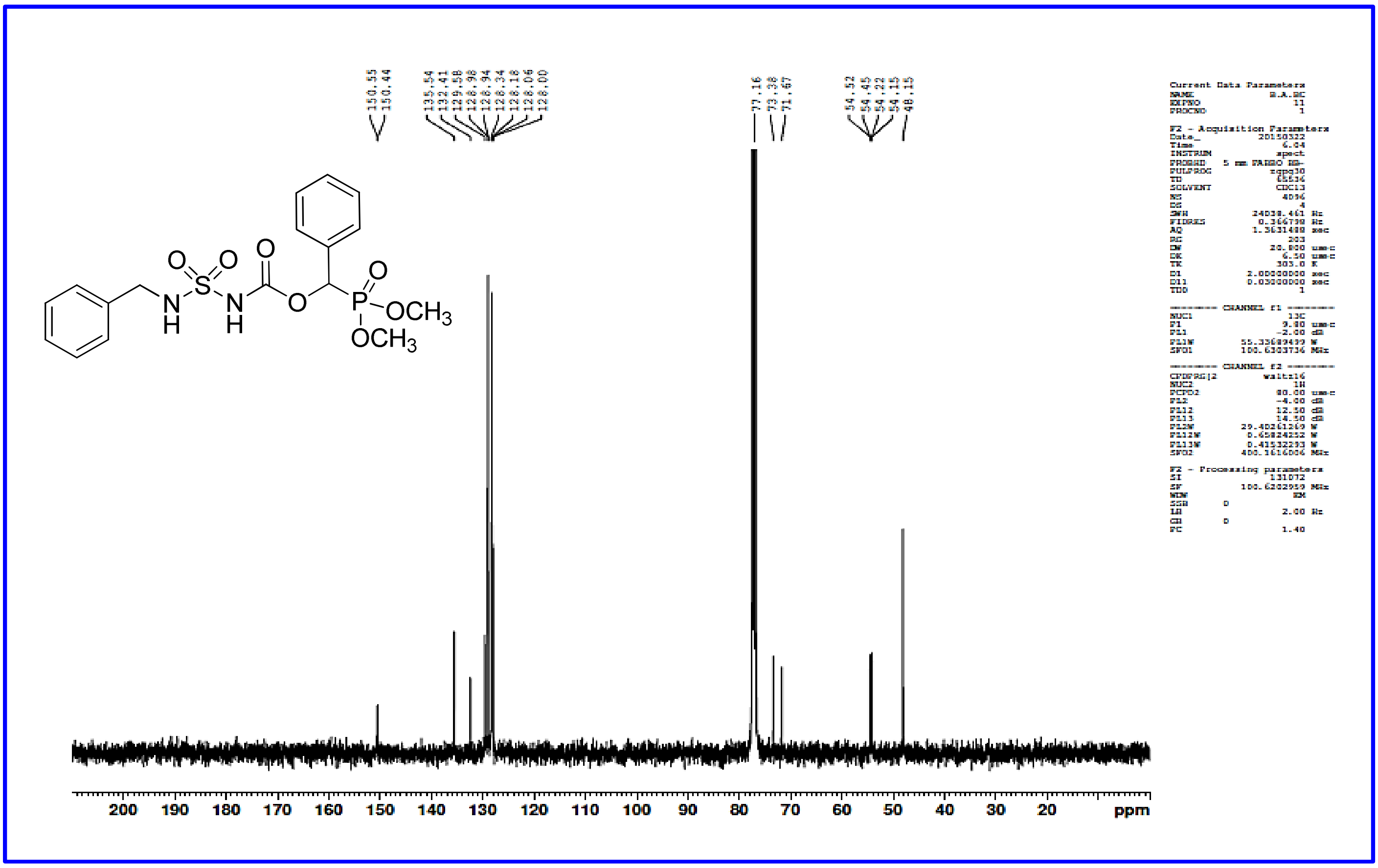

^{13}C NMR Spectrum : (Dimethoxyphosphoryl)(phenyl)methyl *N*-benzylsulfamoylcarbamate **1a**

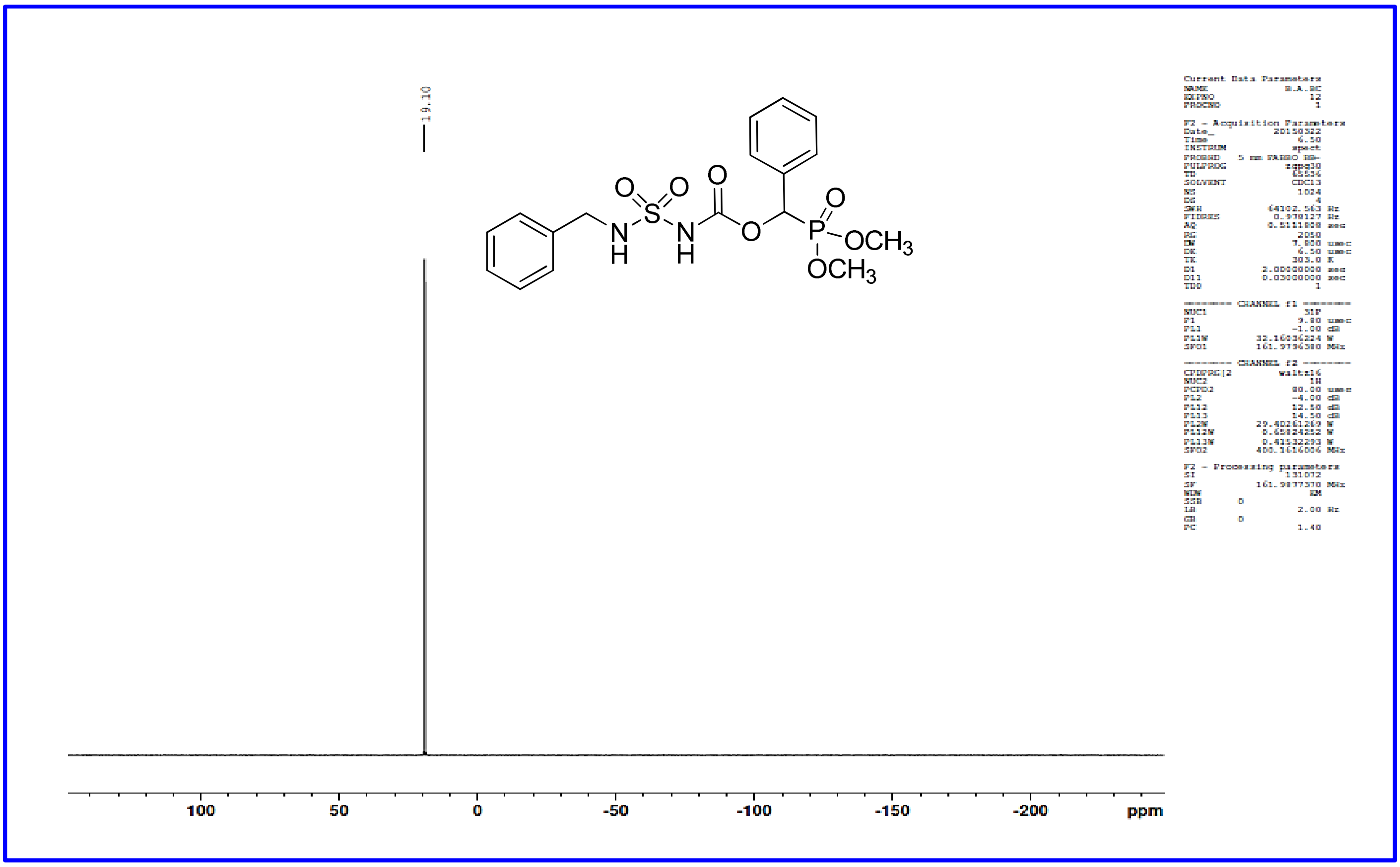

^{31}P NMR Spectrum : (Dimethoxyphosphoryl)(phenyl)methyl *N*-benzylsulfamoylcarbamate **1a**

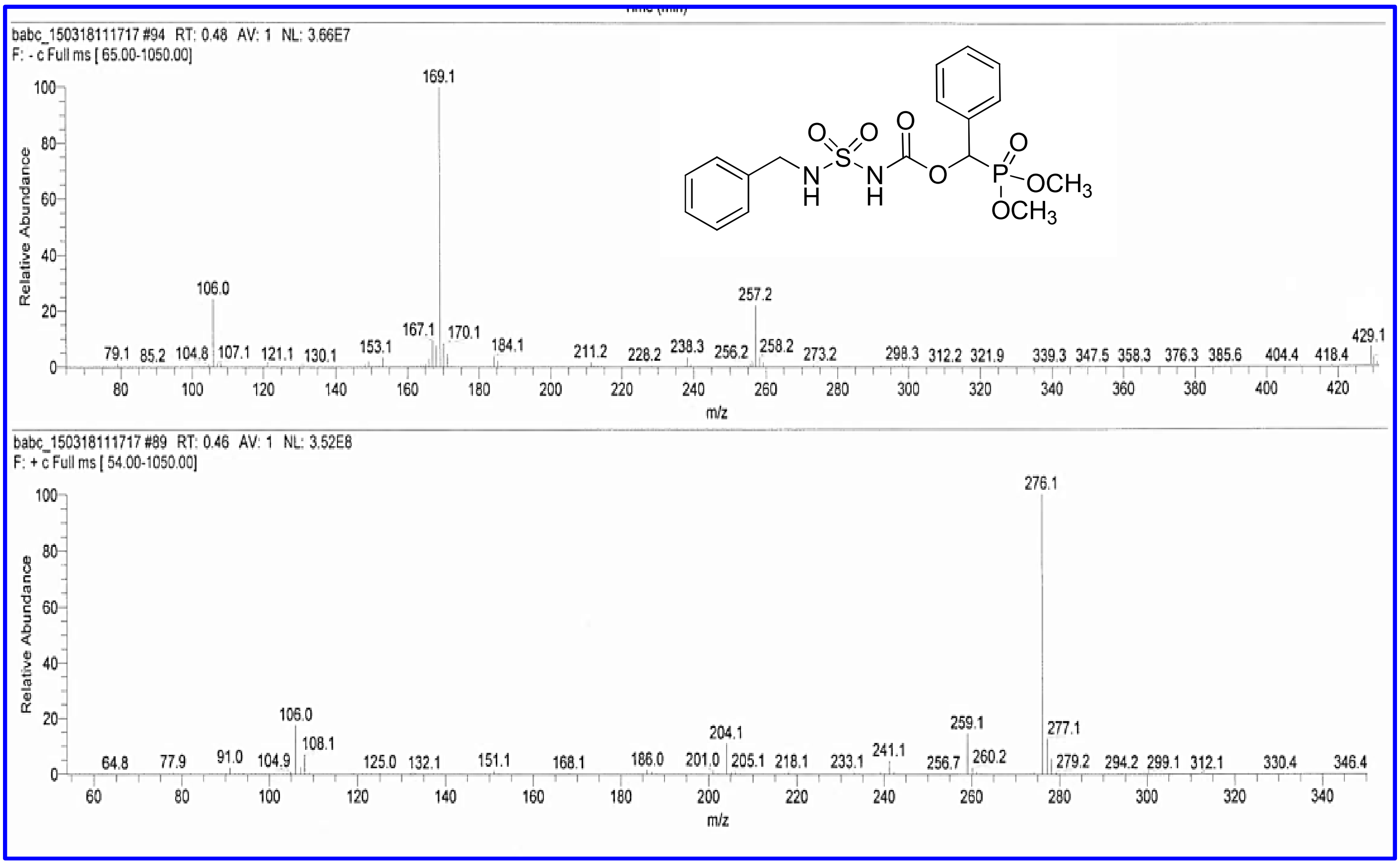

MS : (Dimethoxyphosphoryl)(phenyl)methyl *N*-benzylsulfamoylcarbamate **1a**

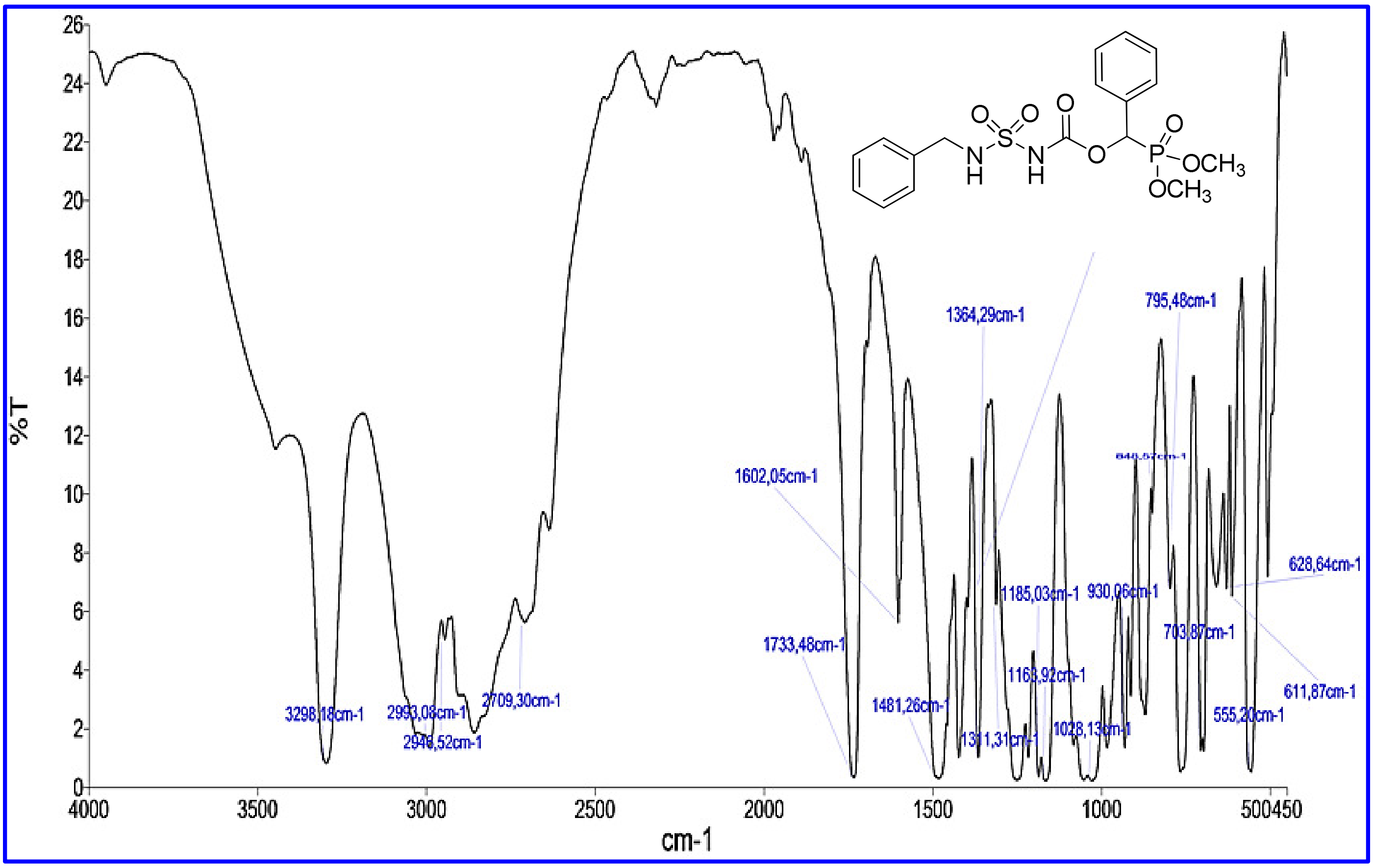

IR Spectrum : (Dimethoxyphosphoryl)(phenyl)methyl *N*-benzylsulfamoylcarbamate **1a**

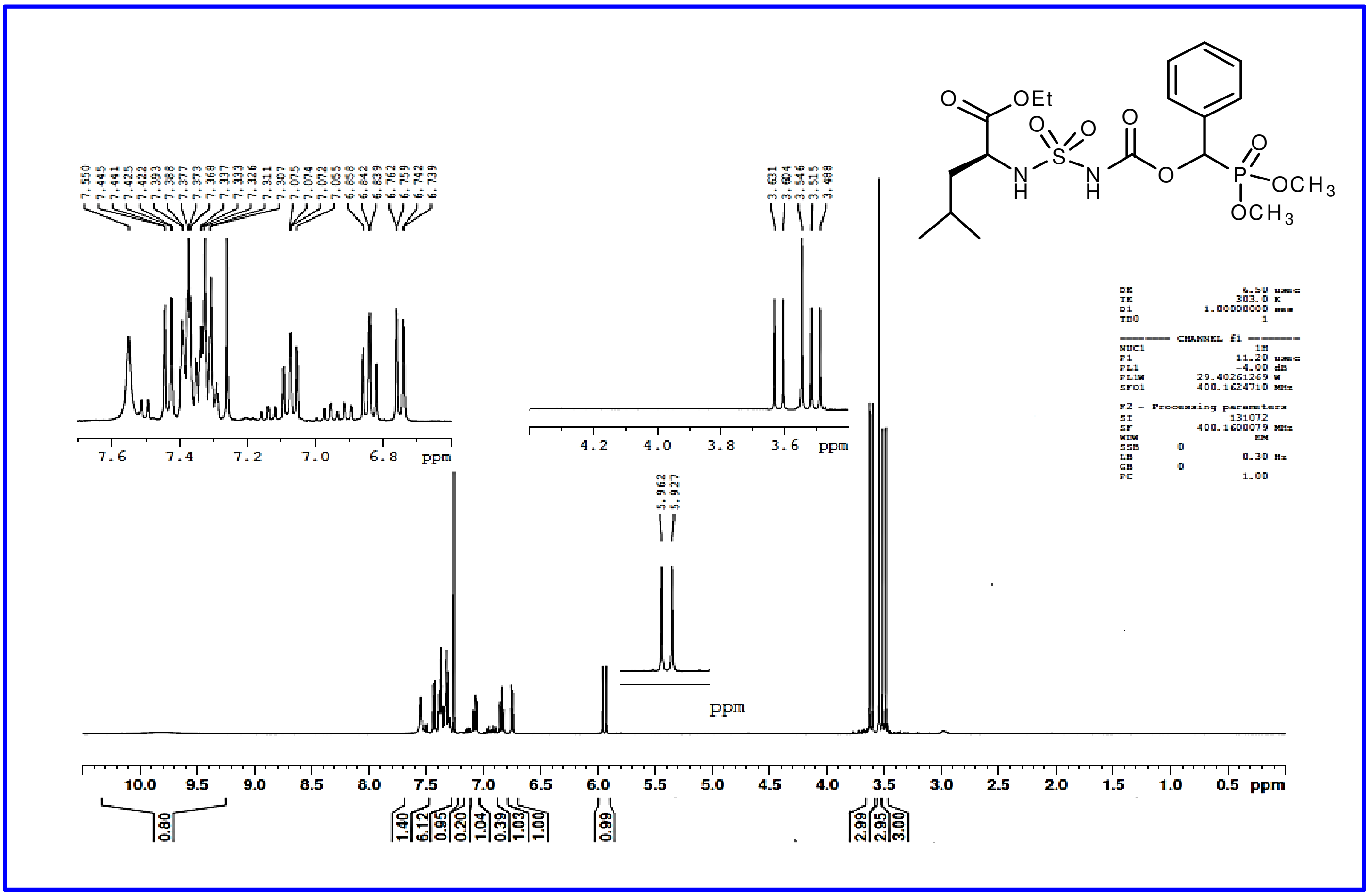

^{1}H NMR Spectrum: (Dimethoxyphosphoryl)(phenyl)methyl *N*-(2-methoxyphenyl)sulfamoylcarbamate **2a**

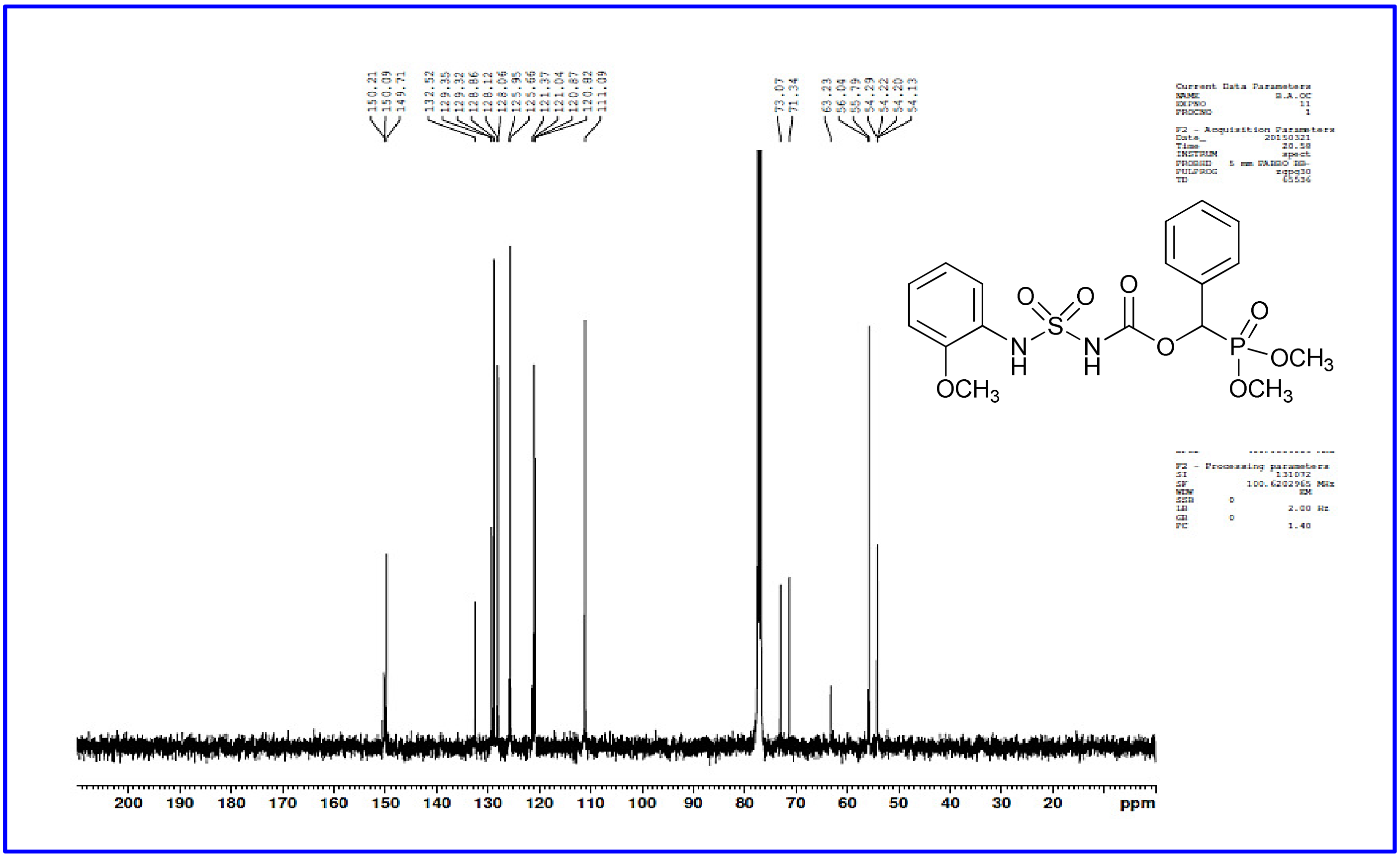

^{13}C NMR Spectrum: (Dimethoxyphosphoryl)(phenyl)methyl *N*-(2-methoxyphenyl)sulfamoylcarbamate **2a**

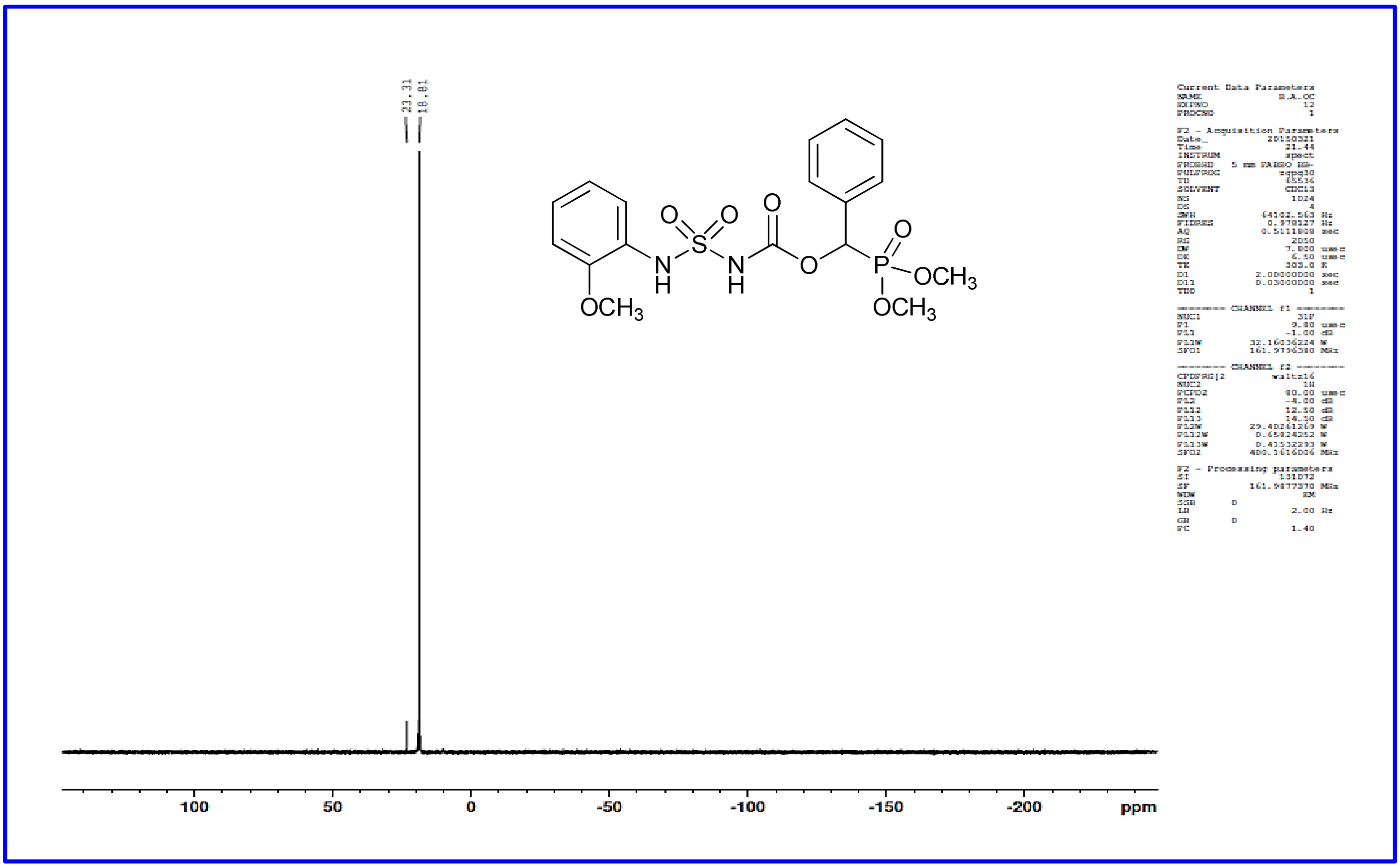

^{31}P NMR Spectrum: (Dimethoxyphosphoryl)(phenyl)methyl *N*-(2-methoxyphenyl)sulfamoylcarbamate **2a**

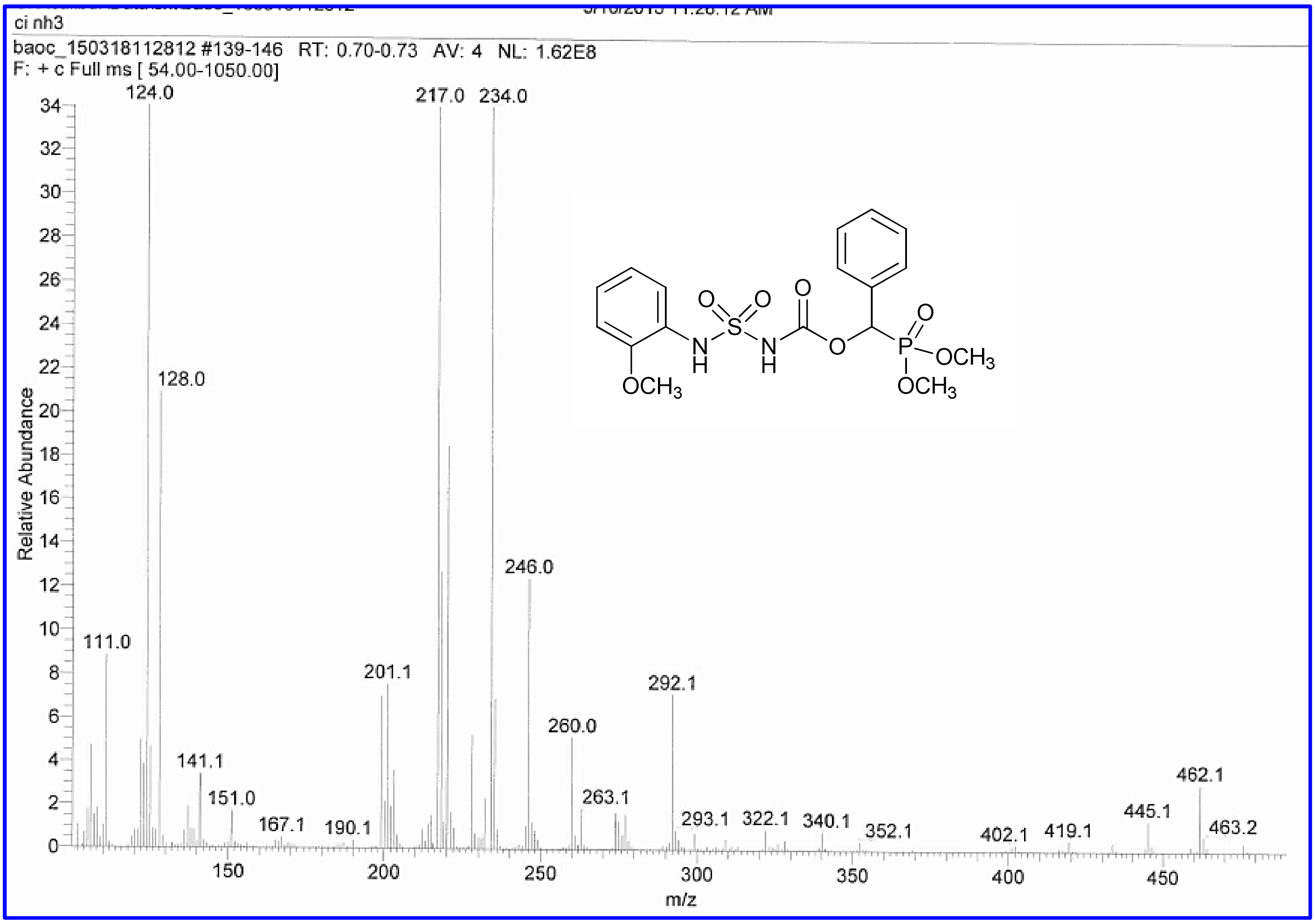

MS: (Dimethoxyphosphoryl)(phenyl)methyl *N*-(2-methoxyphenyl)sulfamoylcarbamate **2a**

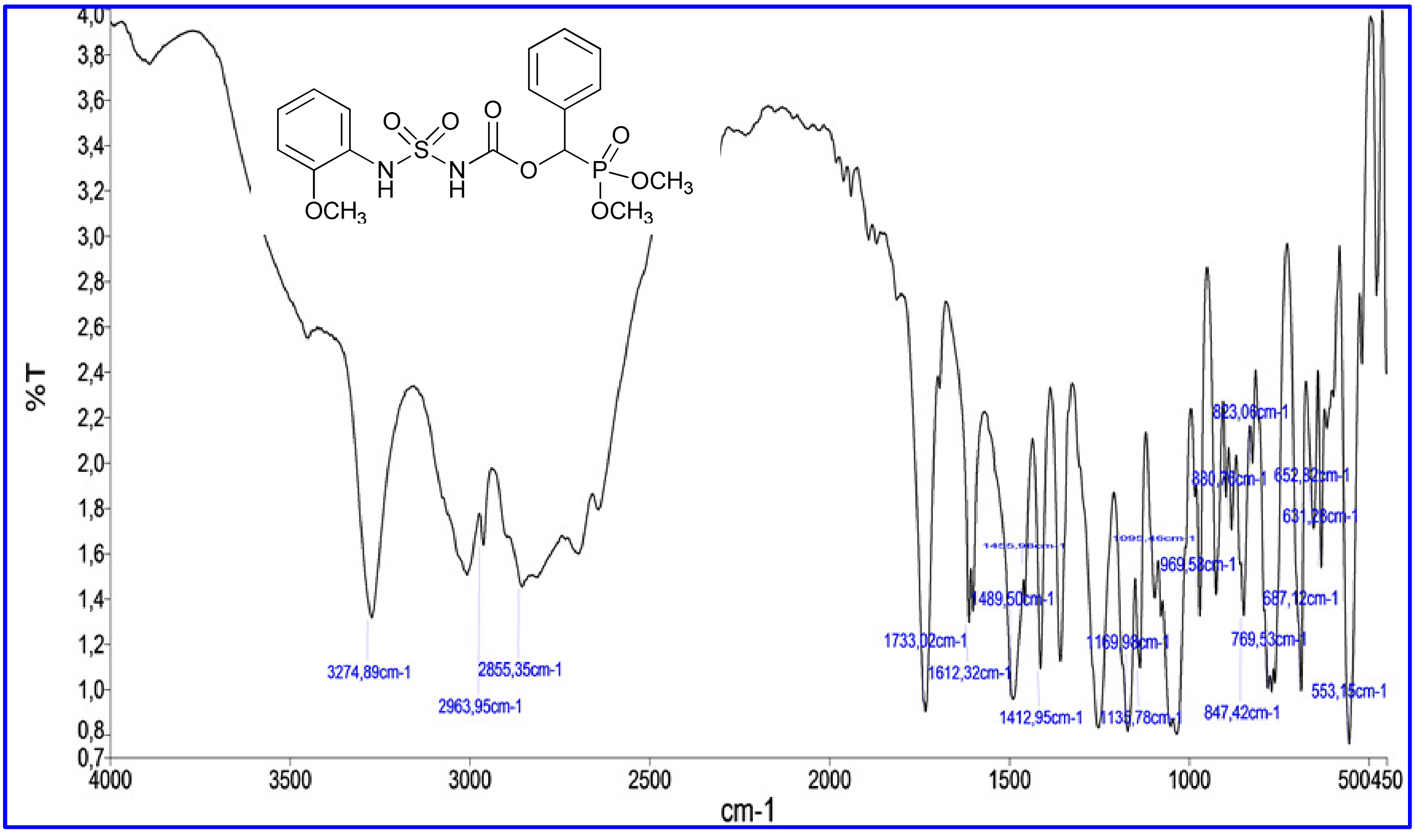

IR Spectrum: (Dimethoxyphosphoryl)(phenyl)methyl *N*-(2-methoxyphenyl)sulfamoylcarbamate **2a**

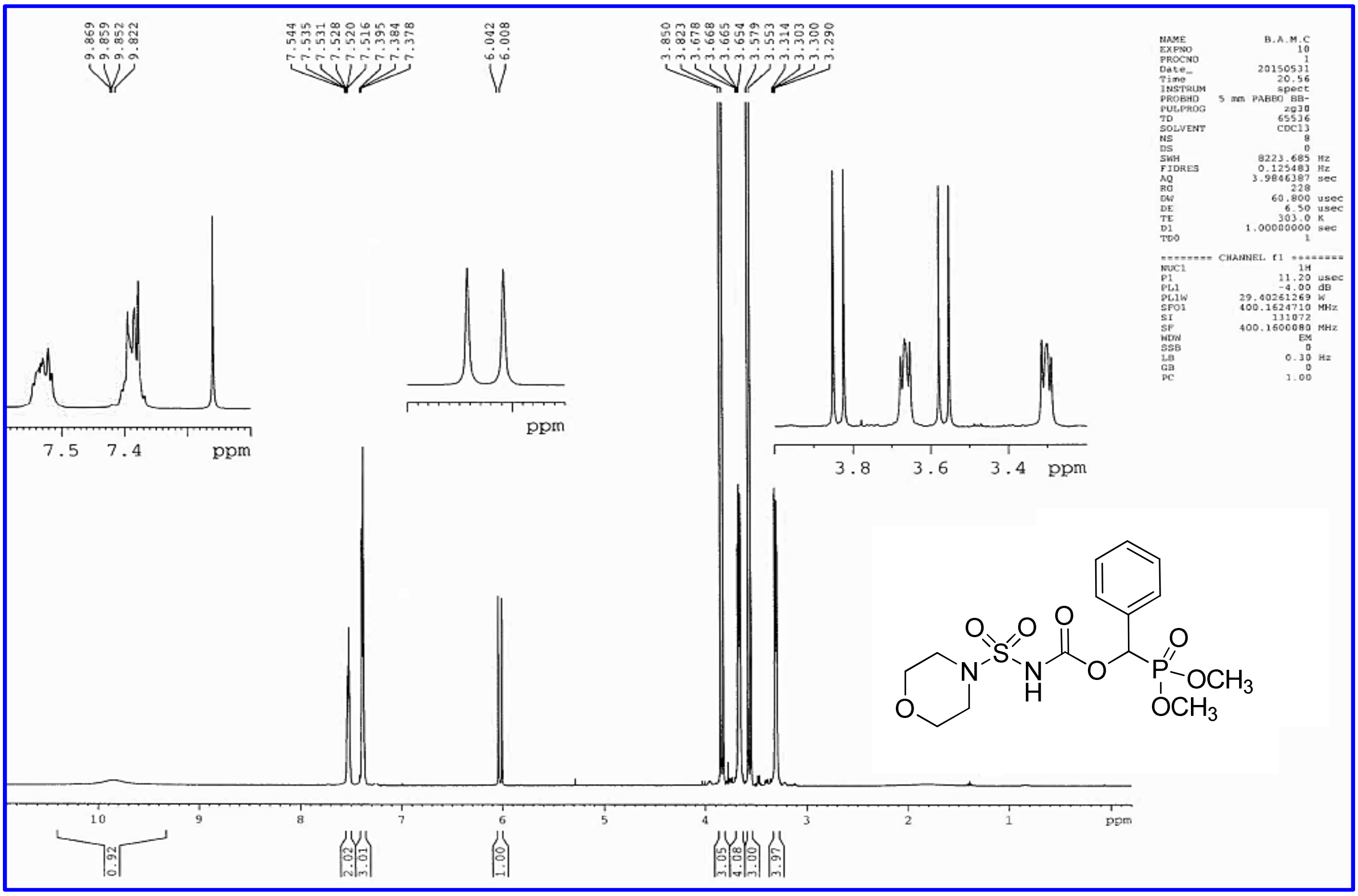

^{1}H NMR Spectrum : (dimethoxyphosphoryl)(phenyl)methyl(morpholinosulfonyl)carbamate **3a**

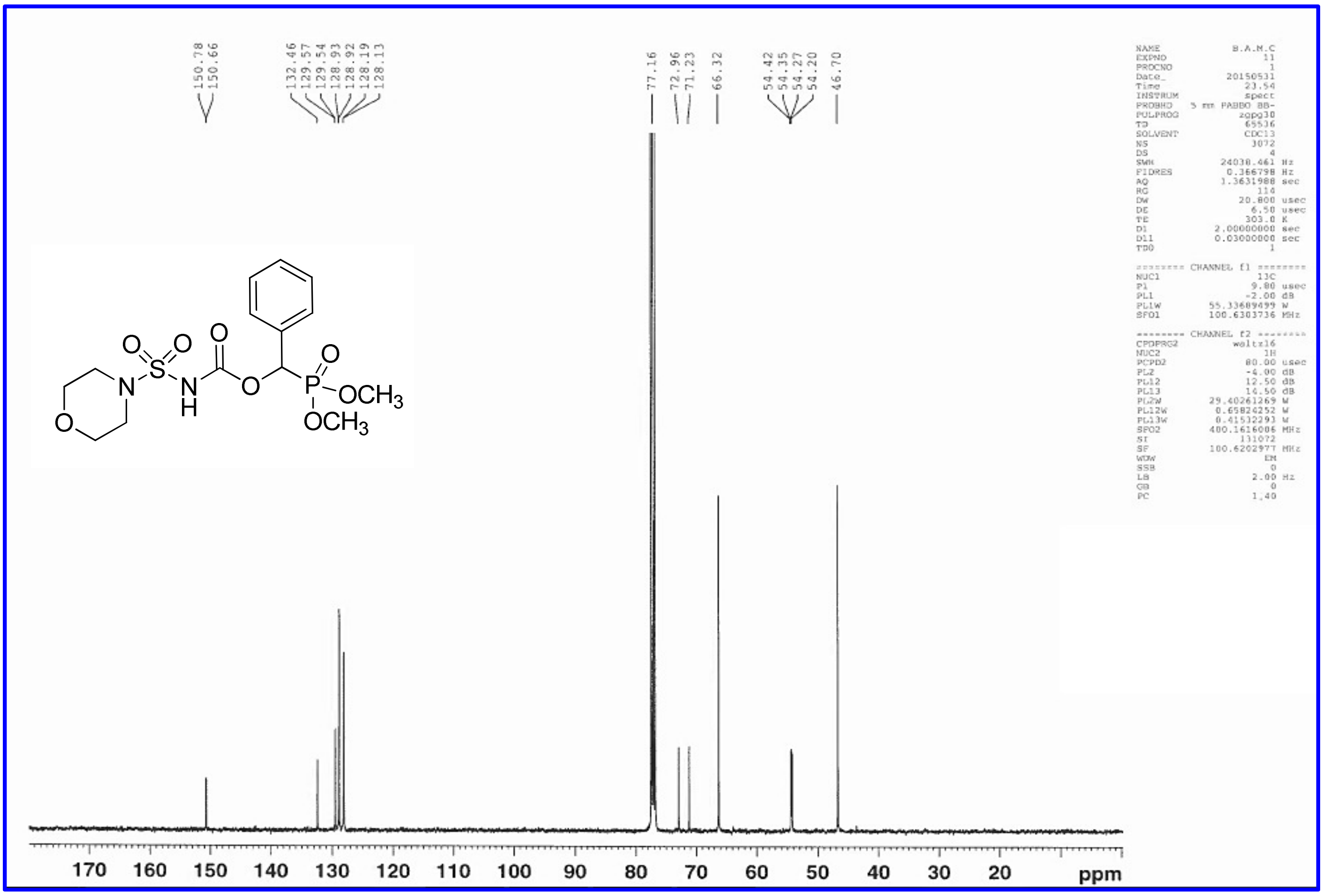

^{13}C NMR Spectrum : (Dimethoxyphosphoryl)(phenyl)methyl(morpholinosulfonyl)carbamate **3a**

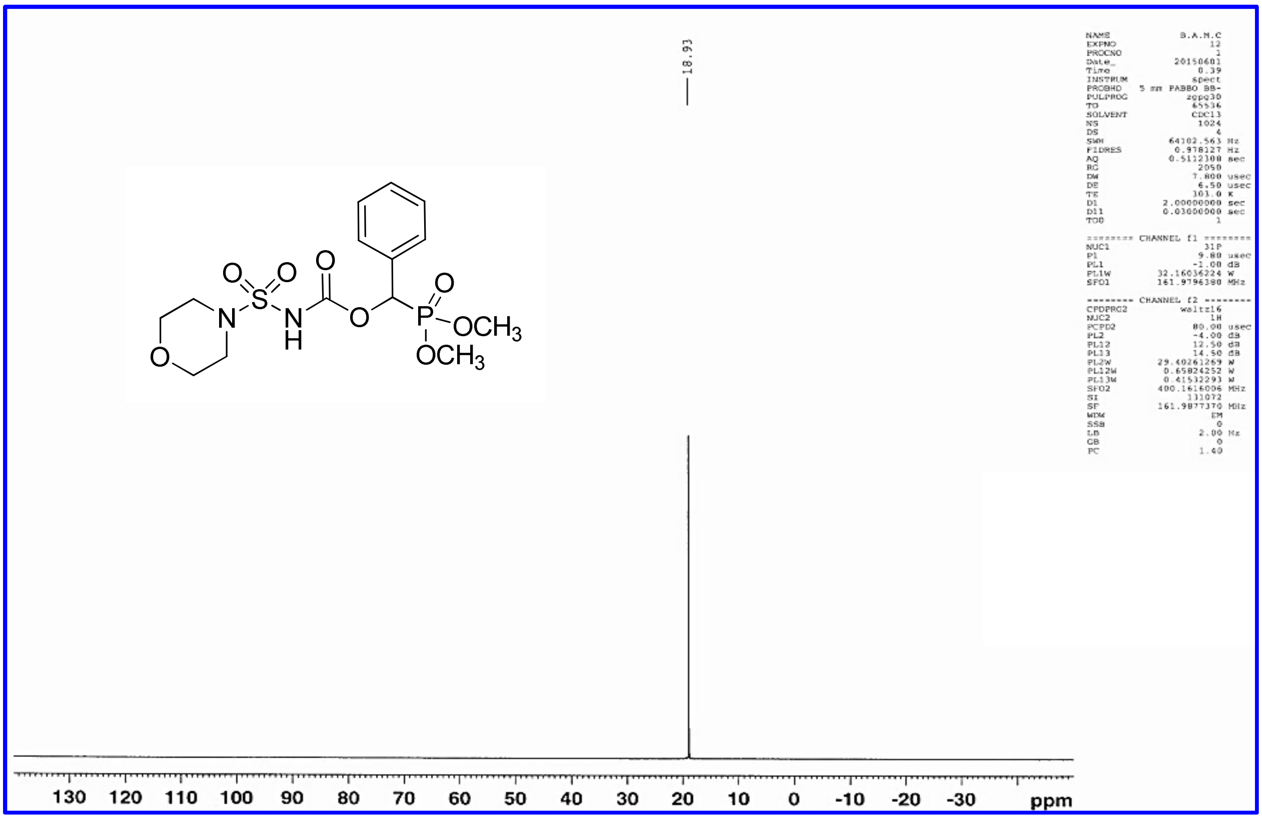

^{31}P NMR Spectrum : (Dimethoxyphosphoryl)(phenyl)methyl(morpholinosulfonyl)carbamate **3a**

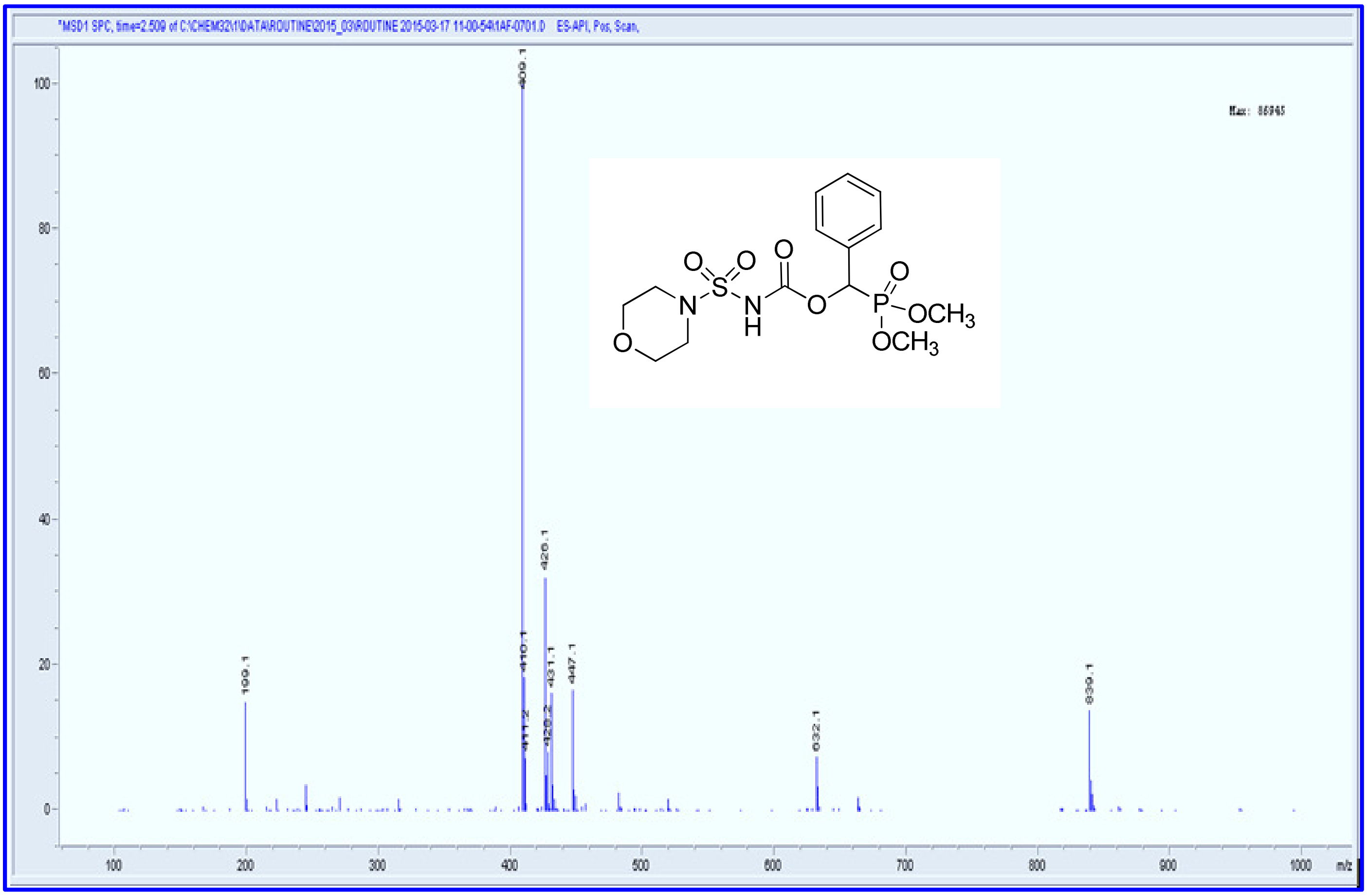

MS: (Dimethoxyphosphoryl)(phenyl)methyl(morpholinosulfonyl)carbamate **3a**

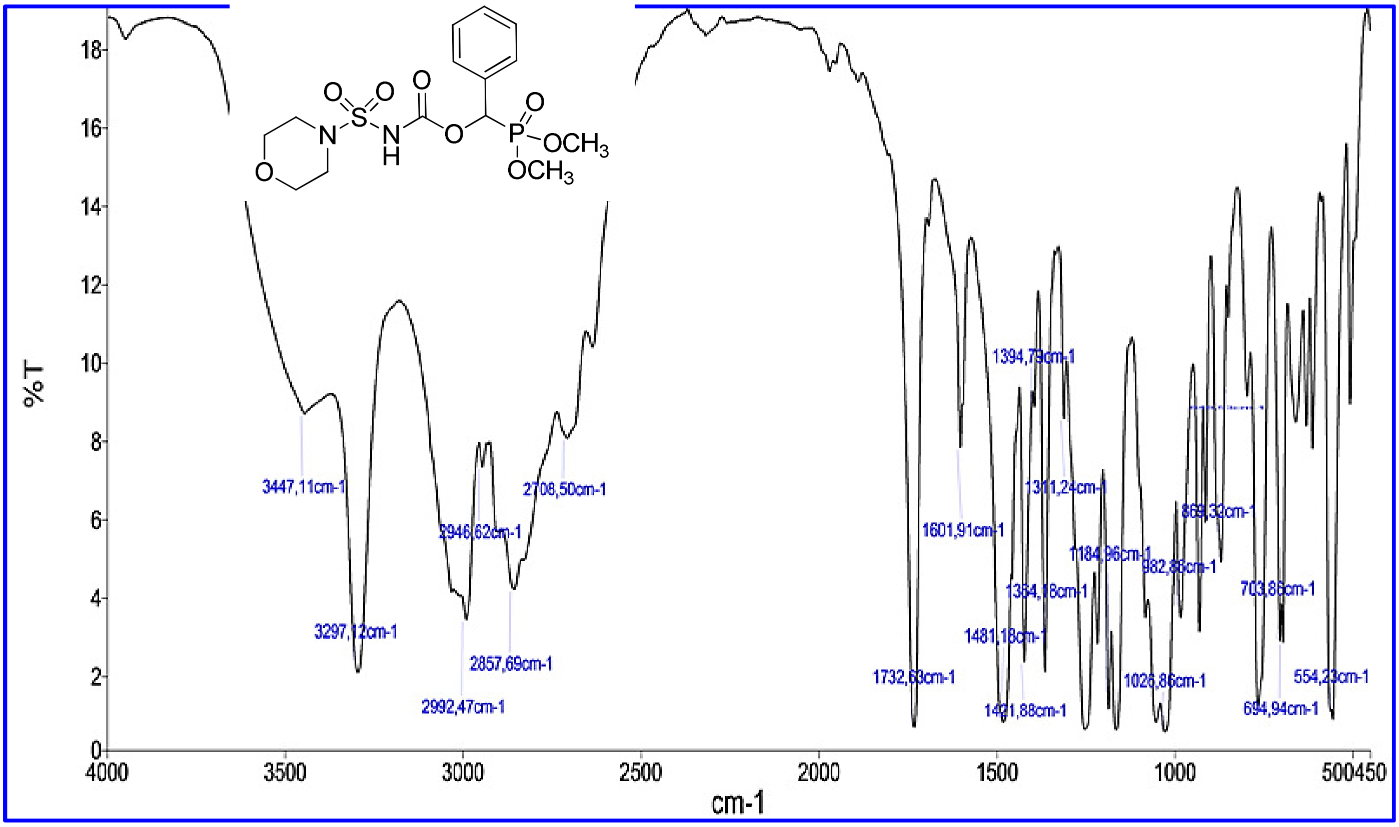

IR Spectrum : (Dimethoxyphosphoryl)(phenyl)methyl(morpholinosulfonyl)carbamate **3a**

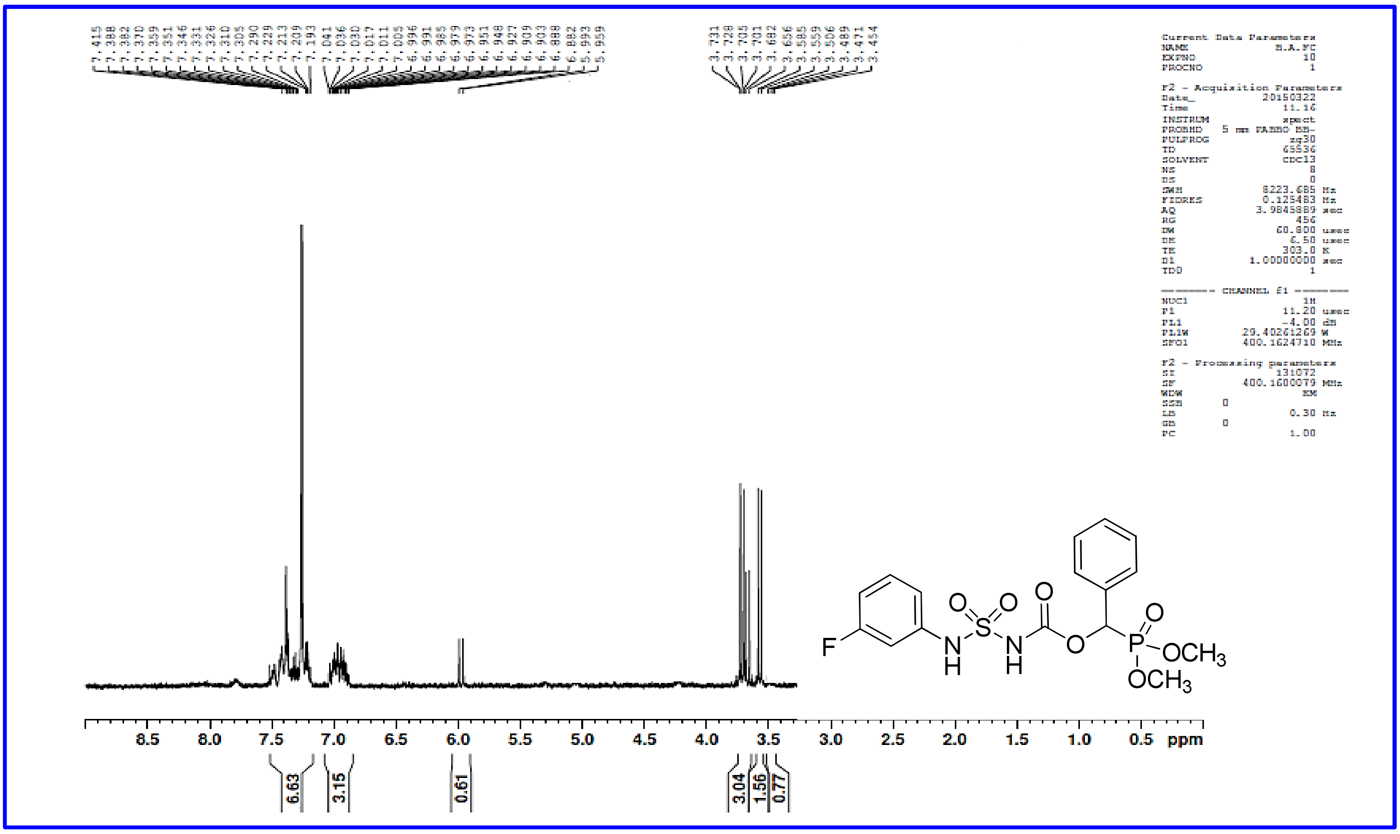

^{1}H NMR Spectrum : (Dimethoxyphosphoryl)(phenyl)methyl *N*-(3-fluorophenyl)sulfamoylcarbamate **4a**

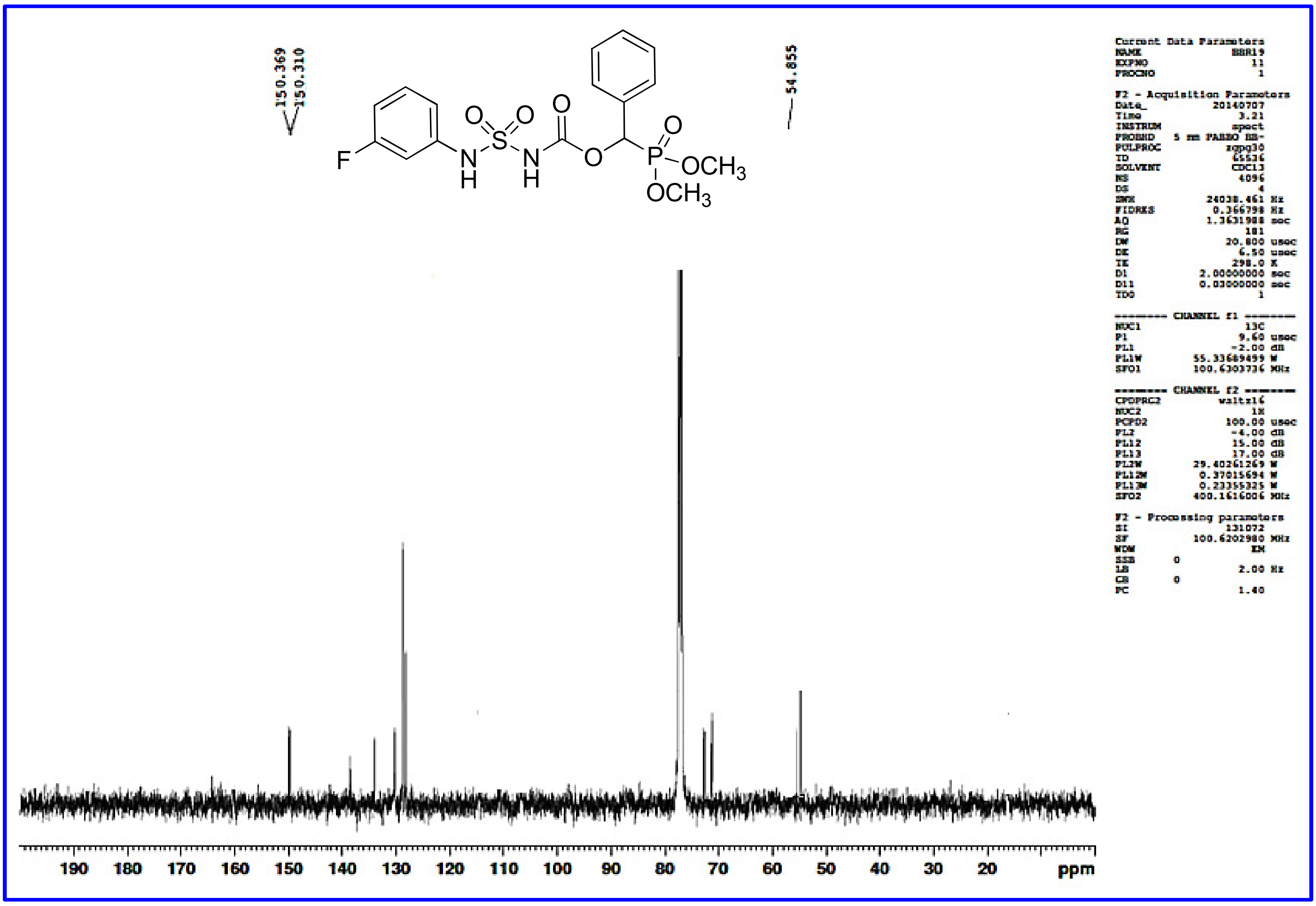

^{13}C NMR Spectrum : (Dimethoxyphosphoryl)(phenyl)methyl *N*-(3-fluorophenyl)sulfamoylcarbamate **4a**

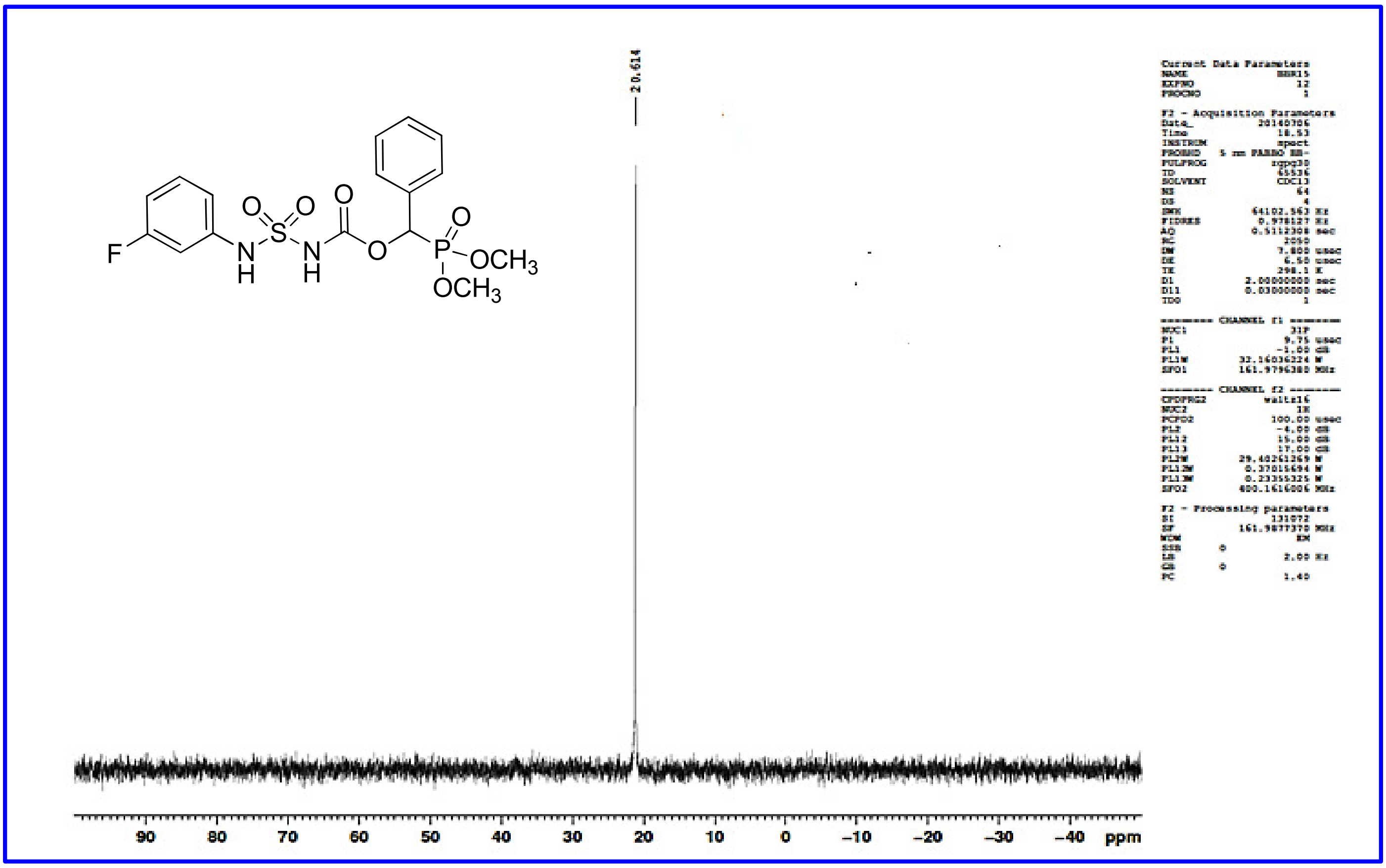

^{31}P NMR Spectrum : (Dimethoxyphosphoryl)(phenyl)methyl *N*-(3-fluorophenyl)sulfamoylcarbamate **4a**

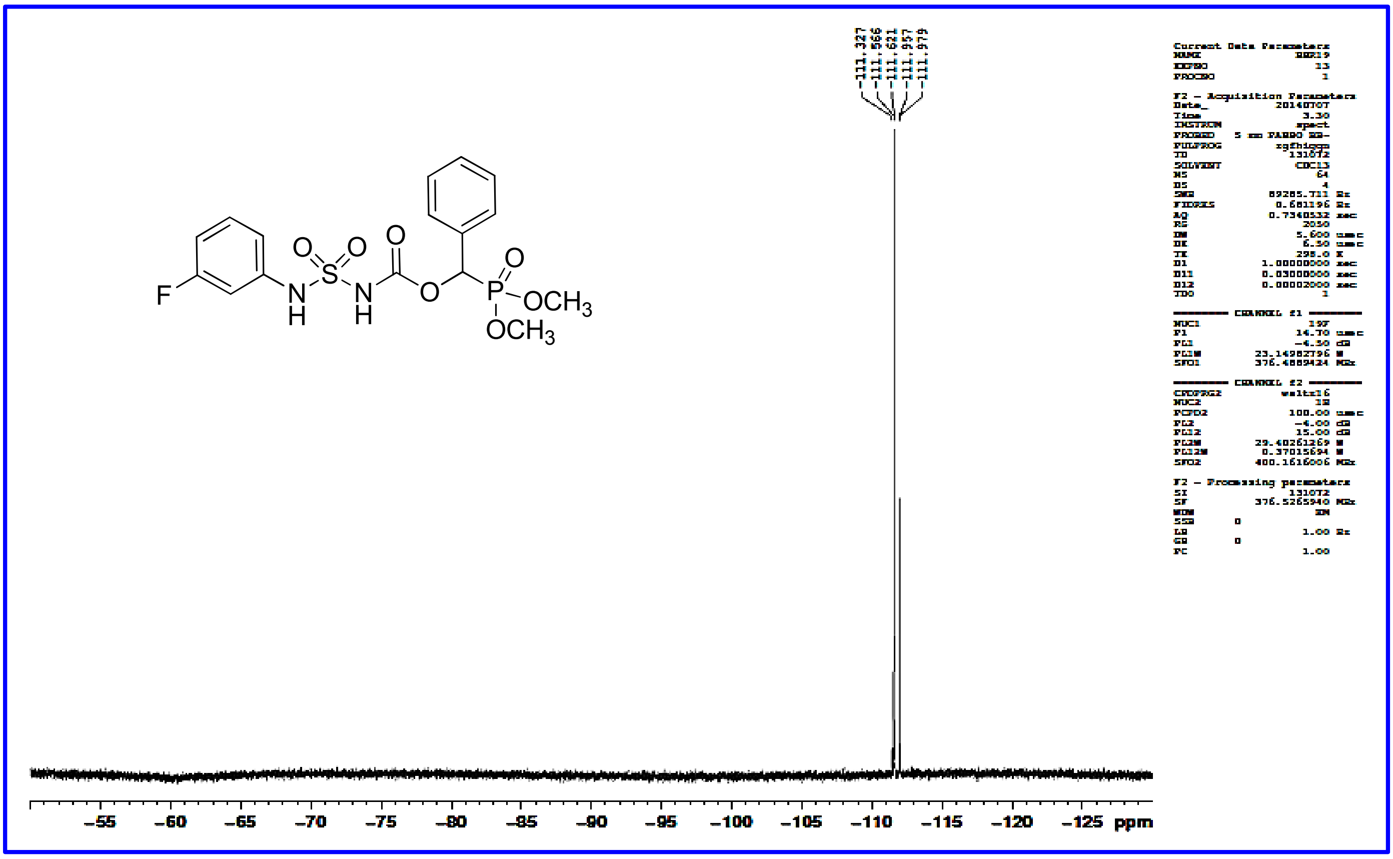

^{19}F NMR Spectrum : (Dimethoxyphosphoryl)(phenyl)methyl *N*-(3-fluorophenyl)sulfamoylcarbamate **4a**

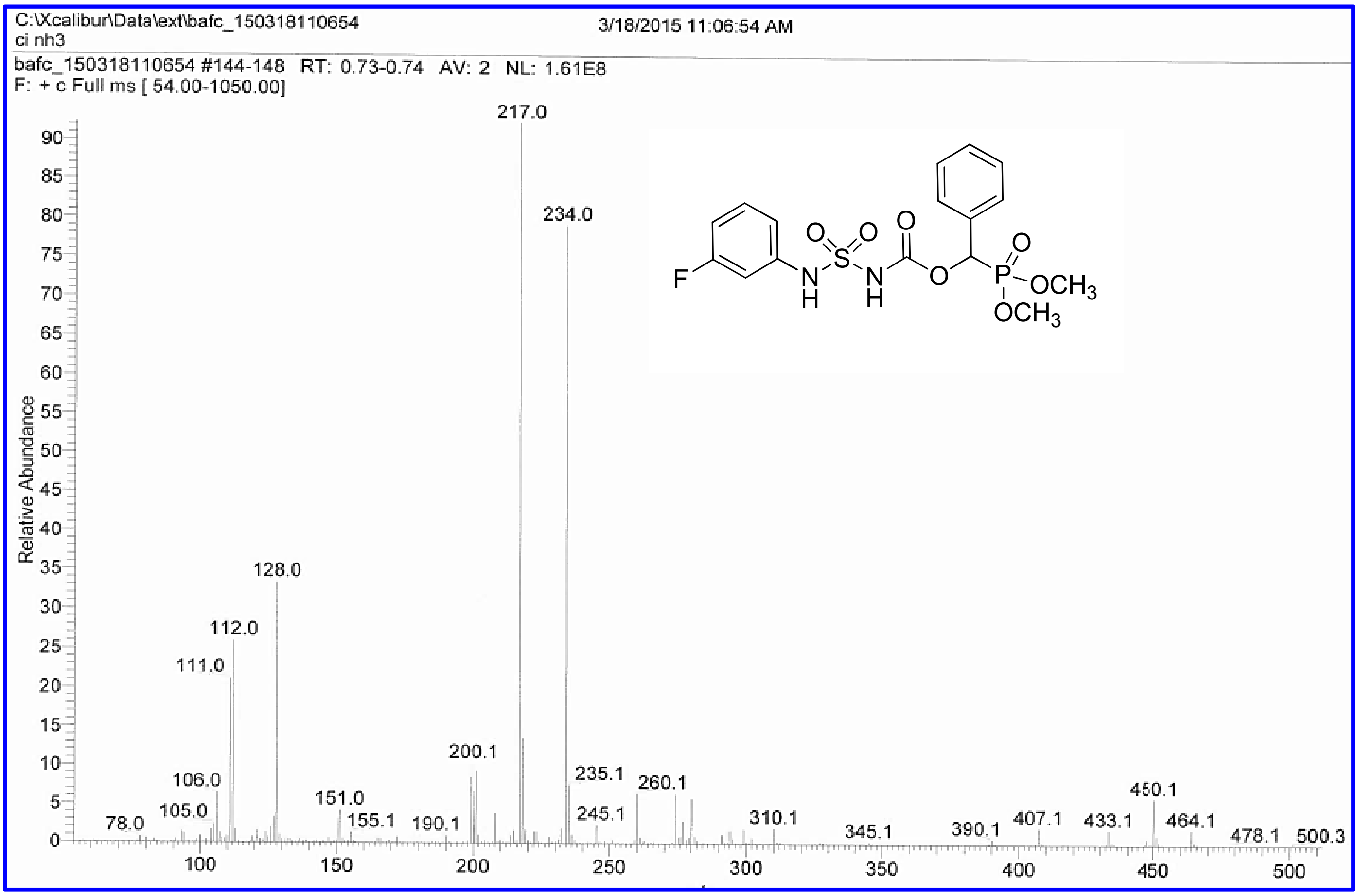

MS : (Dimethoxyphosphoryl)(phenyl)methyl *N*-(3-fluorophenyl)sulfamoylcarbamate **4a**

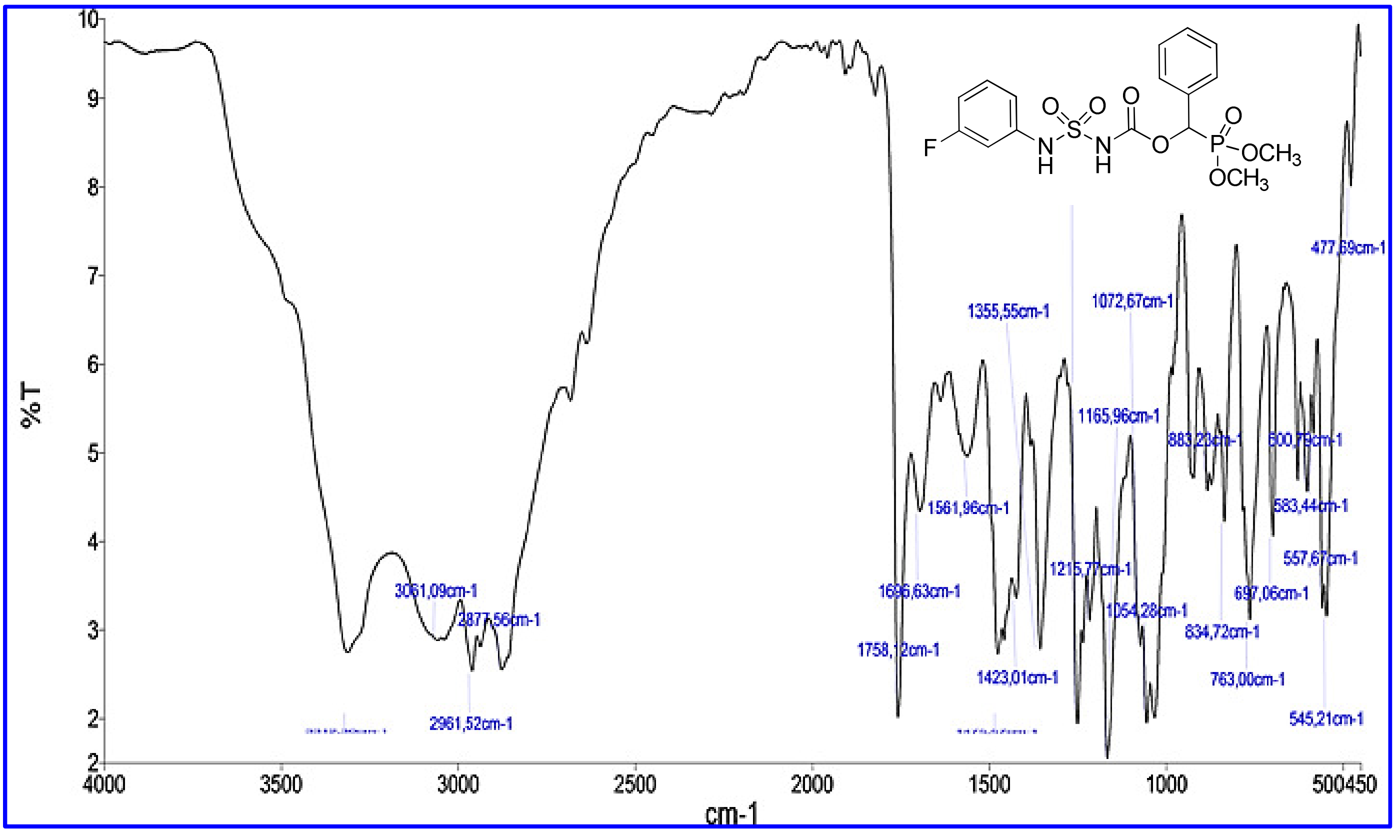

IR Spectrum : (Dimethoxyphosphoryl)(phenyl)methyl *N*-(3-fluorophenyl)sulfamoylcarbamate **4a**

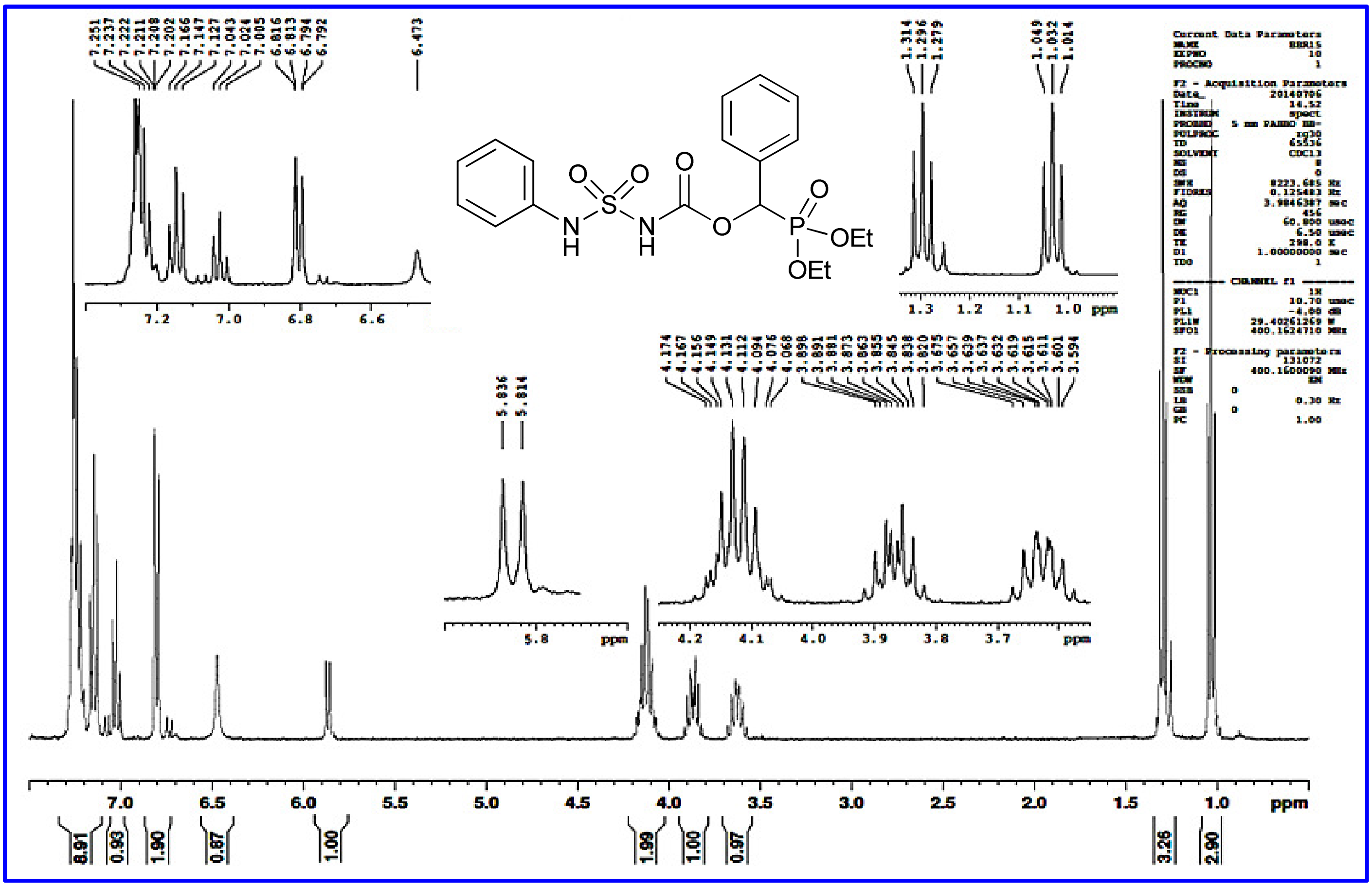

^{1}H NMR Spectrum: (Diethoxyphosphoryl)(phenyl)methyl *N*-phenylsulfamoylcarbamate **5a**

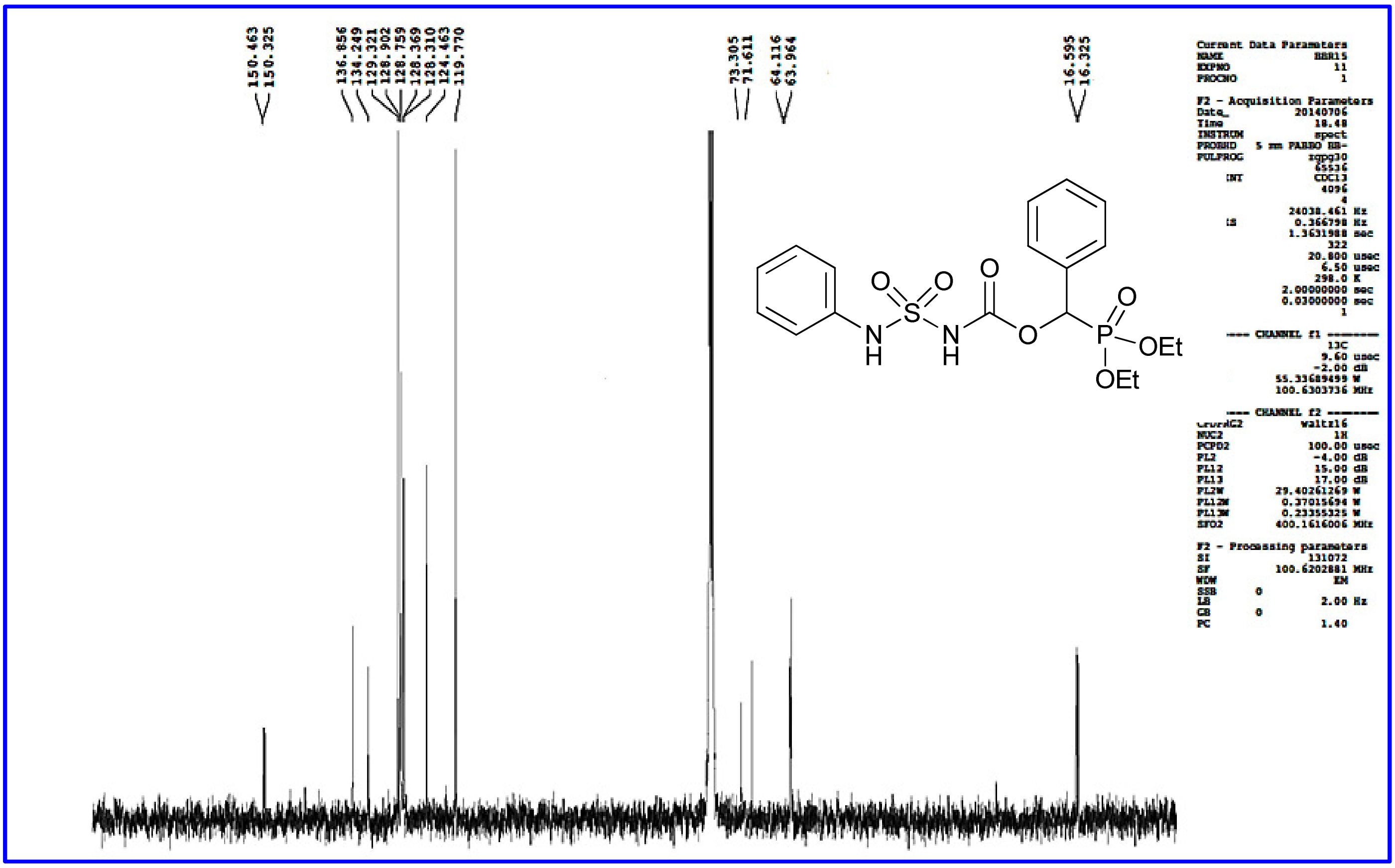

^{13}C NMR Spectrum: (Diethoxyphosphoryl)(phenyl)methyl *N*-phenylsulfamoylcarbamate **5a**

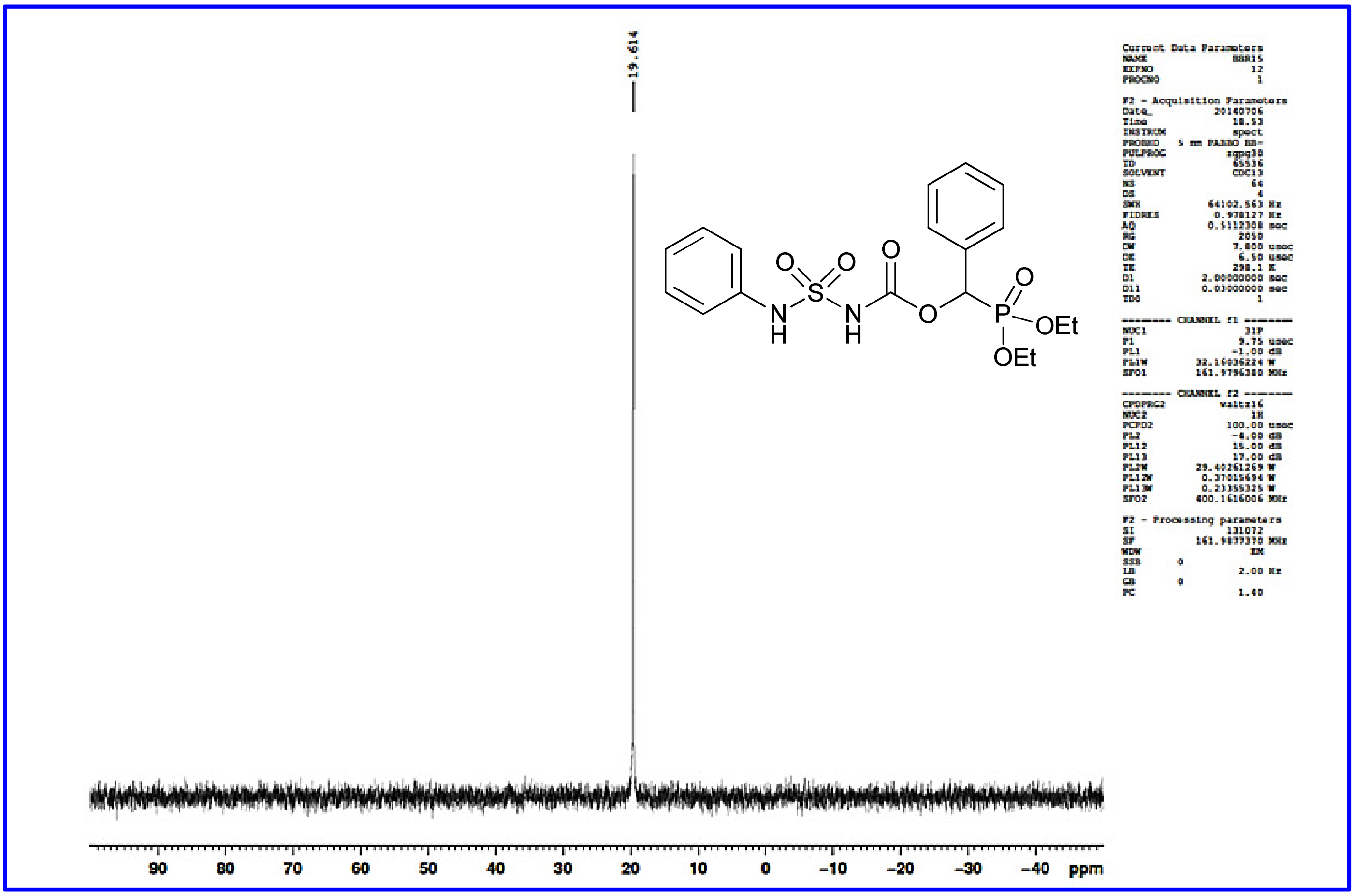

^{31}P NMR Spectrum: (Diethoxyphosphoryl)(phenyl)methyl *N*-phenylsulfamoylcarbamate **5a**

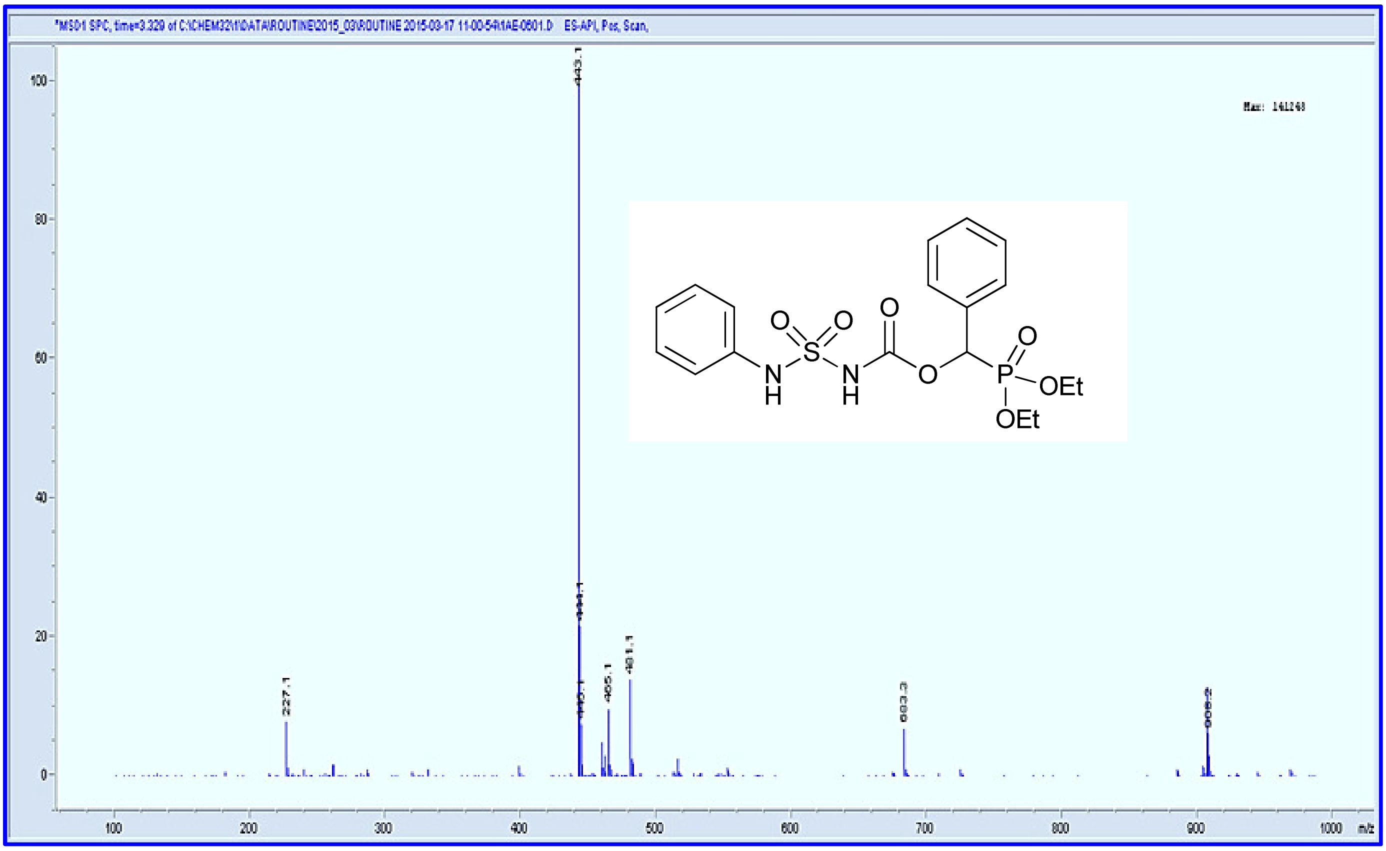

MS: (Diethoxyphosphoryl)(phenyl)methyl *N*-phenylsulfamoylcarbamate **5a**

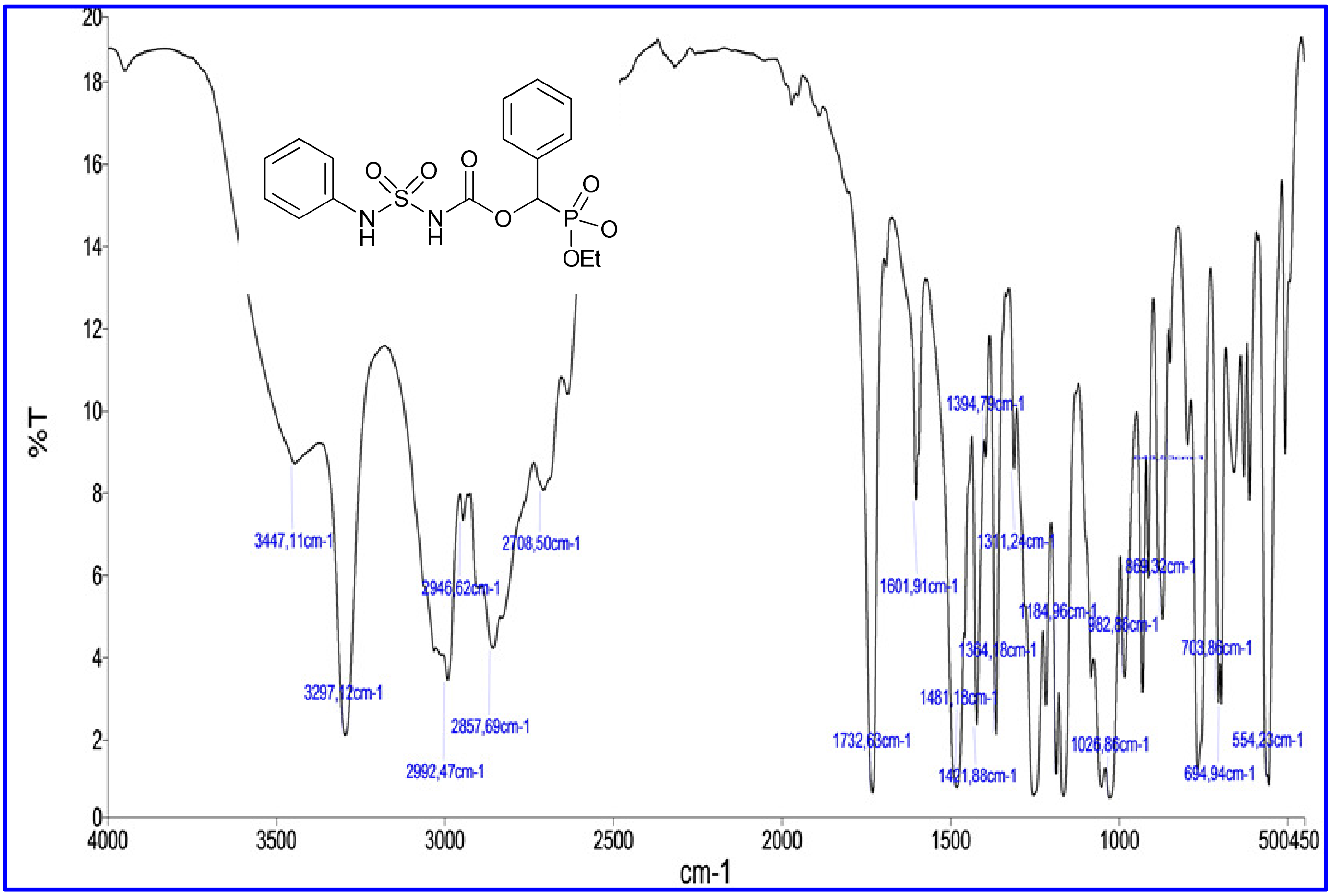

IR Spectrum: (Diethoxyphosphoryl)(phenyl)methyl *N*-phenylsulfamoylcarbamate **5a**

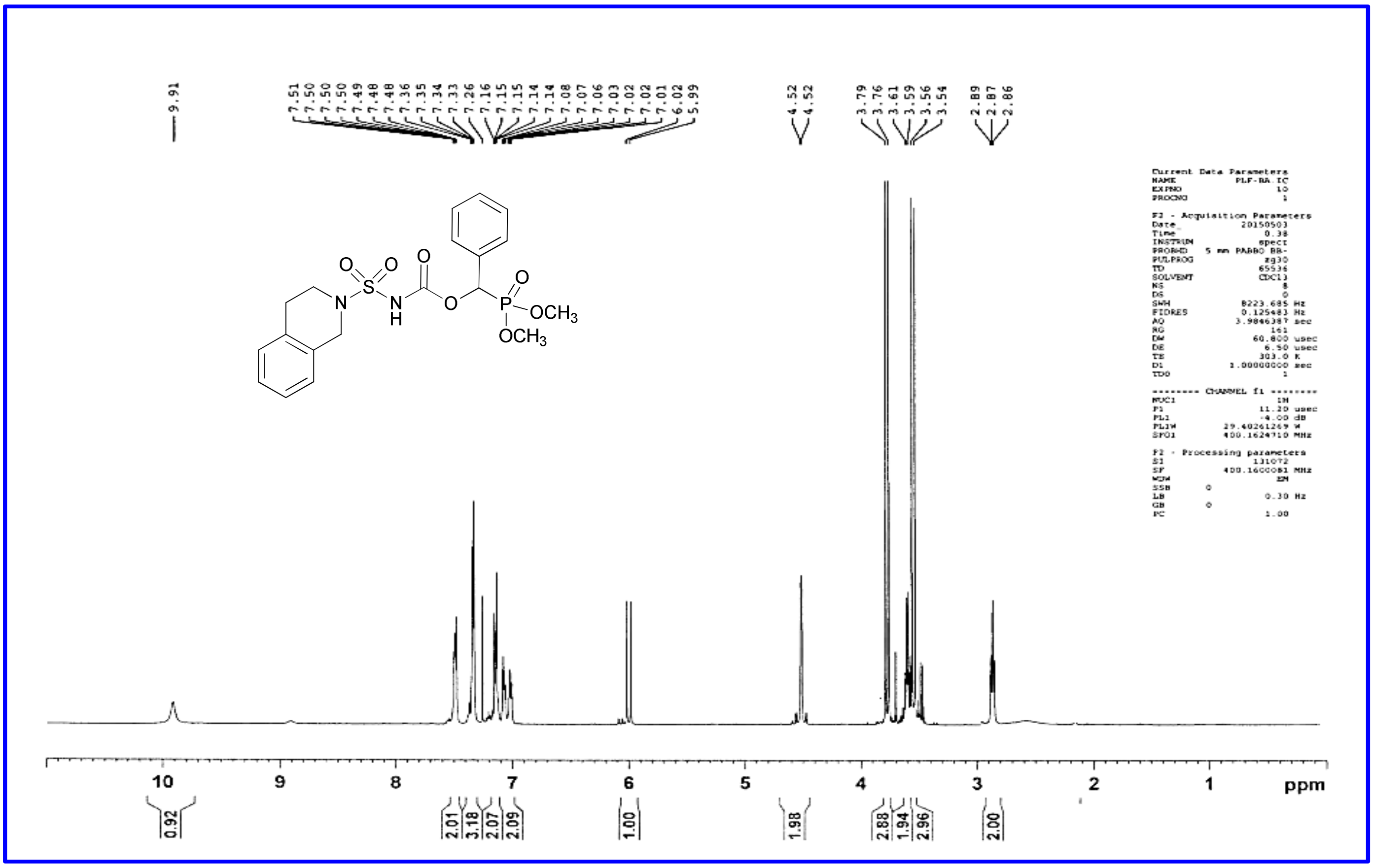

^{1}H NMR Spectrum: (Dimethoxyphosphoryl)(phenyl)methyl (3,4-dihydroisoquinolin-2(1*H*)-yl)sulfonylcarbamate **6a**

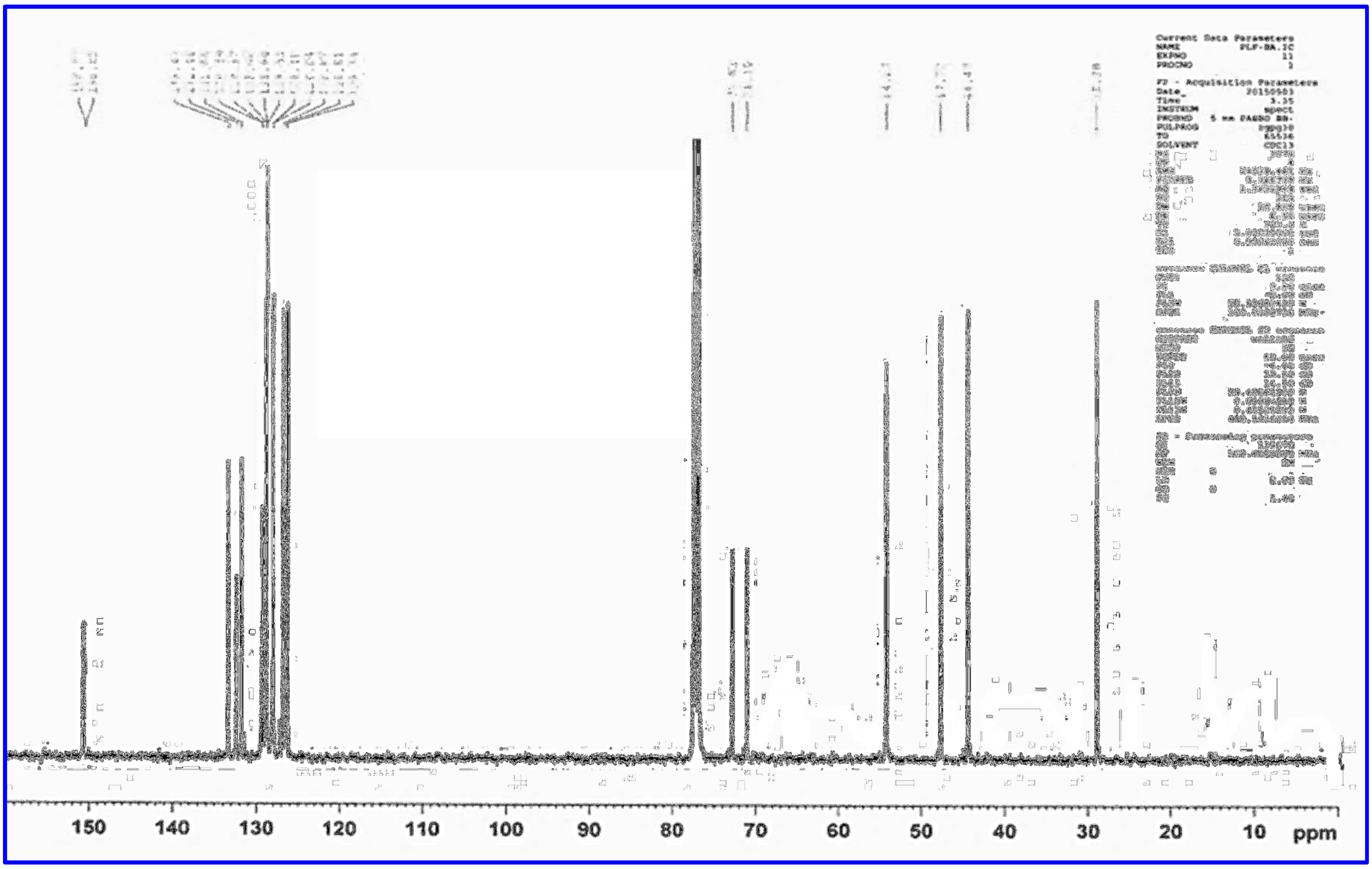

^{13}C NMR Spectrum: (Dimethoxyphosphoryl)(phenyl)methyl (3,4-dihydroisoquinolin-2(*1H*)-yl)sulfonylcarbamate **6a**

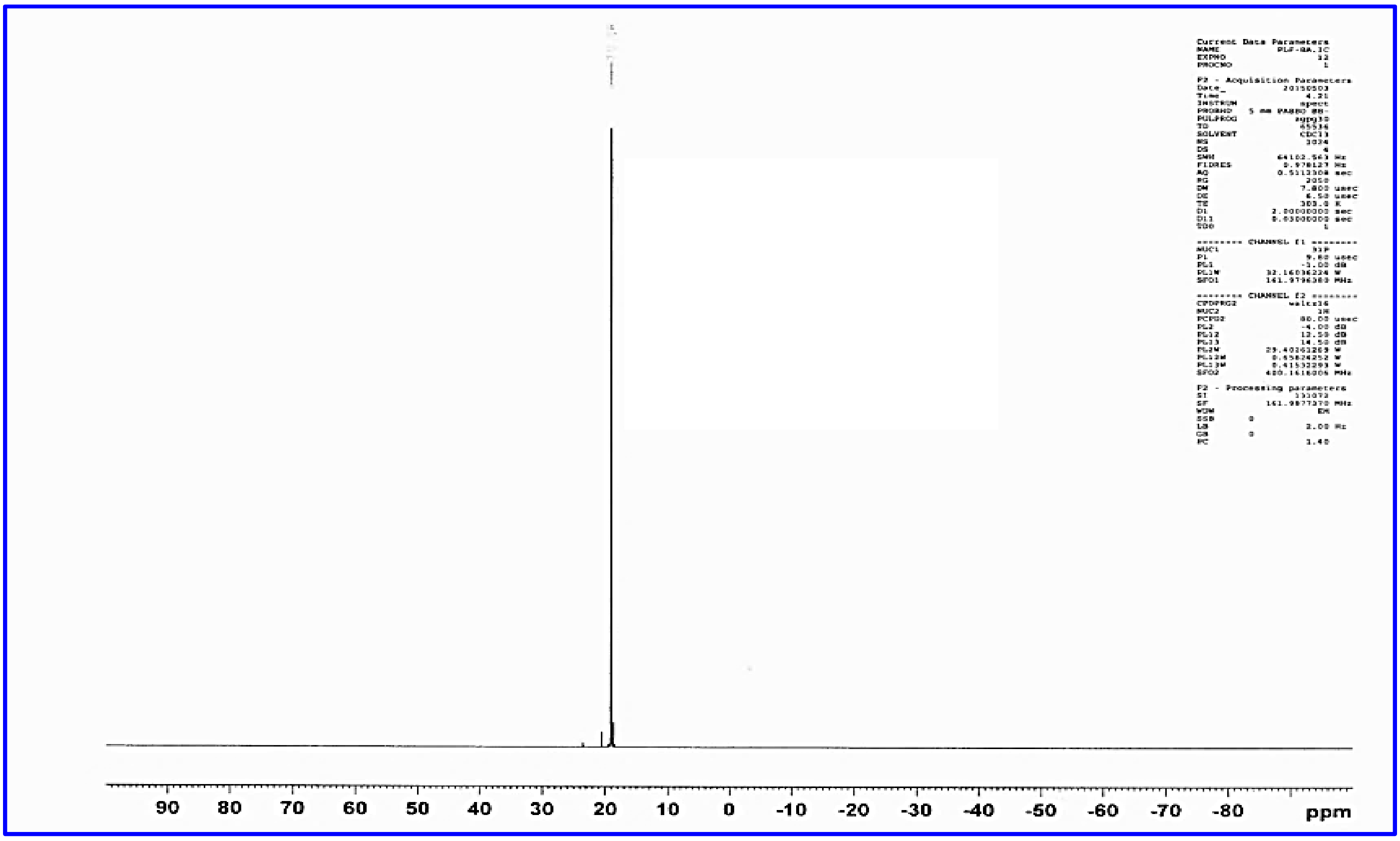

^{31}P NMR Spectrum: (Dimethoxyphosphoryl)(phenyl)methyl (3,4-dihydroisoquinolin-2(1*H*)-yl)sulfonylcarbamate **6a**

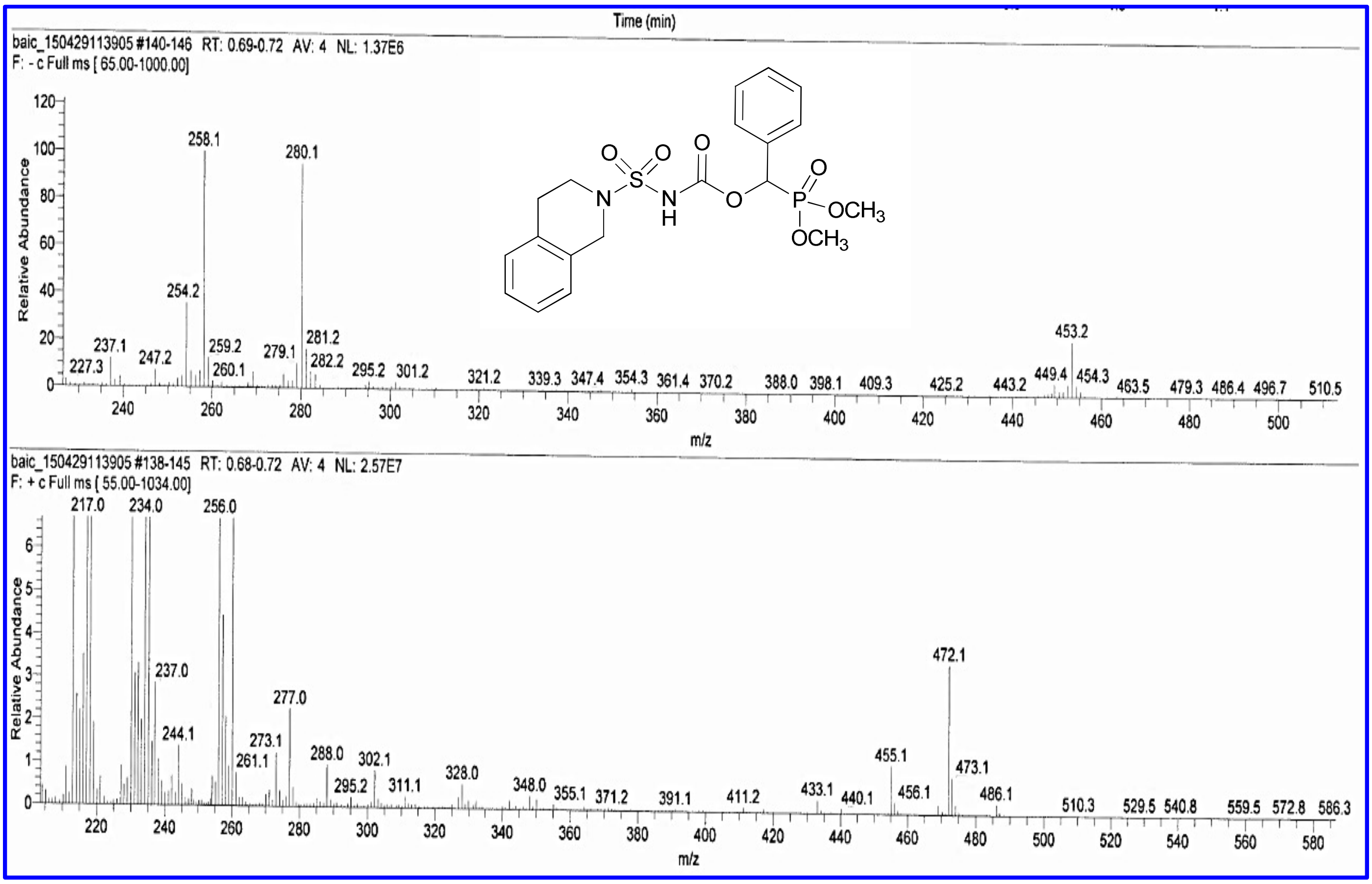

MS: (Dimethoxyphosphoryl)(phenyl)methyl (3,4-dihydroisoquinolin-2(1*H*)-yl)sulfonylcarbamate **6a**

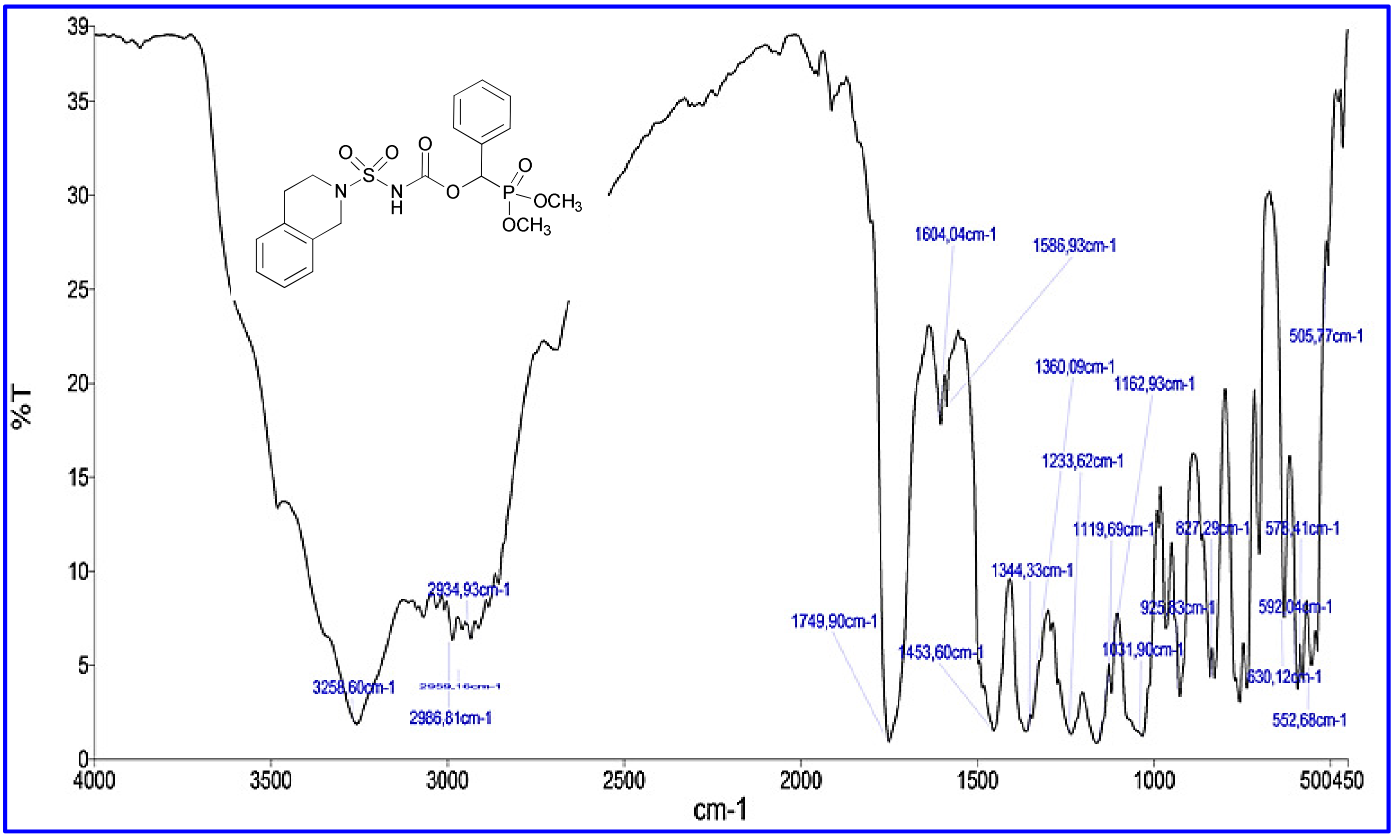

IR Spectrum: (Dimethoxyphosphoryl)(phenyl)methyl (3,4-dihydroisoquinolin-2(1*H*)-yl)sulfonylcarbamate **6a**

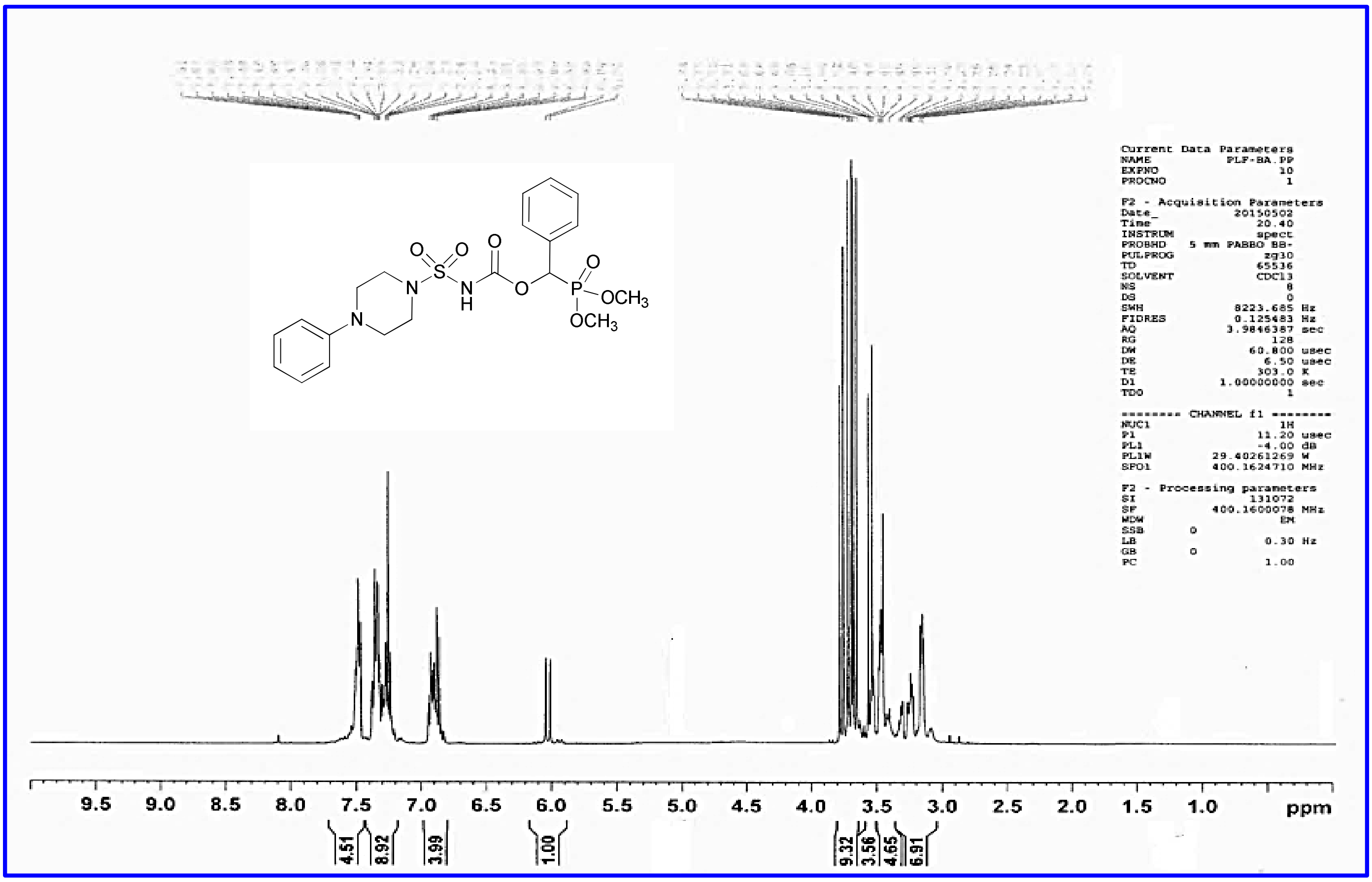

^{1}H NMR Spectrum: (Dimethoxyphosphoryl)(phenyl)methyl(4-phenylpiperazin-1-yl)sulfonylcarbamate **7a**

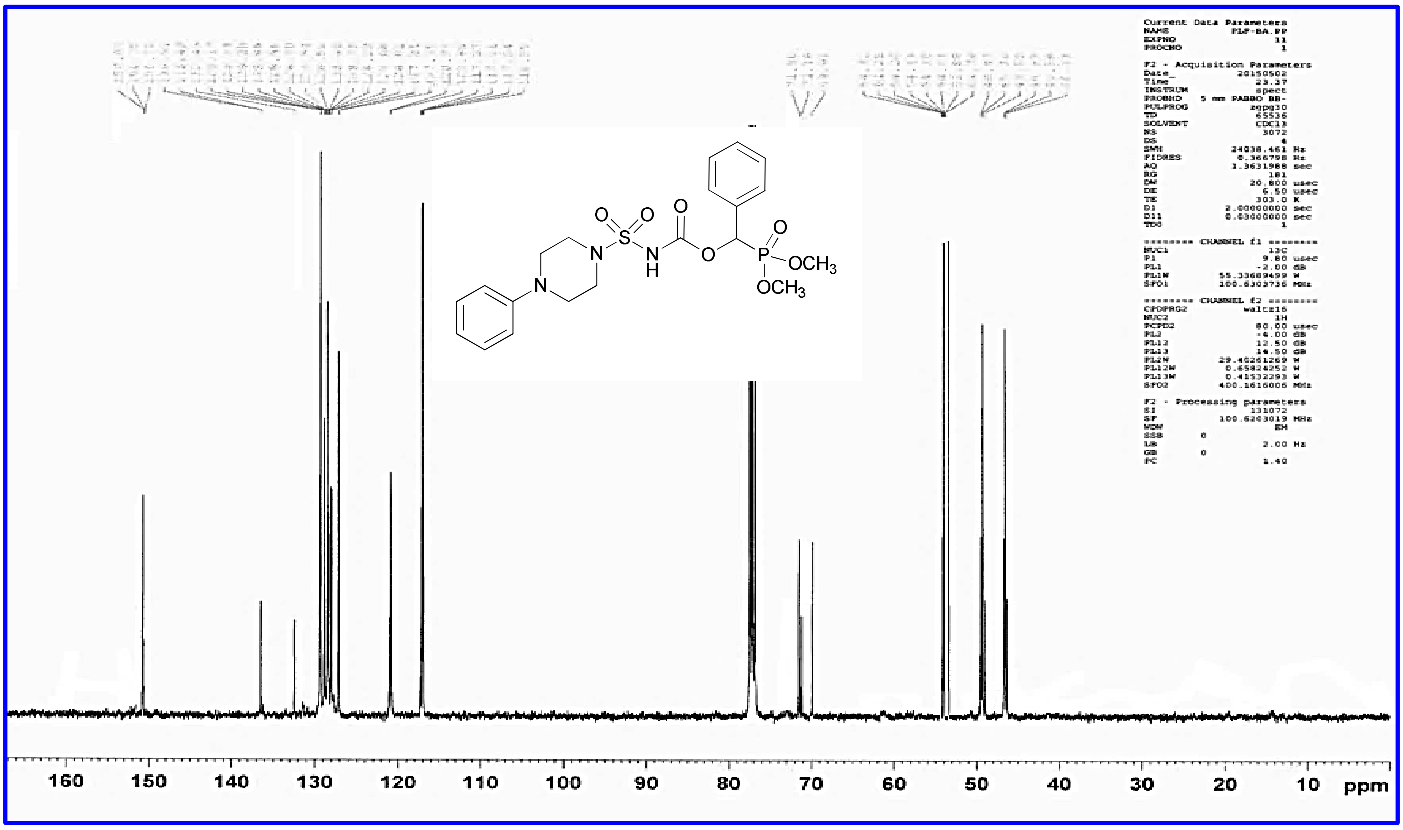

^{13}C NMR Spectrum: (Dimethoxyphosphoryl)(phenyl)methyl(4-phenylpiperazin-1-yl)sulfonylcarbamate **7a**

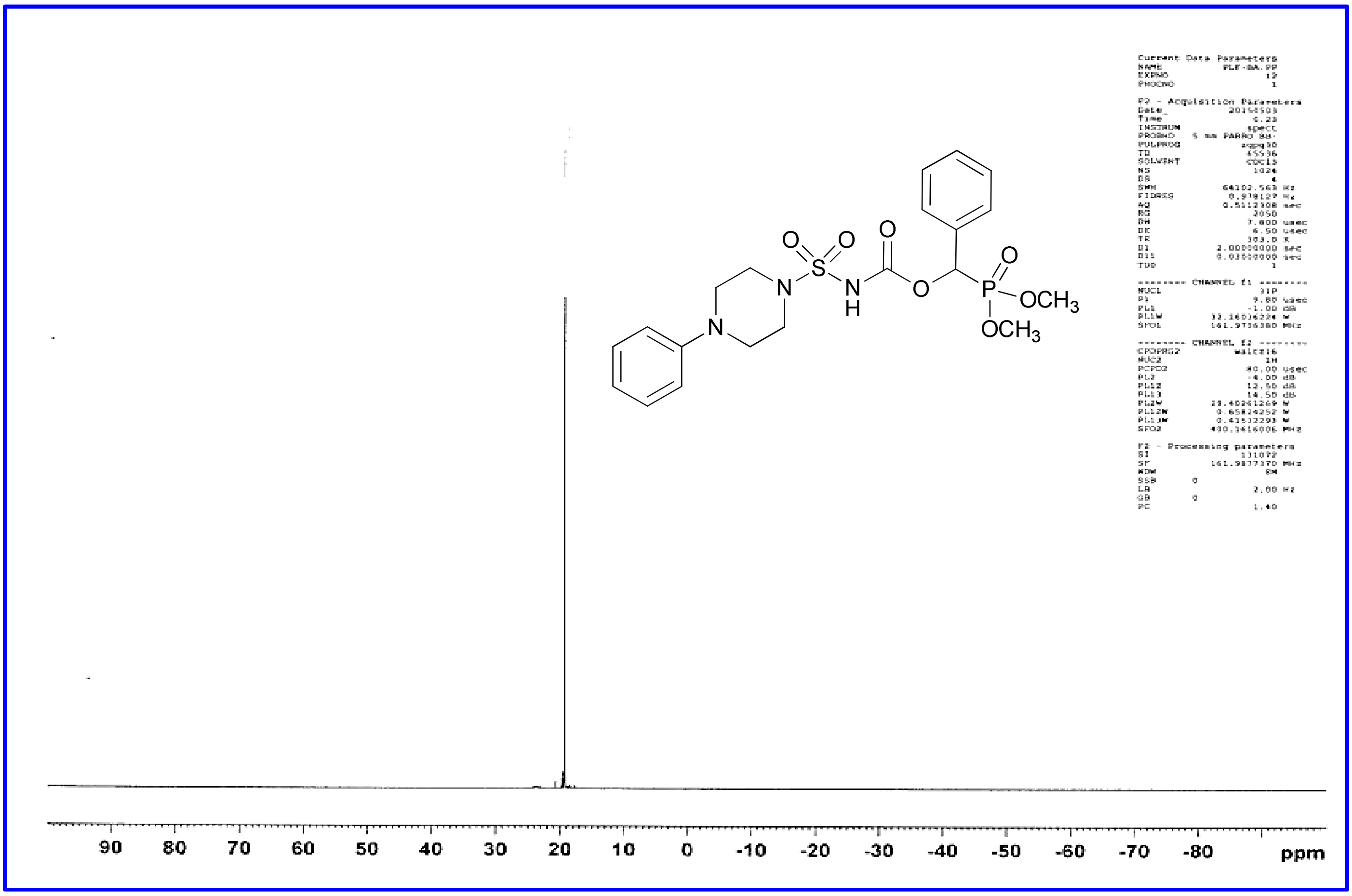

^{31}P NMR Spectrum: (Dimethoxyphosphoryl)(phenyl)methyl(4-phenylpiperazin-1-yl)sulfonylcarbamate **7a**

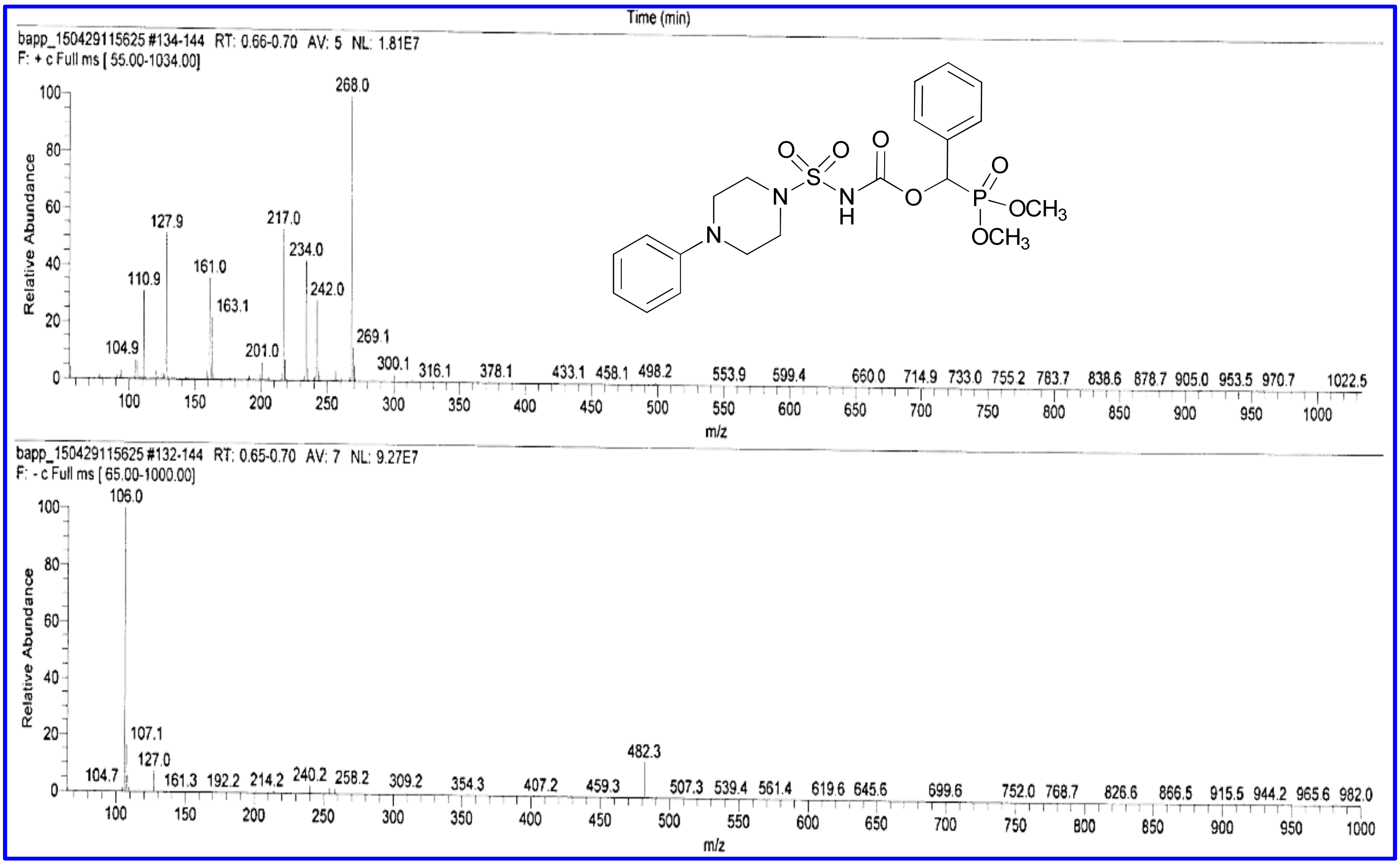

MS: (Dimethoxyphosphoryl)(phenyl)methyl(4-phenylpiperazin-1-yl)sulfonylcarbamate **7a**

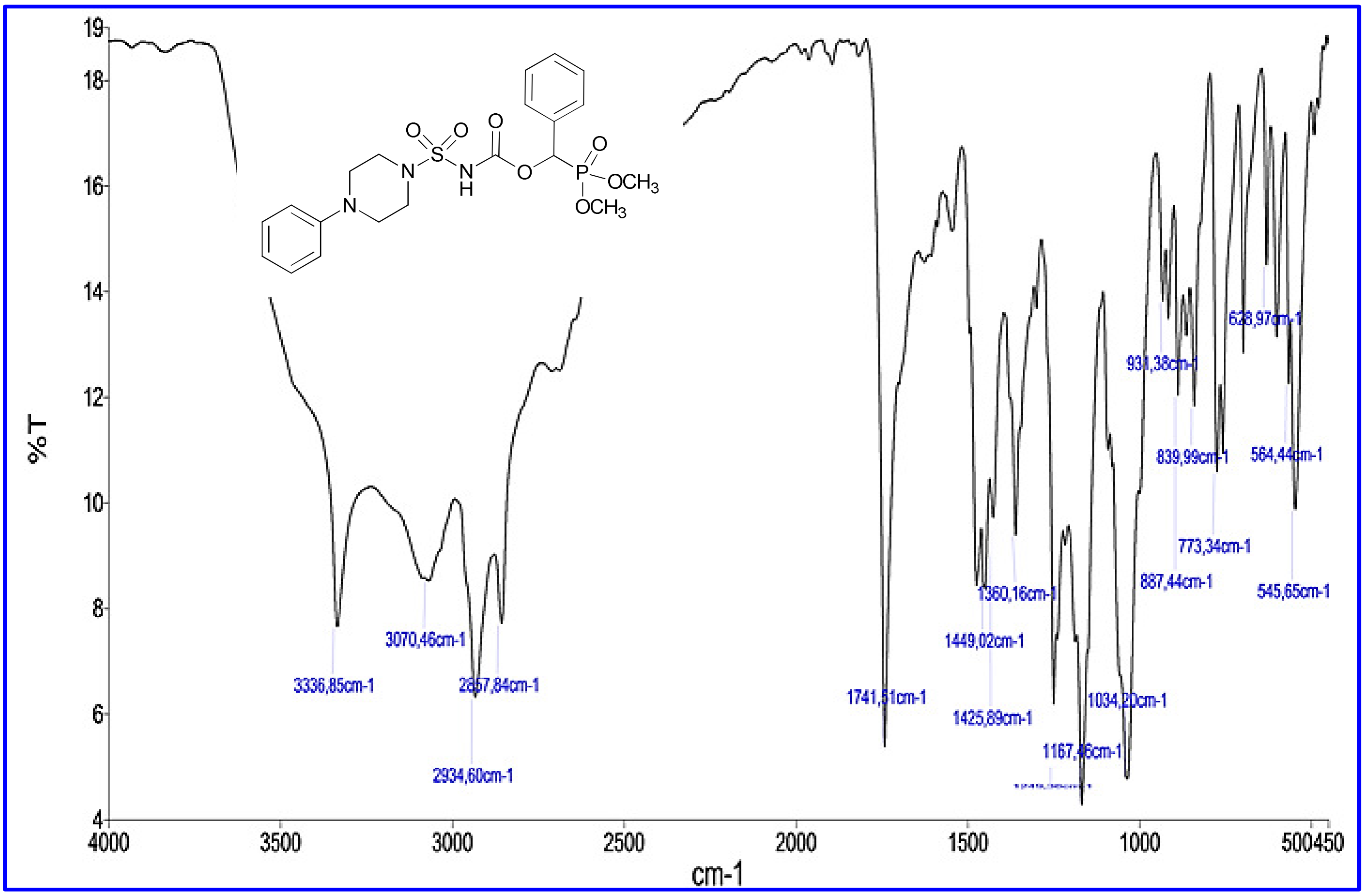

IR Spectrum: (Dimethoxyphosphoryl)(phenyl)methyl(4-phenylpiperazin-1-yl)sulfonylcarbamate **7a**

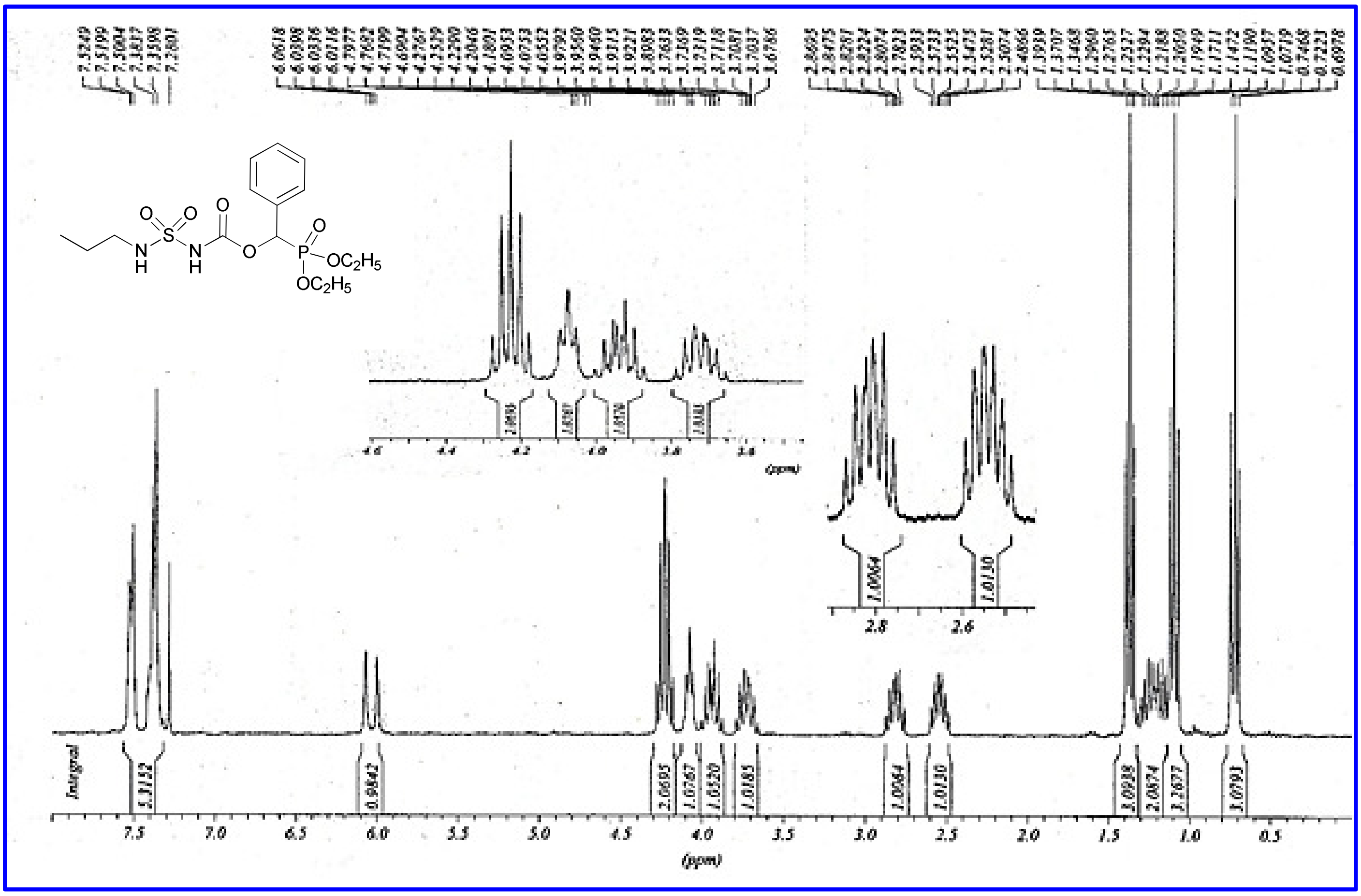

^{1}H NMR Spectrum: (diethoxyphosphoryl)(phenyl)methyl *N*-propylsulfamoylcarbamate **8a**

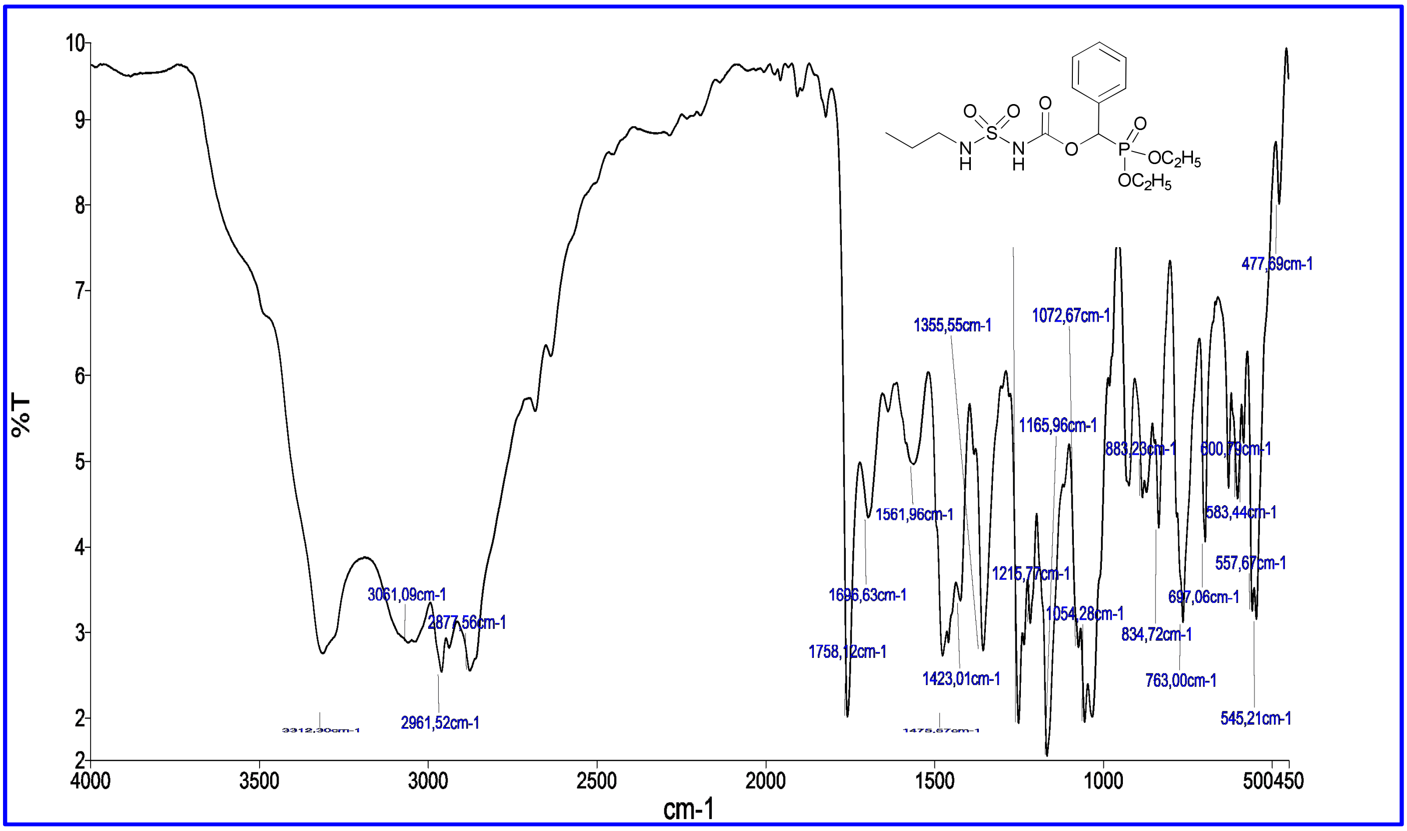

IR Spectrum: (diethoxyphosphoryl)(phenyl)methyl *N*-propylsulfamoylcarbamate **8a**

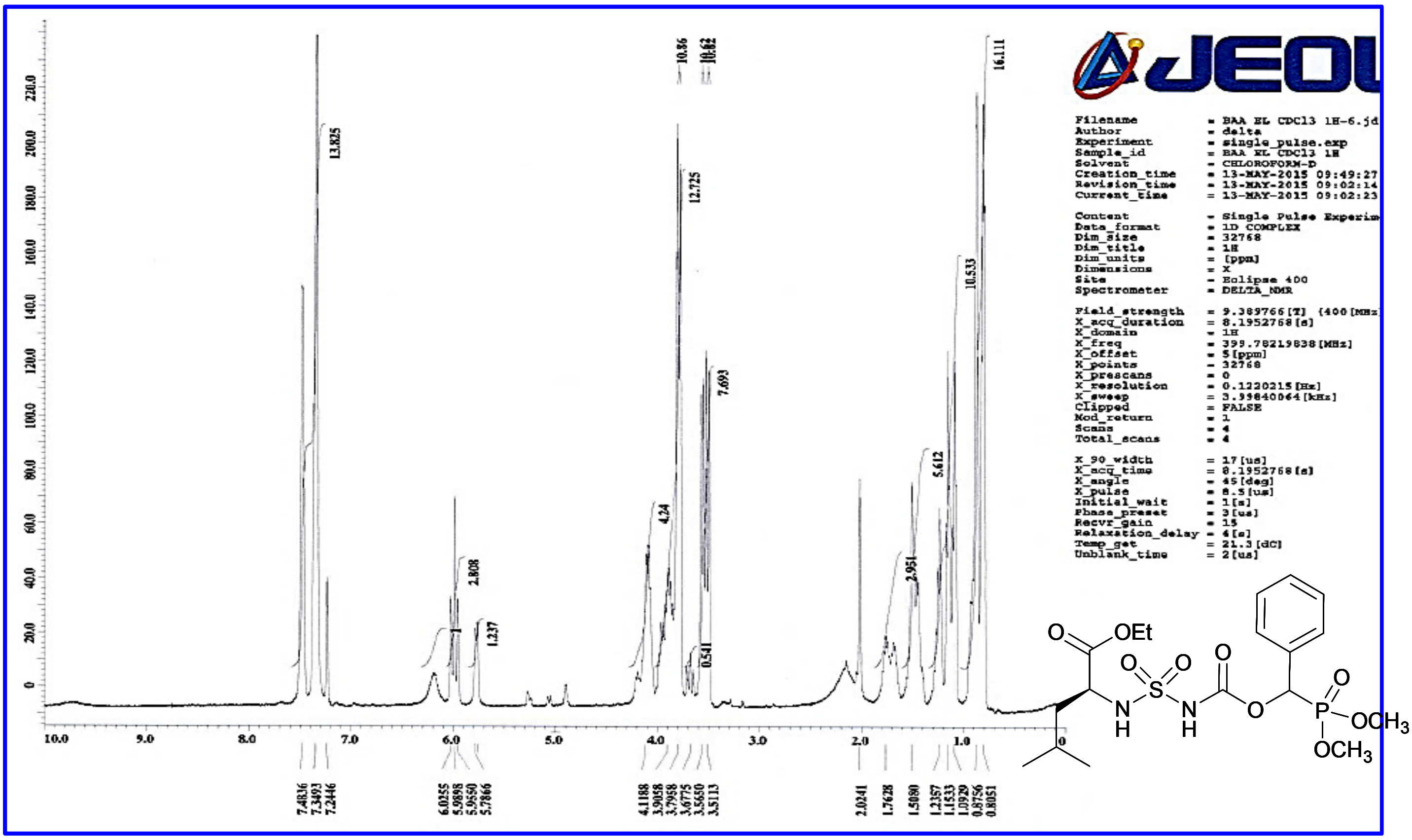

^{1}H NMR Spectrum: ethyl 2-((*N*-(((dimethoxyphosphoryl)(phenyl)methoxy)carbonyl)sulfamoyl)amino)-4-methylpentanoate **1b**

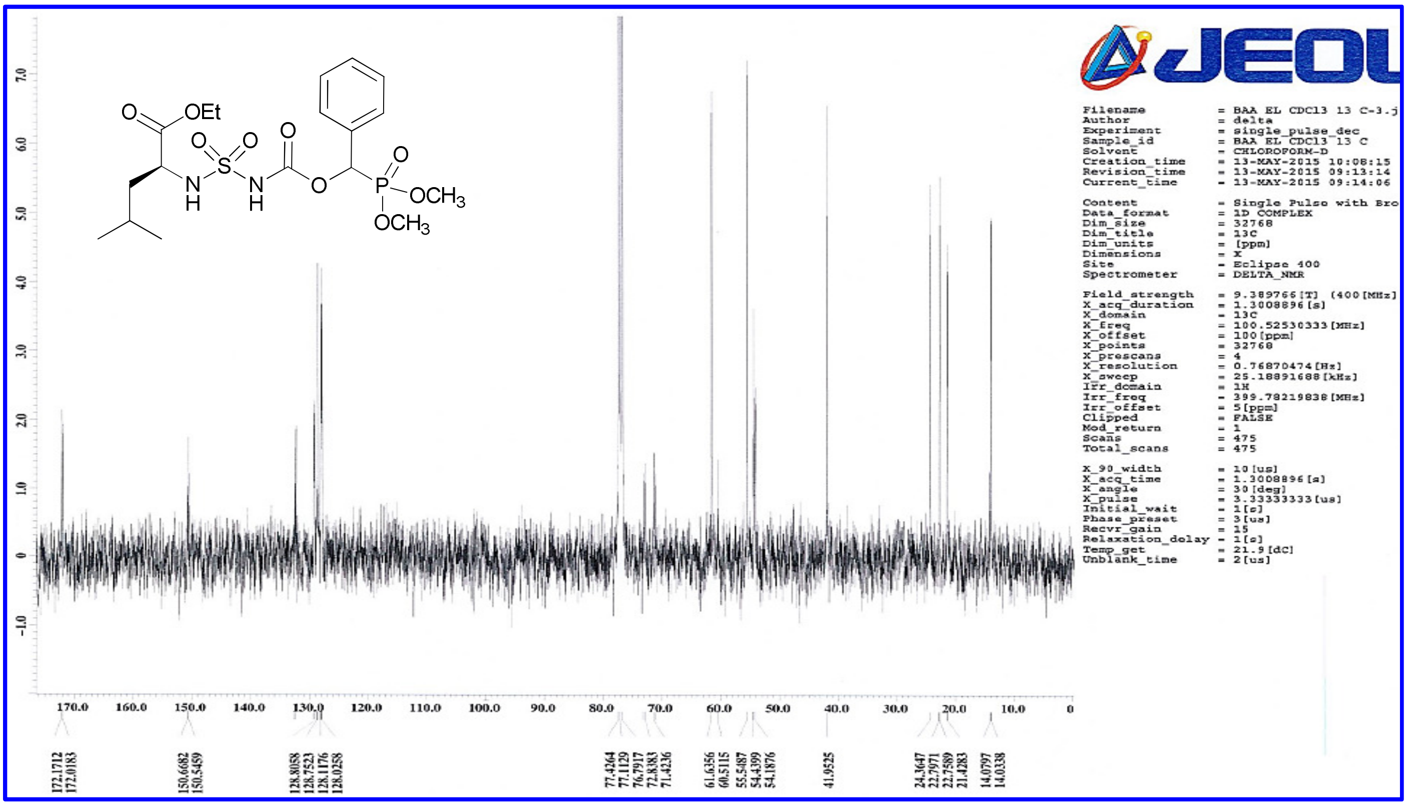

^{13}C NMR Spectrum: ethyl 2-((*N*-(((dimethoxyphosphoryl)(phenyl)methoxy)carbonyl)sulfamoyl)amino)-4-methylpentanoate **1b**

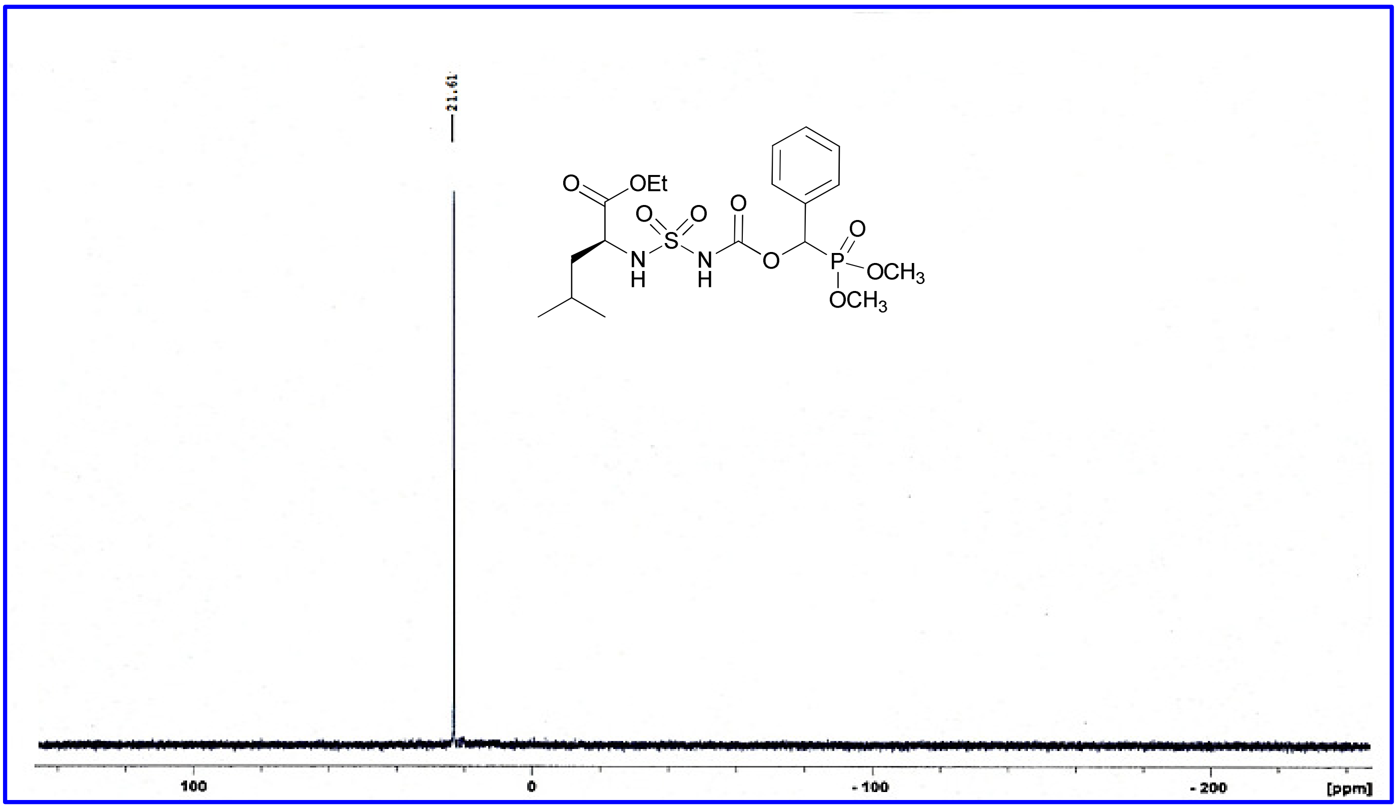

^{31}P NMR Spectrum: ethyl 2-((*N*-(((dimethoxyphosphoryl)(phenyl)methoxy)carbonyl)sulfamoyl)amino)-4-methylpentanoate **1b**

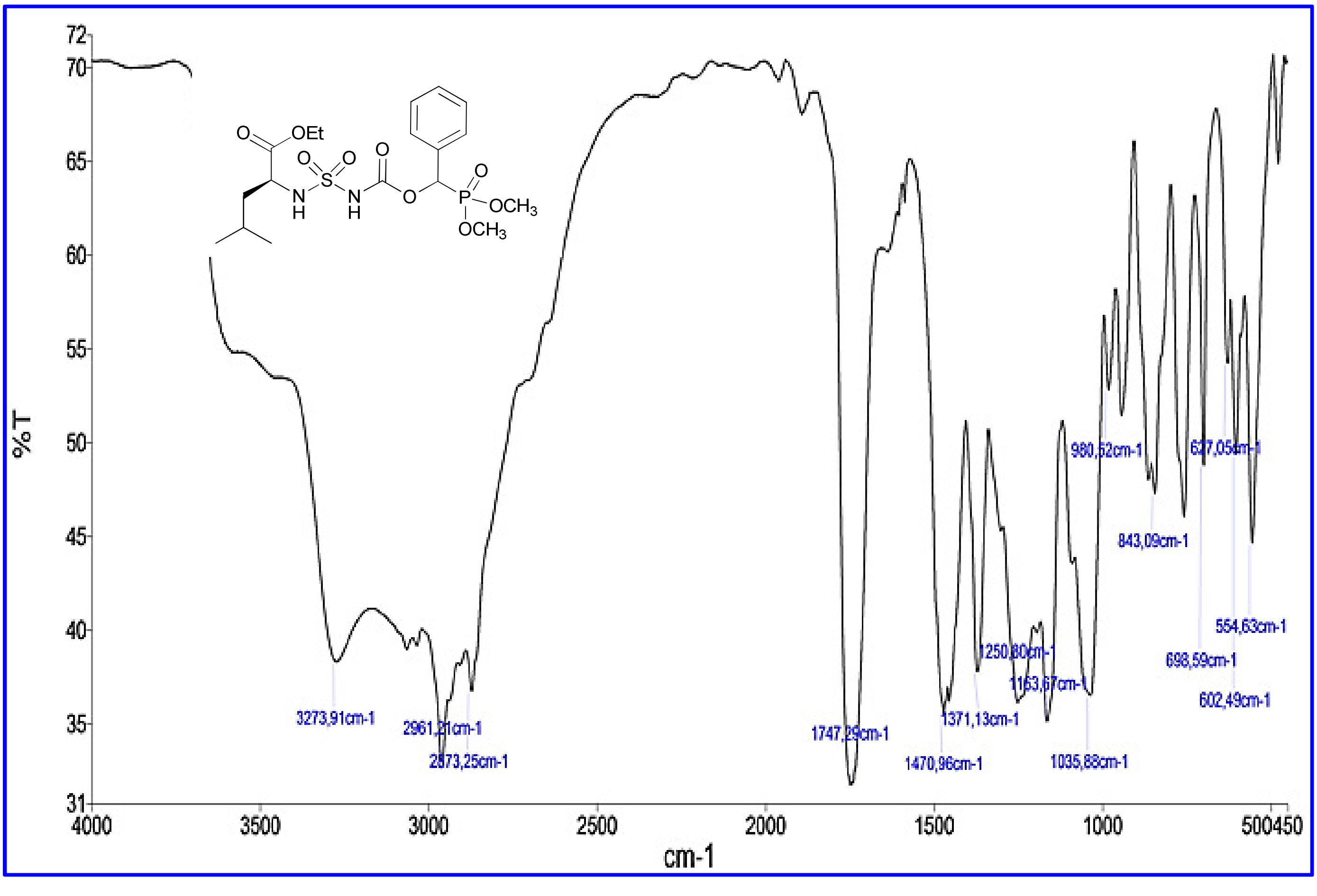

IR Spectrum: ethyl 2-((*N*-(((dimethoxyphosphoryl)(phenyl)methoxy)carbonyl)sulfamoyl)amino)-4-methylpentanoate **1b**

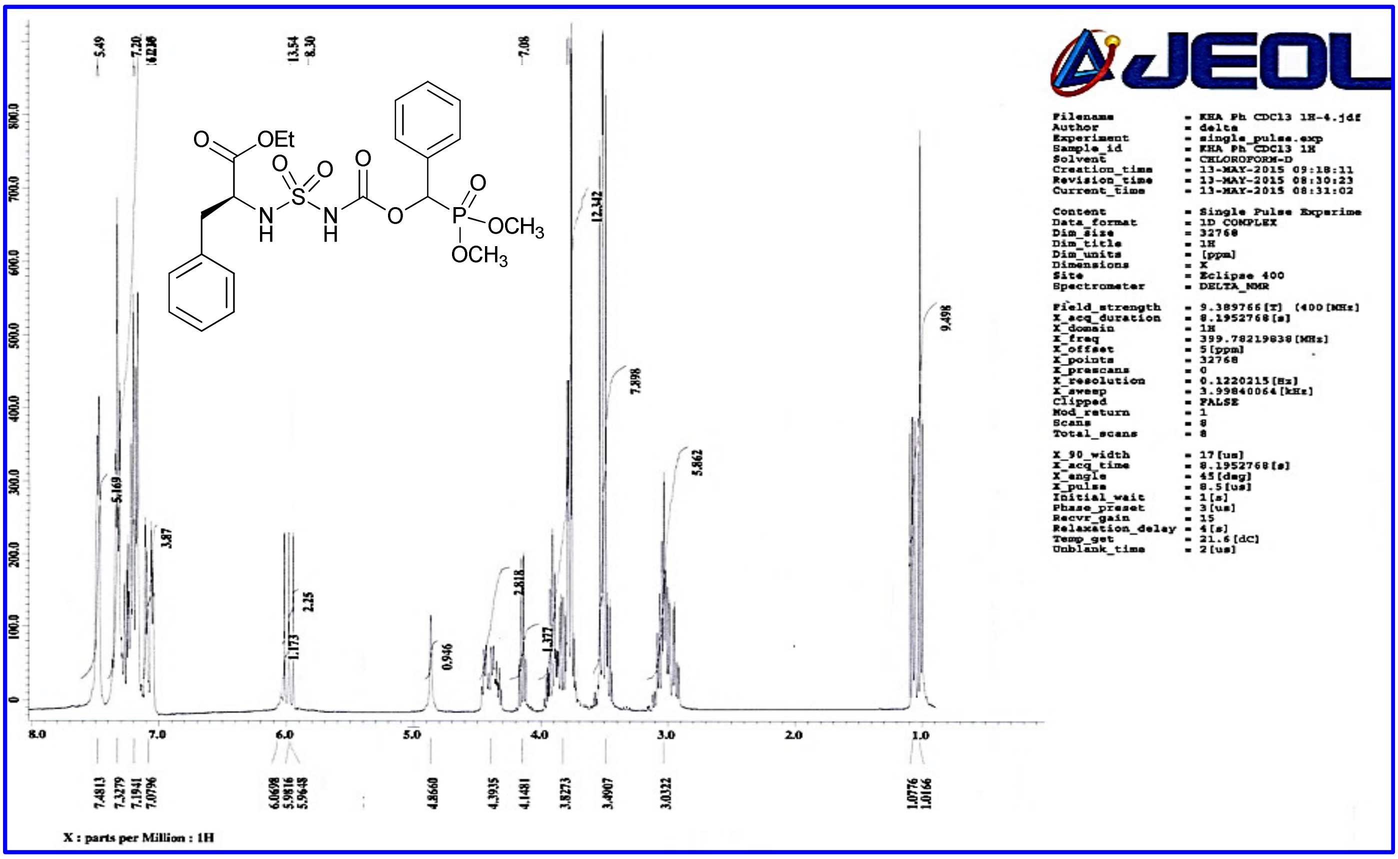

^{1}H NMR Spectrum: ethyl 2-((*N*-(((dimethoxyphosphoryl)(phenyl)methoxy)carbonyl)sulfamoyl)amino)-3-phenylpropanoate **2b**

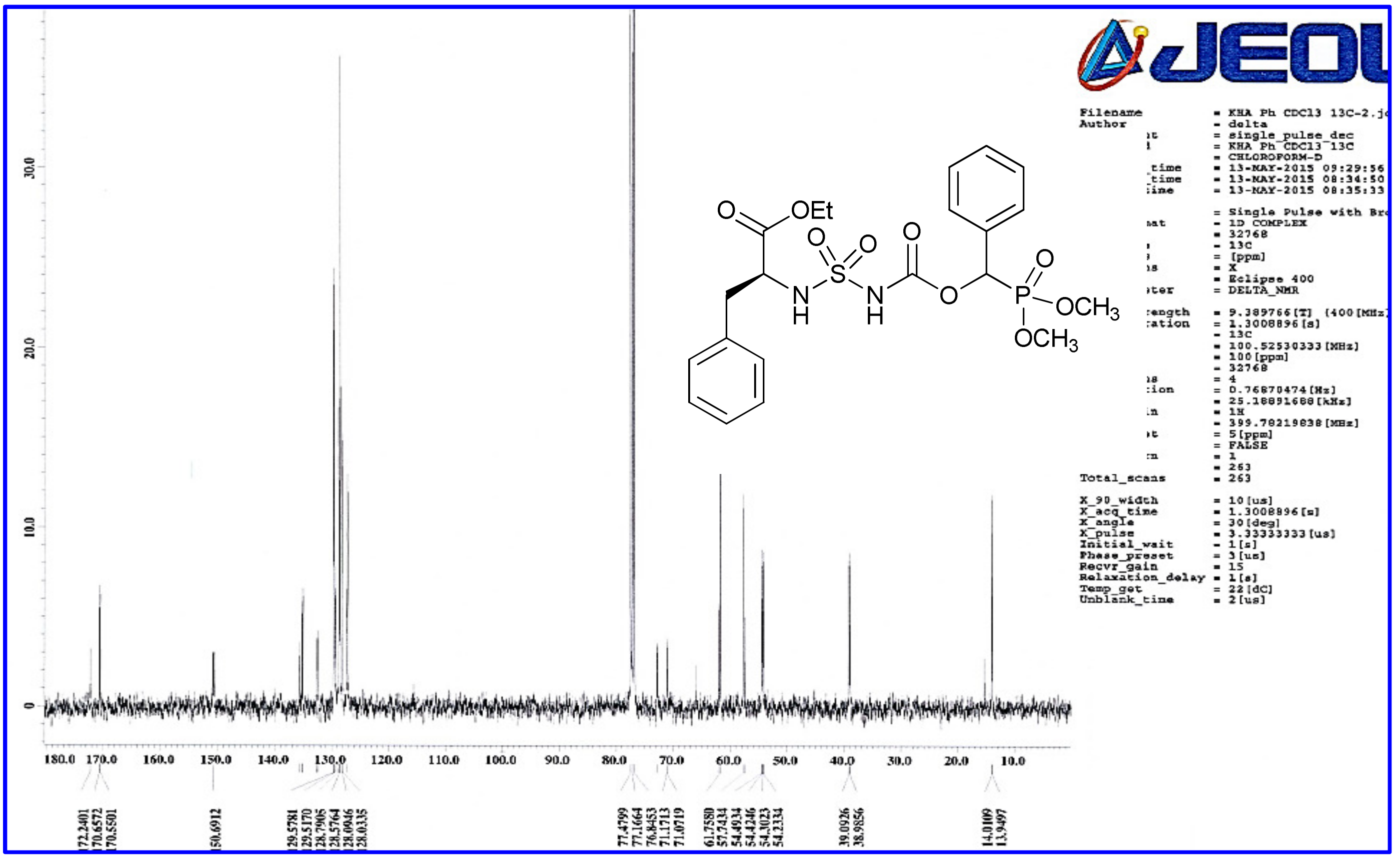

^{13}C NMR Spectrum: ethyl 2-((*N*-(((dimethoxyphosphoryl)(phenyl)methoxy)carbonyl)sulfamoyl)amino)-3-phenylpropanoate **2b**

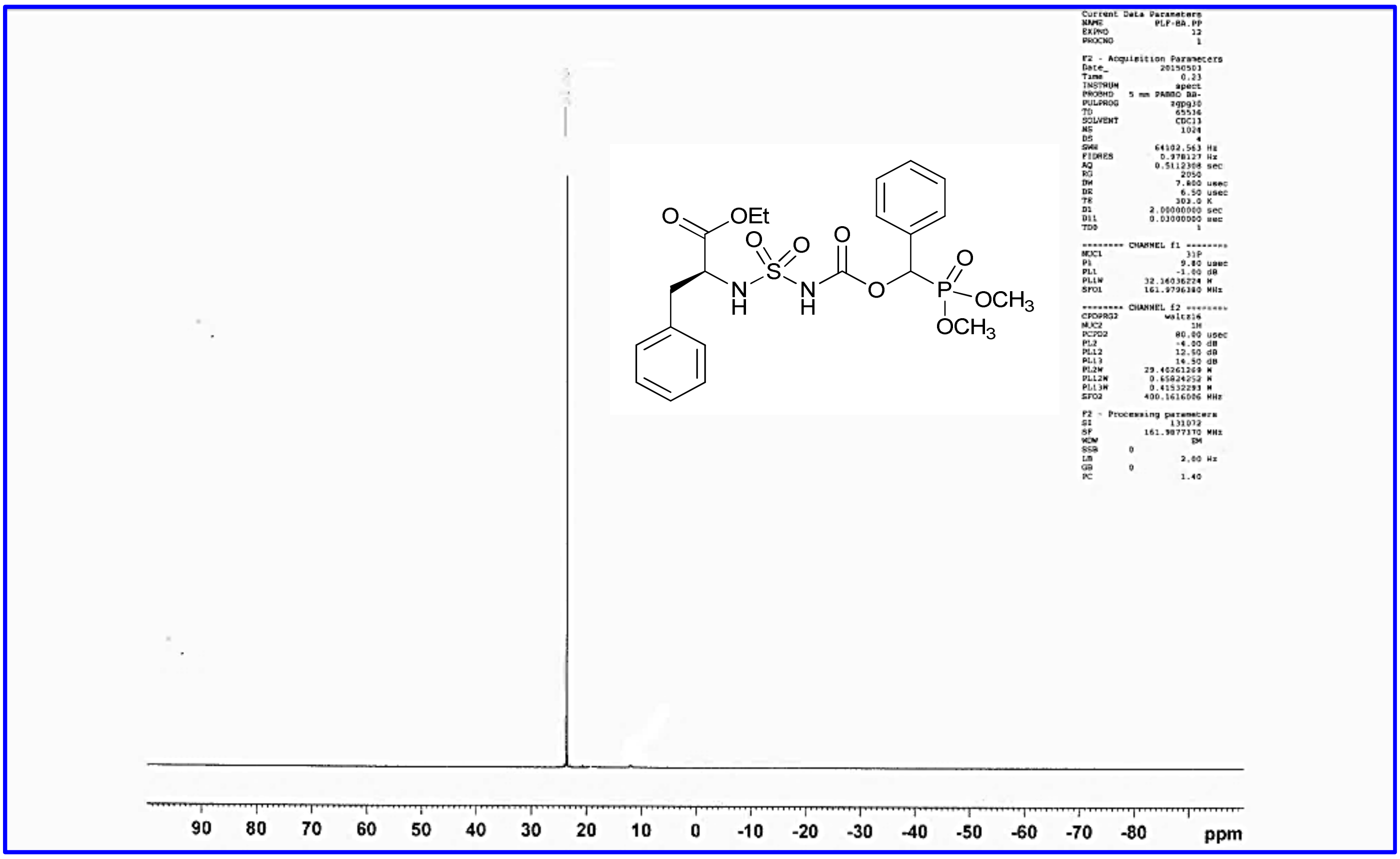

^{31}P NMR Spectrum: ethyl 2-((*N*-(((dimethoxyphosphoryl)(phenyl)methoxy)carbonyl)sulfamoyl)amino)-3-phenylpropanoate **2b**

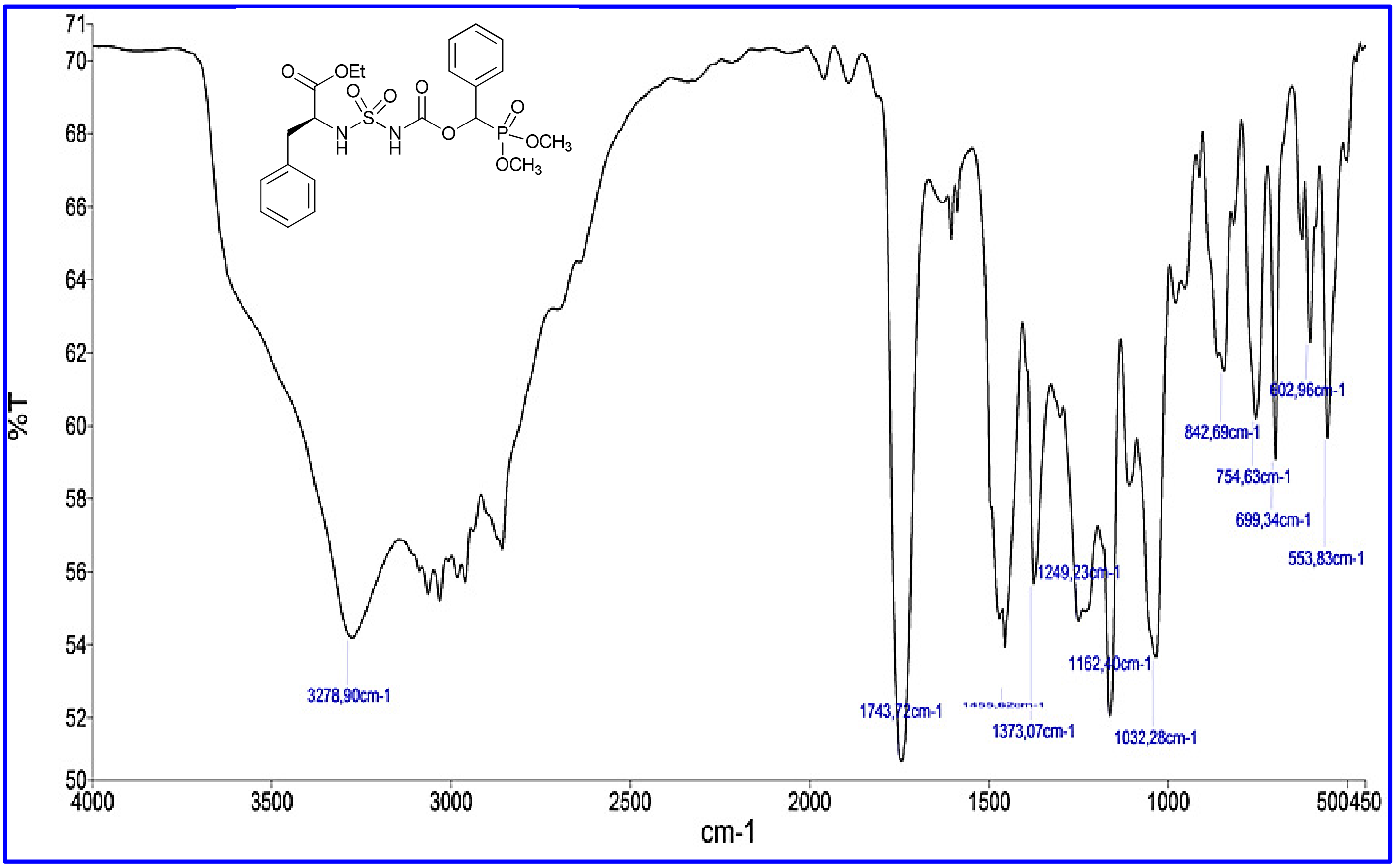

IR Spectrum: ethyl 2-((*N*-(((dimethoxyphosphoryl)(phenyl)methoxy)carbonyl)sulfamoyl)amino)-3-phenylpropanoate **2b**

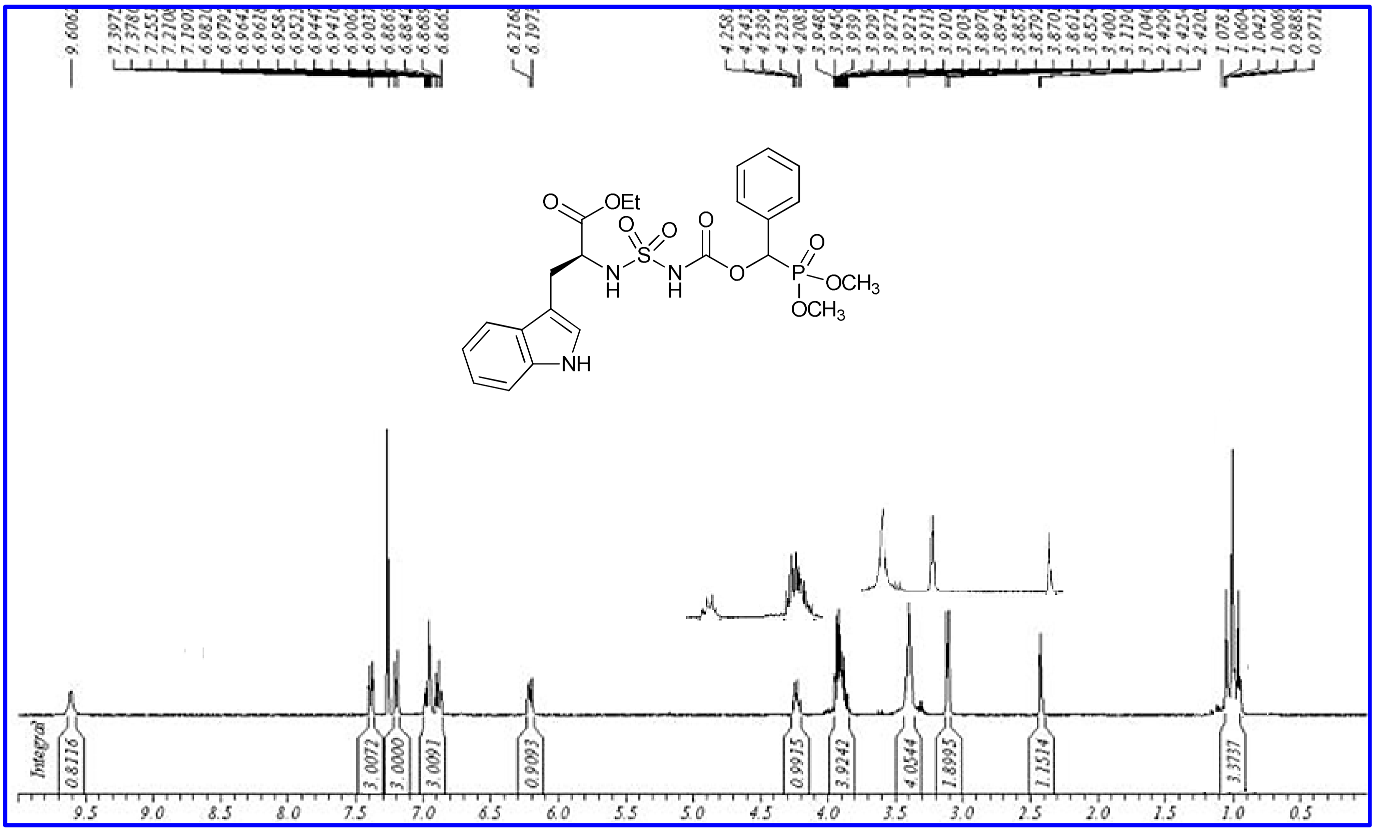

^{1}H NMR Spectrum: ethyl 2-((*N*-(((dimethoxyphosphoryl)(phenyl)methoxy)carbonyl)sulfamoyl)amino)-3-(1*H*-indol-3-yl)propanoate **3b**

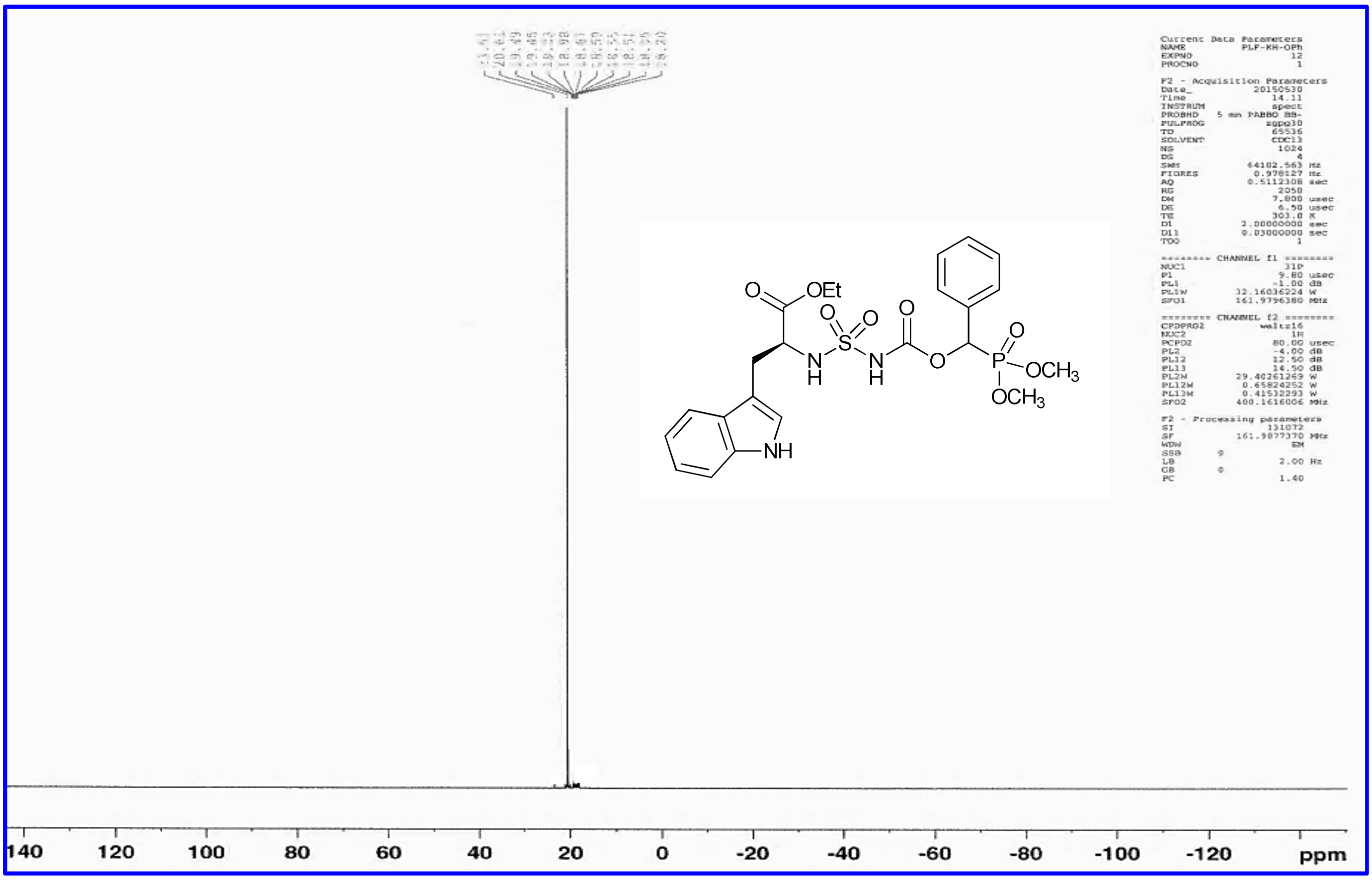

^{31}P NMR Spectrum: ethyl 2-((*N*-(((dimethoxyphosphoryl)(phenyl)methoxy)carbonyl)sulfamoyl)amino)-3-(1*H*-indol-3-yl)propanoate **3b**

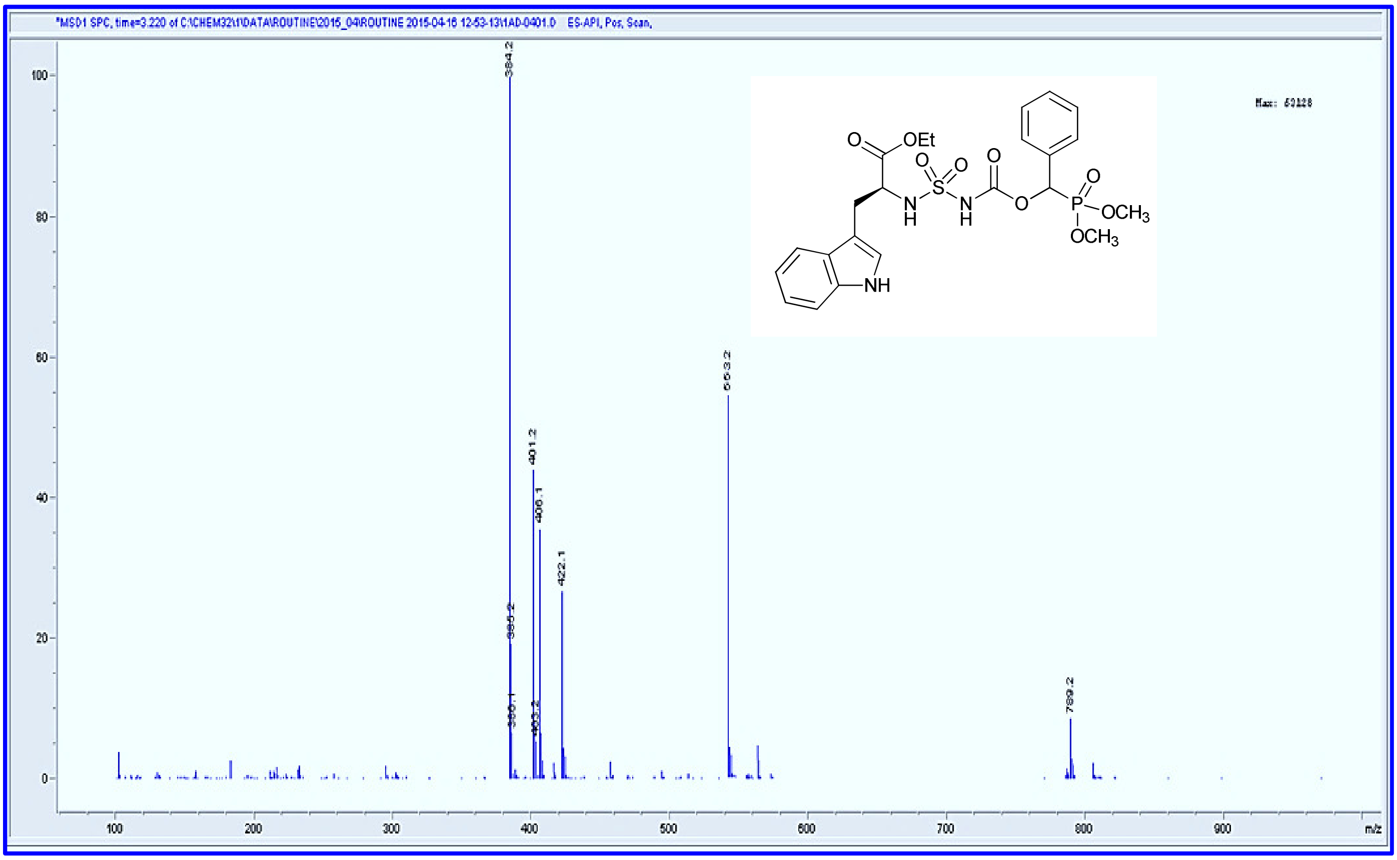

MS: ethyl 2-((*N*-(((dimethoxyphosphoryl)(phenyl)methoxy)carbonyl)sulfamoyl)amino)-3-(1*H*-indol-3-yl)propanoate **3b**

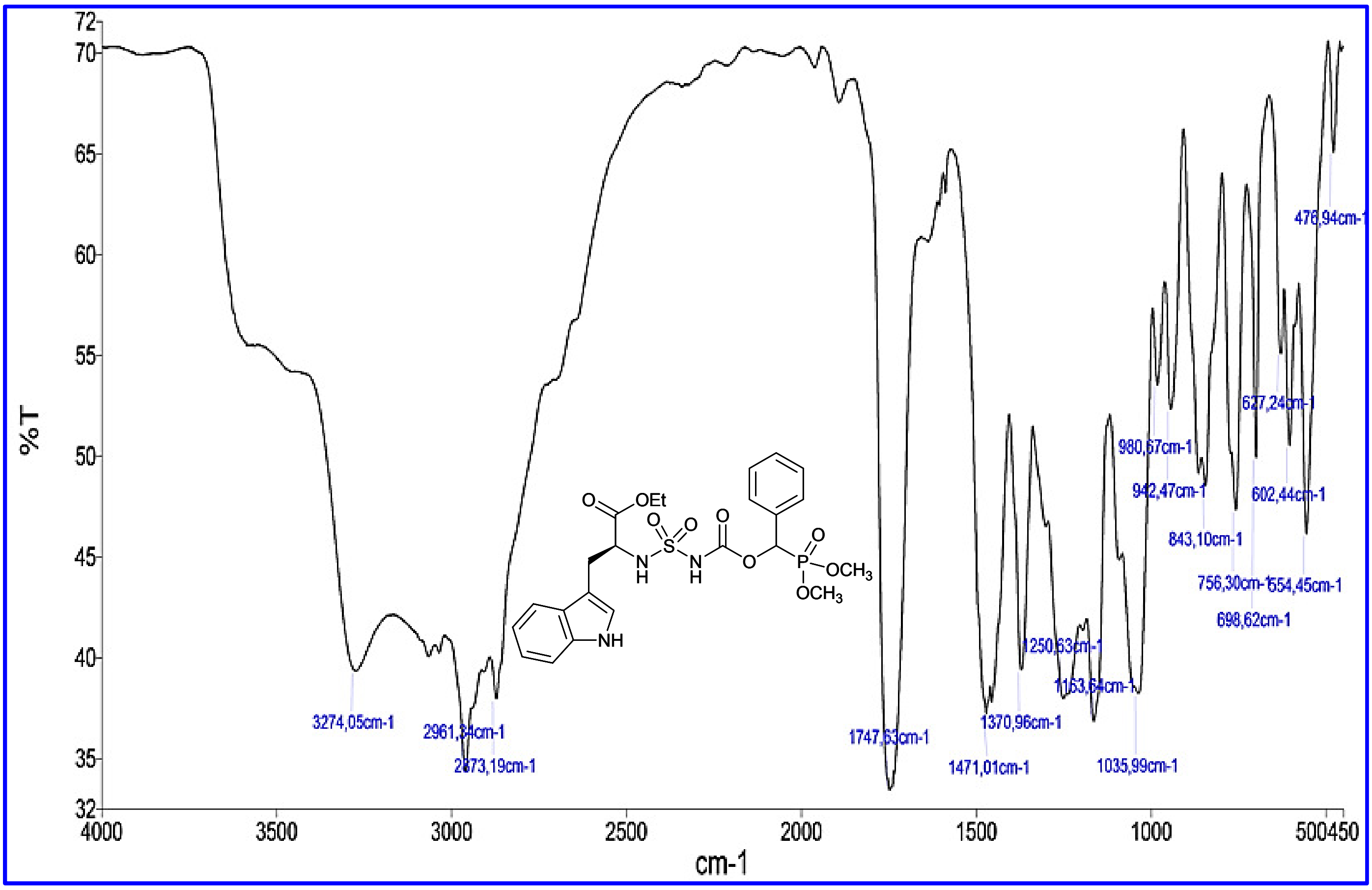

IR Spectrum: ethyl 2-((*N*-(((dimethoxyphosphoryl)(phenyl)methoxy)carbonyl)sulfamoyl)amino)-3-(1*H*-indol-3-yl)propanoate **3b**

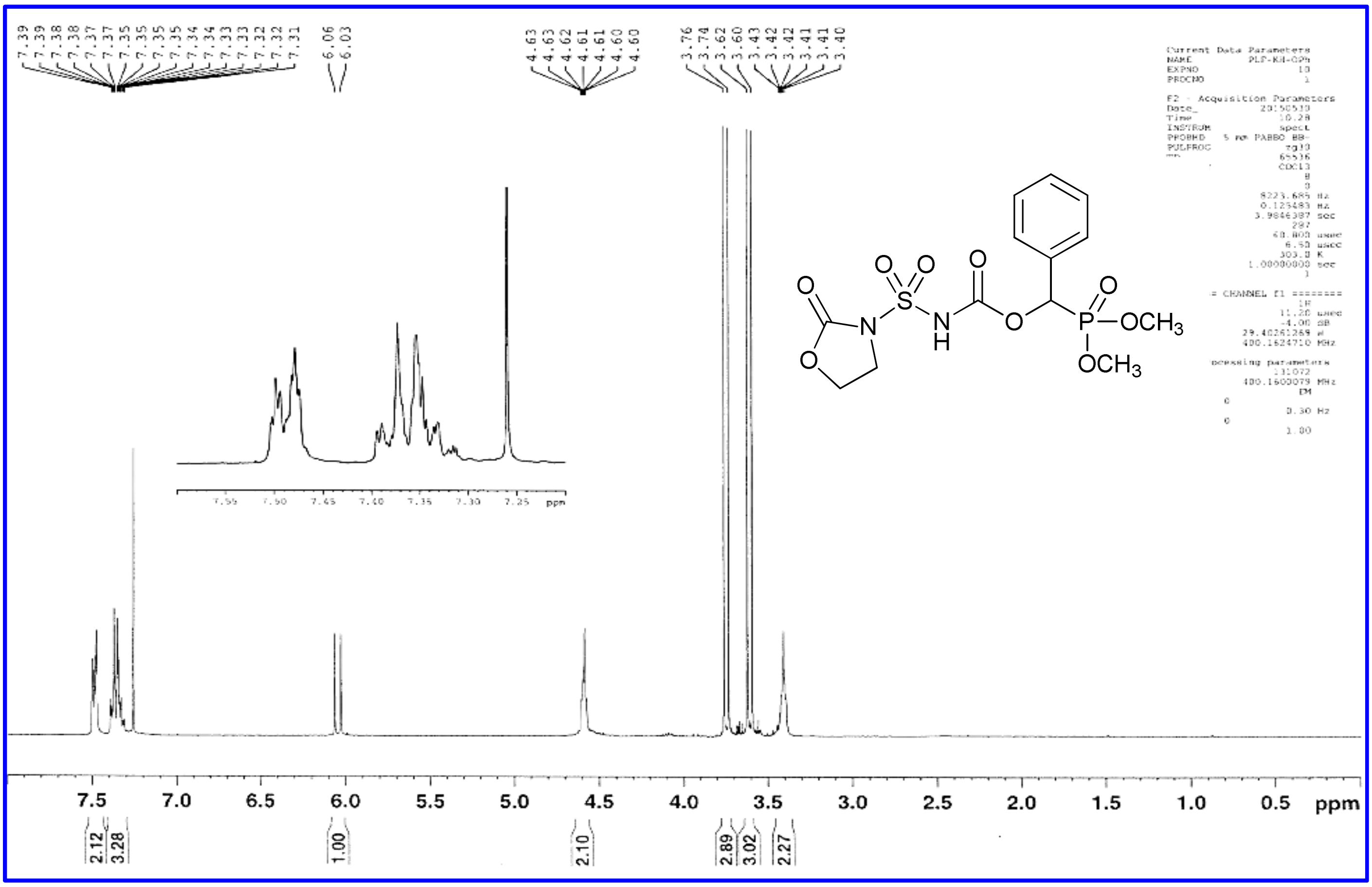

^{1}H NMR Spectrum: (Dimethoxyphosphoryl)(phenyl)methyl (2-oxooxazolidin-3-yl)sulfonylcarbamate **1c**

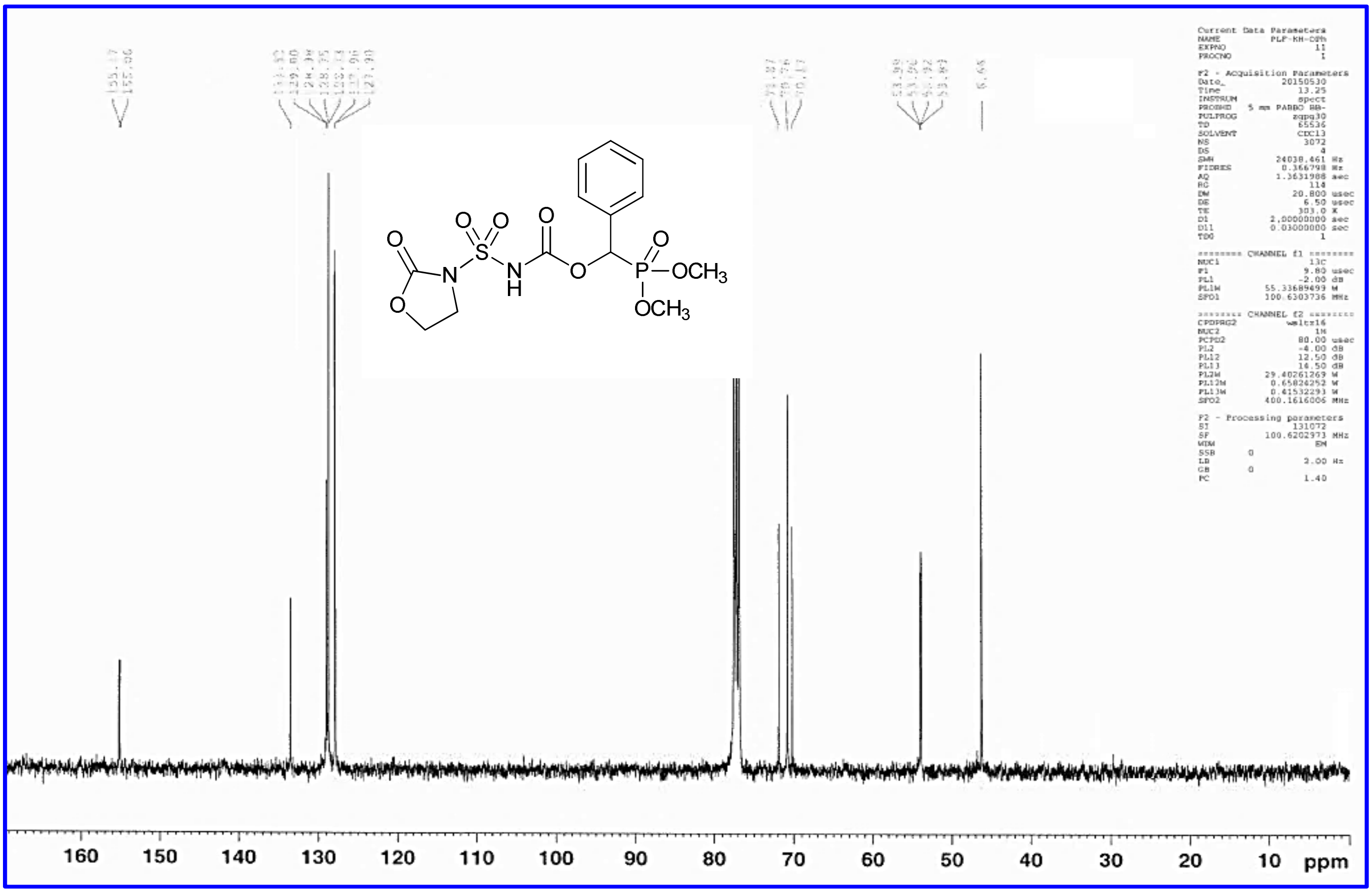

^{13}C NMR Spectrum: (Dimethoxyphosphoryl)(phenyl)methyl((2-oxooxazolidin-3-yl)sulfonyl)carbamate **1c**

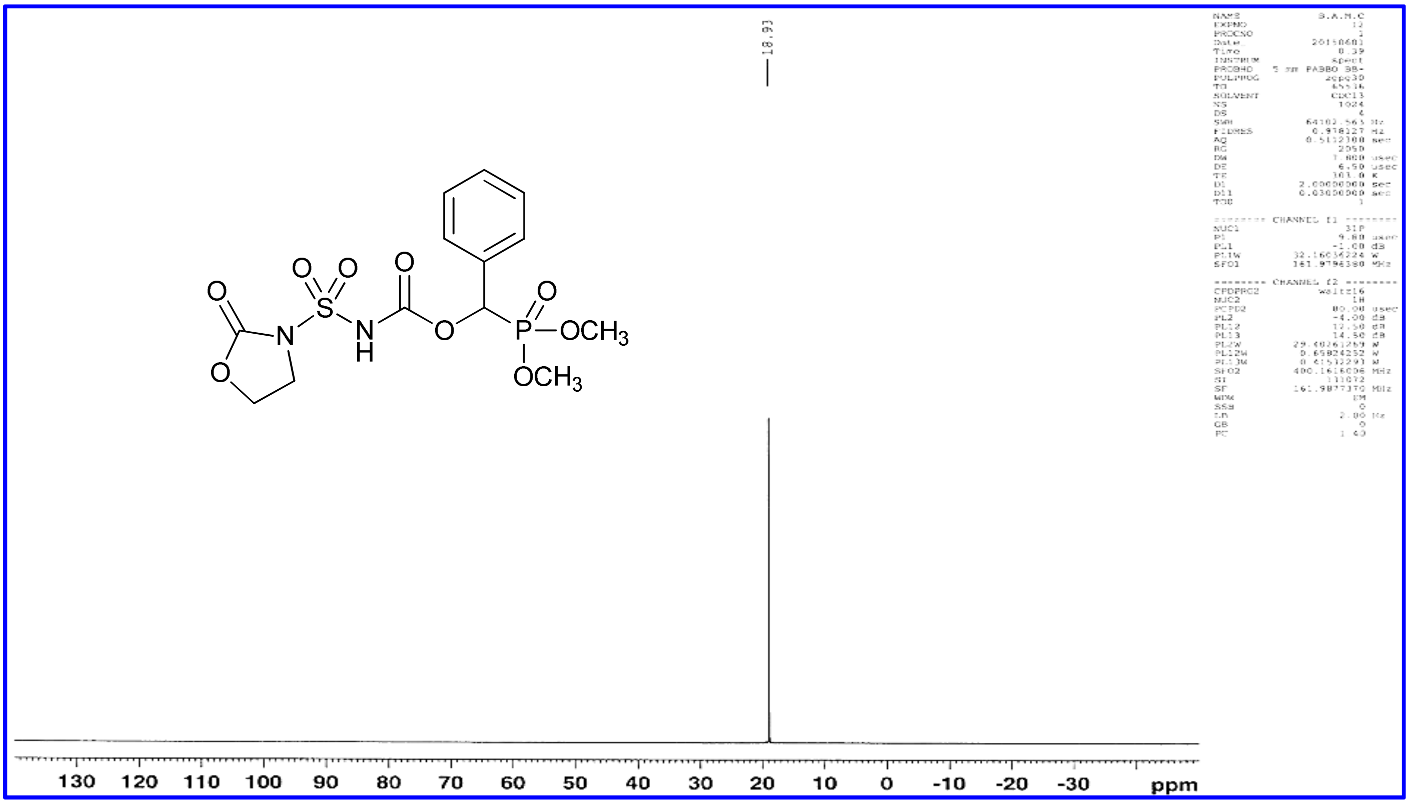

^{31}P NMR Spectrum: (Dimethoxyphosphoryl)(phenyl)methyl((2-oxooxazolidin-3-yl)sulfonyl)carbamate **1c**

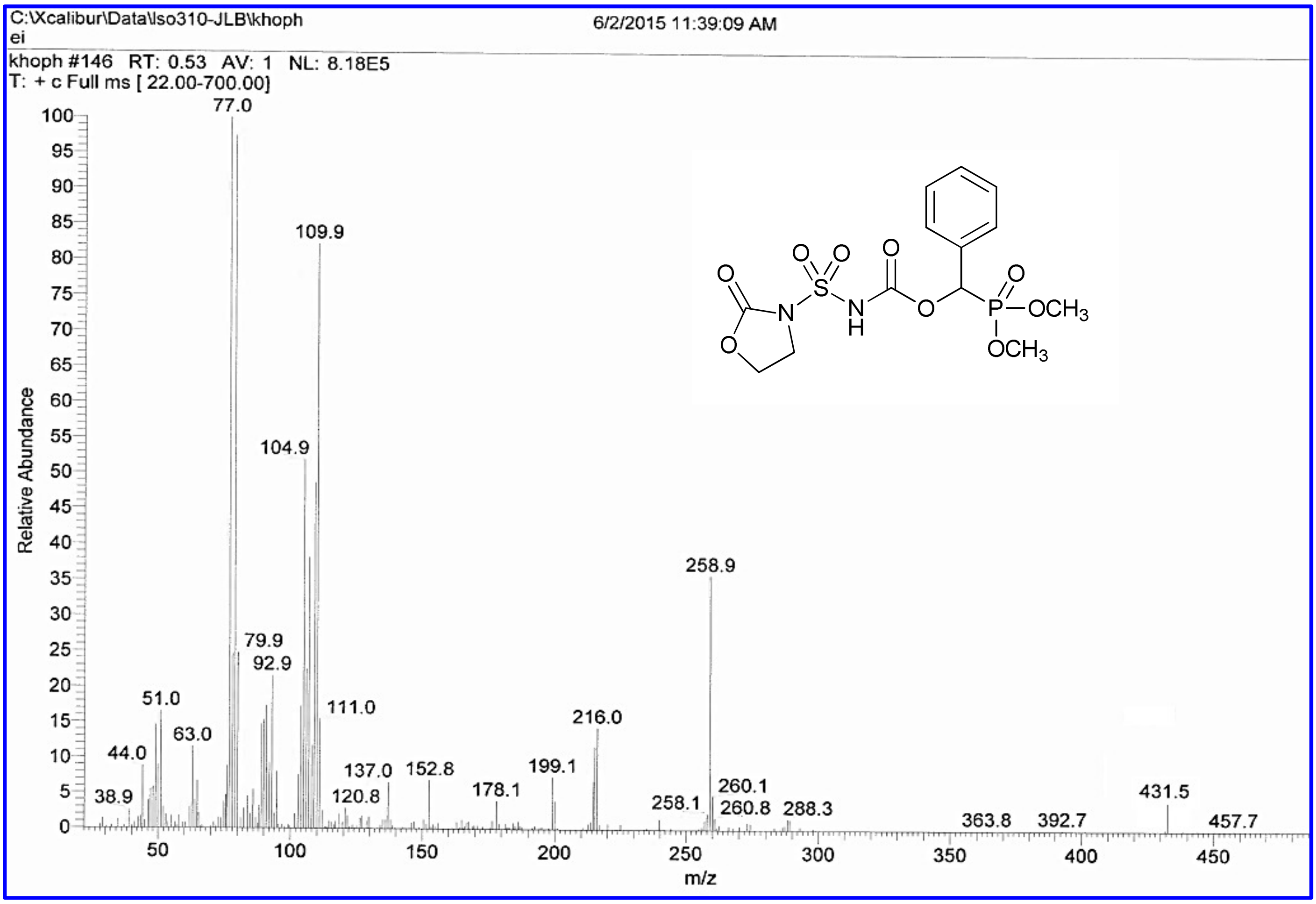

MS: (Dimethoxyphosphoryl)(phenyl)methyl((2-oxooxazolidin-3-yl)sulfonyl)carbamate **1c**

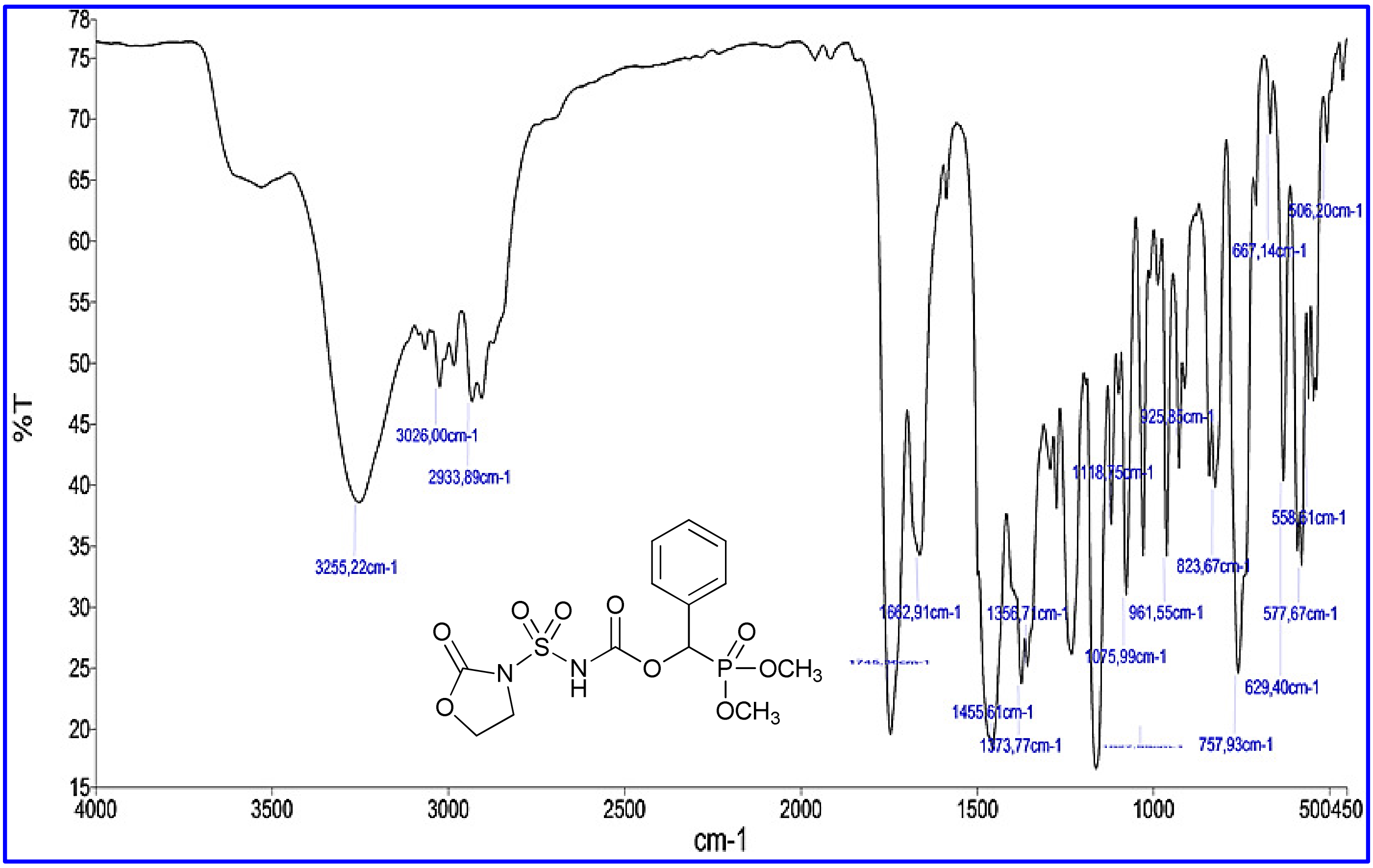

IR Spectrum: (Dimethoxyphosphoryl)(phenyl)methyl((2-oxooxazolidin-3-yl)sulfonyl)carbamate **1c**
